# Macrocarpane-Type Sesquiterpenes from *Laurencia microcladia*

**DOI:** 10.3390/md24090316

**Published:** 2026-09-09

**Authors:** Adrián Gutiérrez-Cepeda, Jorge J. Cabrera-Trujillo, José J. Fernández, Antonio Hernández-Daranas, Manuel Norte, María L. Souto

**Affiliations:** 1 Instituto Universitario de Bio-Orgánica Antonio González (IUBO AG), Universidad de La Laguna (ULL), Avenida Astrofísico Francisco Sánchez 2, 38206 La Laguna, Spain; aguticep@gmail.com (A.G.-C.); jcabrert@ull.edu.es (J.J.C.-T.); mnorte@ull.edu.es (M.N.); 2 Department of Chemistry, Chemistry Institute, Sciences Faculty, Autonomous University of Santo Domingo, University City, Santo Domingo 1355, Dominican Republic; 3 Departamento de Química Orgánica, Universidad de La Laguna (ULL), Avenida Astrofísico Francisco Sánchez 3, 38206 La Laguna, Spain; 4 Biotecnología Marina, IUBO AG-ULL, Unidad Asociada al IPNA-CSIC, 38206 La Laguna, Spain; 5 Instituto de Productos Naturales y Agrobiología (IPNA), Consejo Superior de Investigaciones Científicas (CSIC), Avenida Astrofísico Francisco Sánchez 3, 38206 La Laguna, Spain; adaranas@ipna.csic.es

**Keywords:** macrocarpanes, sesquiterpenes, *Laurencia microcladia*, marine natural products, vanadium-dependent peroxidases, DFT, apoptosis-inducing activity

## Abstract

Seven undescribed sesquiterpenes featuring an uncommon macrocarpane skeleton, laurocarpanes A–G (**1**–**7**), were isolated from specimens of *Laurencia microcladia* collected in Fuerteventura, the Canary Islands. The identification of these macrocarpane-type metabolites is of particular interest from both chemical and chemotaxonomic perspectives, as it highlights distinctive biosynthetic capabilities within the studied species and further expands current knowledge regarding the distribution of this uncommon structural class in marine organisms. Their structures and configurational assignments were determined by a comprehensive NMR study, high-resolution mass spectrometry, and coupling constant evaluations. A biosynthetic model was proposed linking the assembly of the macrocarpane skeleton directly to an (*S*)-*β*-bisabolene monocyclic precursor, with the process mediated by vanadium-dependent bromoperoxidase (V-BPO). The plausibility of the proposed biogenetic formation of laurocarpanes A–G (**1**–**7**) was assessed through Density Functional Theory (DFT) calculations.

## 1. Introduction

**Figure 1:**
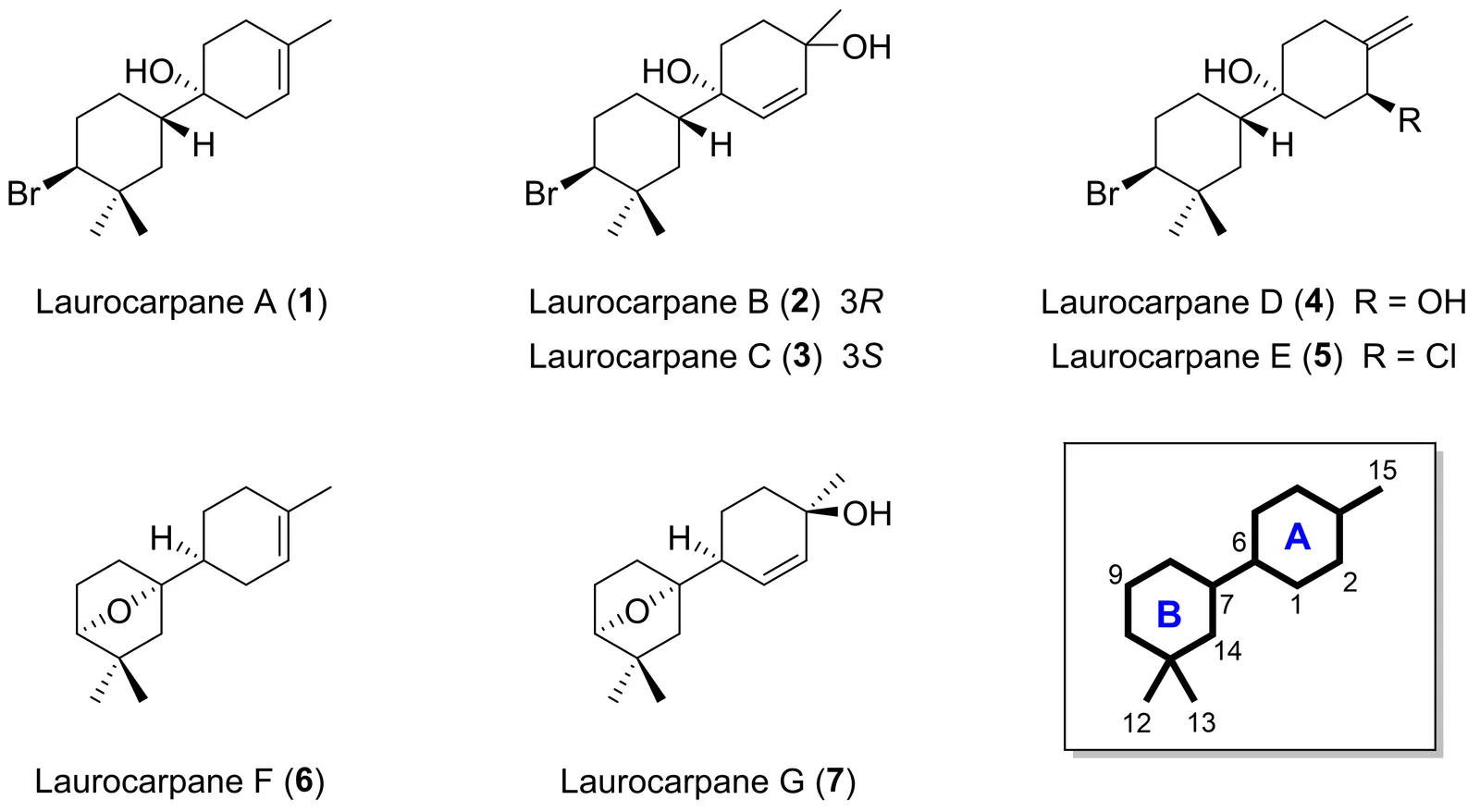
Structures of new sesquiterpenes isolated from the red alga *Laurencia microcladia*.

The bisabolyl-type cation plays a central role in the biosynthesis of various sesquiterpenes, acting as the precursor to several major skeletal types, such as cuparanes, lauranes, chamigranes, etc. [1]. Furthermore, cyclization between the C-14 and C-11 positions of the deprotonated bisabolyl cation intermediate is responsible for the formation of a rare class of bicyclic sesquiterpenes, the macrocarpanes (Figure 1). This skeleton was first proposed by Cool in 2005 [2] for a series of five new hydrocarbon sesquiterpenes isolated from the leaves of *Cupressus macrocarpa*, hence the common name for this class of compounds. The number of macrocarpane skeleton metabolites that have been isolated from natural sources is limited. In addition to eight examples obtained from terrestrial sources [2,3], only nine sesquiterpenes and one norderivative have been isolated, and all from *Laurencia* species [4,5,6].

As part of our continuing interest in the chemistry of the genus *Laurencia* [7,8,9,10,11,12,13] and during the course of a marine bioactive compound discovery program, seven new sesquiterpenes, laurocarpanes A–G (**1**–**7**), presenting this uncommon framework, were obtained from *Laurencia microcladia* (Figure 1).

## 2. Results and Discussion

### 2.1. Structural Elucidation of Macrocarpane-Type Sesquiterpenes

Fresh specimens of the alga *Laurencia microcladia* (2.0 kg) collected in El Cotillo, Fuerteventura (the Canary Islands, Spain) were extracted at room temperature using CH_2_Cl_2_/MeOH (1:1, *v*/*v*). The resultant extract was subjected to a multi-step chromatographic fractionation sequence to yield compounds **1**–**7** (Figure 1).

**Figure 2:**
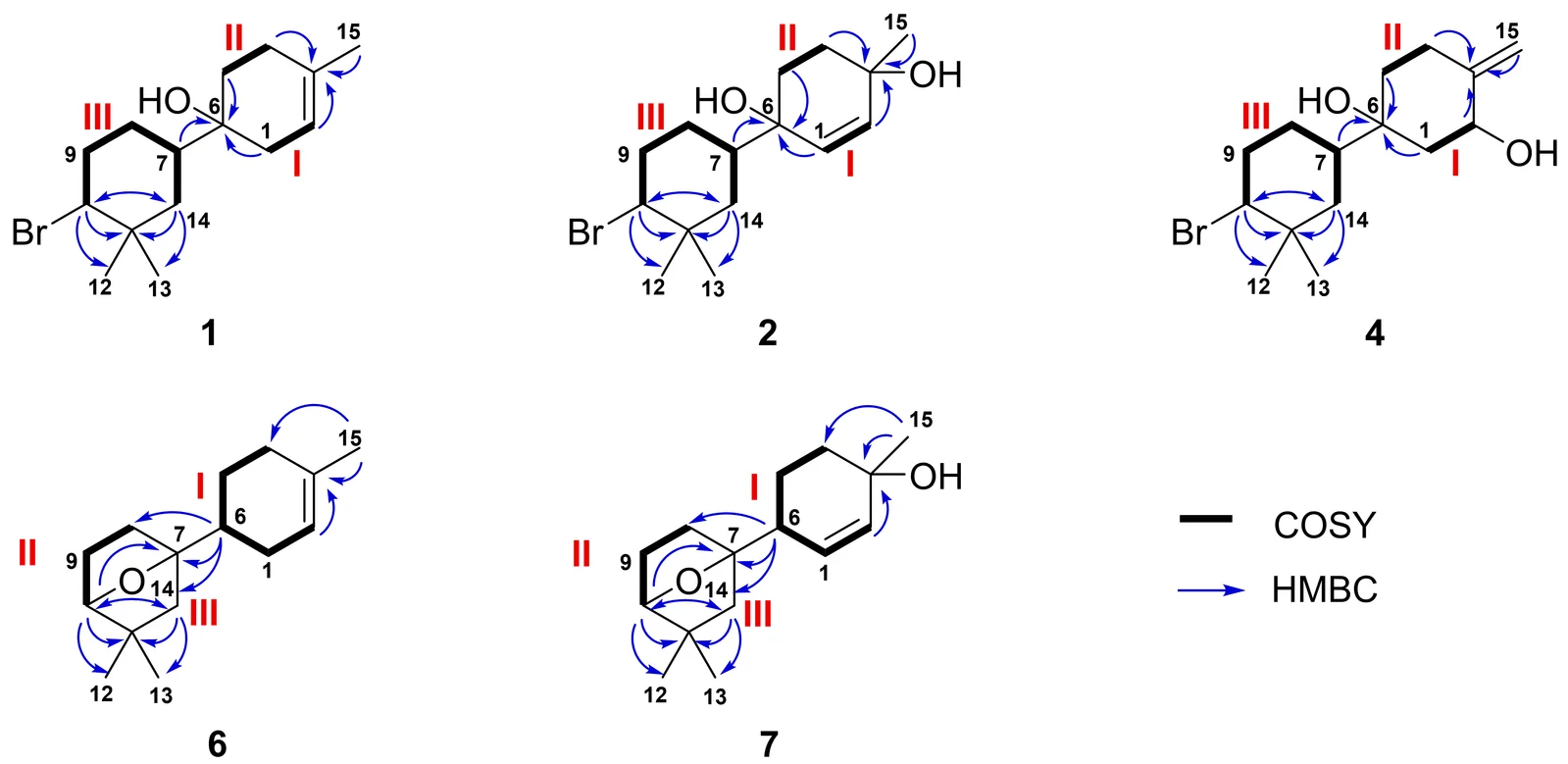
COSY and selected HMBC correlations of compounds **1**, **2**, **4**, **6**, and **7**.

Laurocarpane A (**1**) was isolated as a colorless oil. ESI-FTICR (ion peaks at *m*/*z* 301.1410 and 303.1390 ) revealed a molecular formula of C_15_H_25_BrO, indicating the presence of three degrees of unsaturation. The ^13^C NMR and HSQC-edited spectra established the presence of three methines (*δ*_C_ 118.1, 66.5, 41.3), six methylenes (*δ*_C_ 40.3, 35.2, 34.2, 31.0, 28.1, 26.9), three methyls (*δ*_C_ 31.9, 23.3, 20.4), and three non-protonated carbons (*δ*_C_ 134.0, 71.0, 36.6) (Table 1). The ^1^H NMR spectrum showed a broad vinylic signal at *δ*_H_ 5.27, a brominated methine at *δ*_H_ 3.94 (dd, *J* = 12.8, 4.4 Hz), a vinylic methyl at *δ*_H_ 1.68, and two singlet methyls at *δ*_H_ 1.07 and 1.04. These data, combined with COSY correlations, enabled the assignment of three independent spin systems: I [H_2_-1 (*δ*_H_ 2.14 and 1.89) ⟶ H-2 (*δ*_H_ 5.27)], II [H_2_-4 (*δ*_H_ 2.13 and 1.94) ⟶ H_2_-5 (*δ*_H_ 1.66 and 1.51)], and III [H-10 (*δ*_H_ 3.94)/H_2_-9 (*δ*_H_ 2.20 and 2.00)/H_2_-8 (*δ*_H_ 1.78 and 1.19)/H-7 (*δ*_H_ 1.61)/H_2_-14 (*δ*_H_ 1.82 and 1.21)] (Figure S1). Additionally, the HMBC correlations of H-2, H_2_-4, and H_3_-15 (δ_H_ 1.68) with the quaternary carbon C-3 (δ_C_ 134.0), as well as those of the methylenes H_2_-1 and H_2_-5 with the quaternary carbon C-6 (δ_C_ 71.0), connected the spin systems I and II in one of the molecule’s rings. Likewise, C-6 showed an HMBC correlation with H-7 (δ_H_ 1.61) that belonged to system III. Finally, the HMBC correlations from H-10, H_2_-14, and the gem-dimethyl groups H_3_-12 (δ_H_ 1.07) and H_3_-13 (δ_H_ 1.04) to C-11 (δ_C_ 36.6) completed the planar structure of **1**, establishing the existence of another cyclohexane ring (Figure 2).

**Figure 3:**
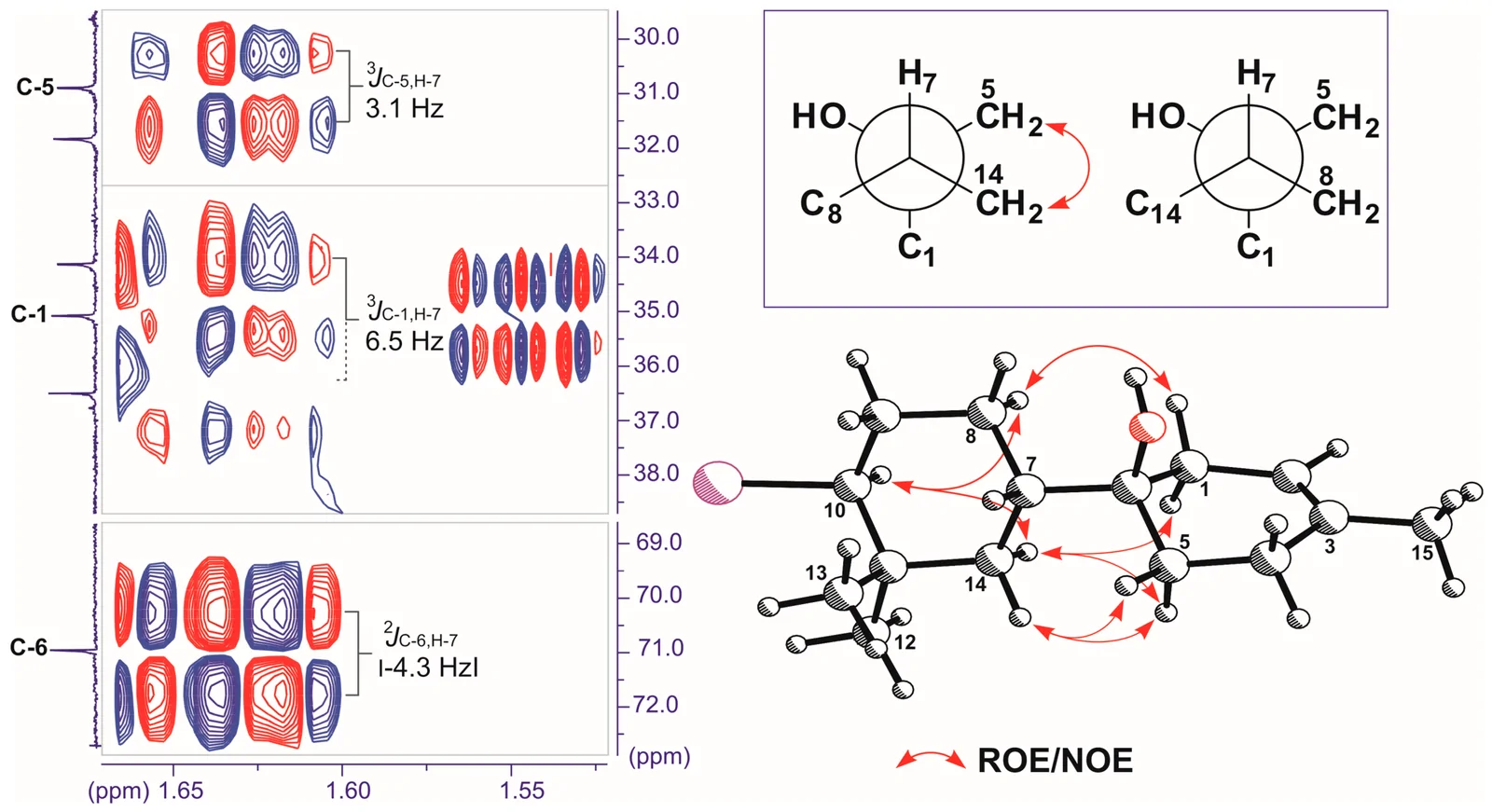
Determination of the relative stereochemistry of the C-7/C-6 fragment of laurocarpane A (**1**): *J*-HMBC experiment (600 MHz) in CDCl_3 _and the rotamers from coupling constant values and key ROE/NOE correlations.

The relative configuration of metabolite **1** was determined by comparison of TROESY data and the analysis of homo- and heteronuclear *J* couplings [14]. According to the values of the H-10 homonuclear coupling constants (*J*_H-H_ 12.8 and 4.4 Hz), its orientation must be axial, leaving the bromine atom in the equatorial position. In addition, a strong ROE enhancement observed between H-10 and one of the diastereotopic H-14 protons (*δ*_H_ 1.21), as well as its subsequent large coupling constant with H-7 (^3^*J*_H-14α,H-7_ = 12.6 Hz), was consistent with a *trans*-diaxial arrangement of H-14α/H-7 and an equatorial configuration of the bond that connects the two cyclohexane rings. Through a *J*-HMBC experiment, two rotamers around the C-6/C-7 bond were deduced (Figure 3). The large absolute value of 4.3 Hz obtained for the constant ^2^*J*_C-6,H-7_ indicates that the OH group on C-6 and H-7 are in a *gauche* relative position. Likewise, the values ^3^*J*_C-5,H-7_ = 3.1 Hz and ^3^*J*_C-1,H-7_ = 6.5 Hz agree with *gauche* (C-5/H-7) and *anti* (C-1/H-7) orientations. Finally, from the TROESY data and the 1D-NOE experiments for the H_2_-5 protons, it was possible to establish that they correlate exclusively with H_2_-14, which implies a 6R*, 7S* relative configuration.

Laurocarpane B (**2**) displayed a close structural relationship with **1**. The molecular formula was determined to be C_15_H_25_BrO_2_ on the basis of ESI-HRMS data. The IR spectrum showed bands attributable to hydroxyl (3402 cm^−1^) and alkene (1666 cm^−1^) type functional groups.

In fact, the comparison of the ^1^H and ^13^C NMR spectroscopic data (Table 1) shows a correspondence in the assignments between the positions C-7/C-11,C-14 (ring B), locating the difference between both compounds in ring A, more specifically in the presence of a disubstituted double bond (δ_H/C_ 5.77/136.8 and 5.67/131.3 in **2**) instead of a trisubstituted (δ_H/C_ 5.27/118.1 and 134.0 in **1**), and in the upfield shift of the H_3_-15 methyl protons from a vinylic methyl (δ_H_ 1.68 in **1**) to a methylcarbinol (δ_H_ 1.37 in **2**). The key differences between them were confirmed by the ^1^H-^1^H COSY correlations of I: H-1 (*δ*_H_ 5.77) ⟶ H-2 (*δ*_H_ 5.67) and II: H_2_-4 (*δ*_H_ 2.08/1.69) ⟶ H_2_-5 (*δ*_H_ 1.93/1.51); and the HMBC correlations of H_3_-15, H-2, H_2_-4/C-3 (*δ*_C_ 78.8) and H-1, H_2_-5, H-7 (*δ*_H_ 1.71)/C-6 (*δ*_C_ 70.9) (Figure 2). The double bond geometry was established as *Z* based on the value of the coupling constant ^3^*J*_H-1,H-2_ = 10.1 Hz. The relative configurations of the C-10, C-7 and C-6 centers matched that proposed for laurocarpane A (**1**), while that of C-3 was established as 3R* based on the observed ROE correlations of one of the methylene protons H_2_-4 at δ_H_ 1.69 with the more deshielded diastereotopic proton H-5 (δ_H_ 1.93) and methyl H_3_-15 (Table 1).

The (+)-HRESIMS spectrum of laurocarpane C (**3**) exhibited a [M + Na]^+^ ion at *m*/*z* 339.0940; 341.0922 (100:76), indicating that it is an isomer of **2**. Moreover, the NMR data of **3** were very similar to those of **2** (Table 1 and Table S1), with the exception of the chemical shifts at positions C-3 and C-15 (Δδ_C_ > 0.5 ppm). The analysis of the 2D NMR spectra (HSQC, HMBC, and COSY) for **3** established the same planar structure as that of **2**. Consequently, a different relative configuration at C-3 between **2** and **3** was proposed.

Laurocarpane D (**4**) shared the same molecular formula with compounds **2** and **3** according to analysis of ESI-FTICR (*m*/*z* 315.0954 and 317.0934 [M]^+^). A comparison of the ^13^C and ^1^H NMR spectra (Table 1 and Table S1) suggested the main differences among these isomers are found in ring A being the methylcarbinol in **2** and **3** replaced by an exocyclic double bond [*δ*_H_ 4.93 (s)/4.85 (s)], and an allylic oxymethine (*δ*_H_ 4.73, dd, *J* = 11.3, 4.6 Hz) in **4**. Analysis of the C-1/C-6 fragment through HSQC and COSY experiments revealed two ^1^H-^1^H spin systems [I: H_2_-1 (δ_H_ 2.10 and 1.43) → H-2 (δ_H_ 4.73); II: H_2_-4 (δ_H_ 2.40 and 2.26) → H_2_-5 (δ_H_ 1.64 and 1.47)]. Conversely, the HMBC study connected the H_2_-4 methylene and H-2 methine protons with the C-3 quaternary carbon signal (δ_C_ 146.4), which was also correlated with the exo olefinic protons (H_2_-15). Additionally, the connectivity of the H_2_-1, H_2_-5, and H-7 protons (δ_H_ 1.58) with the C-6 carbon (δ_C_ 74.9), which is adjacent to the hydroxyl group, was observed. This completed the structure of laurocarpane D (4) (Figure 2). As in the previous metabolites, the relative arrangement of the substituents at C-7 and C-10 on the dimethylcyclohexyl ring and the hydroxyl group at C-6 on ring A was determined to be *trans-diequatorial* and *axial*, respectively. Based on the coupling constants exhibited by the *axial* methine H-2 (*J* = 11.3, 4.6 Hz) with the two methylene protons H_2_-1, the spatial arrangement of the hydroxyl group on C-2 was assigned as *equatorial*. Therefore, the relative configuration at this center was proposed as 2S*.

Laurocarpane E (**5**) was isolated as a colorless, amorphous solid. Based on the pseudomolecular isotopic ions [M + Na]^+^ observed in the high-resolution mass spectrum (*m*/*z* 357.2040, 359.2204), its molecular formula was established as C_15_H_24_BrClO. The NMR spectra of laurocarpane E (**5**) were very similar to those of **4** (Table 1). The most significant differences were observed at the ^13^C NMR chemical shift of C-2 (δ_C_ 69.2 in **5** vs. δ_C_ 82.2 in **4**), which revealed the replacement of the hydroxy group of **4** by a chlorine atom in **5**. This finding is in agreement with the molecular formula and the relative intensities of isotope pseudomolecular peaks observed. The coupling constants observed for the axial proton H-2 (*J* = 10.7, 4.2 Hz) confirmed that the configuration at this stereocenter in **5** was identical to that of **4**.

**Figure 4:**
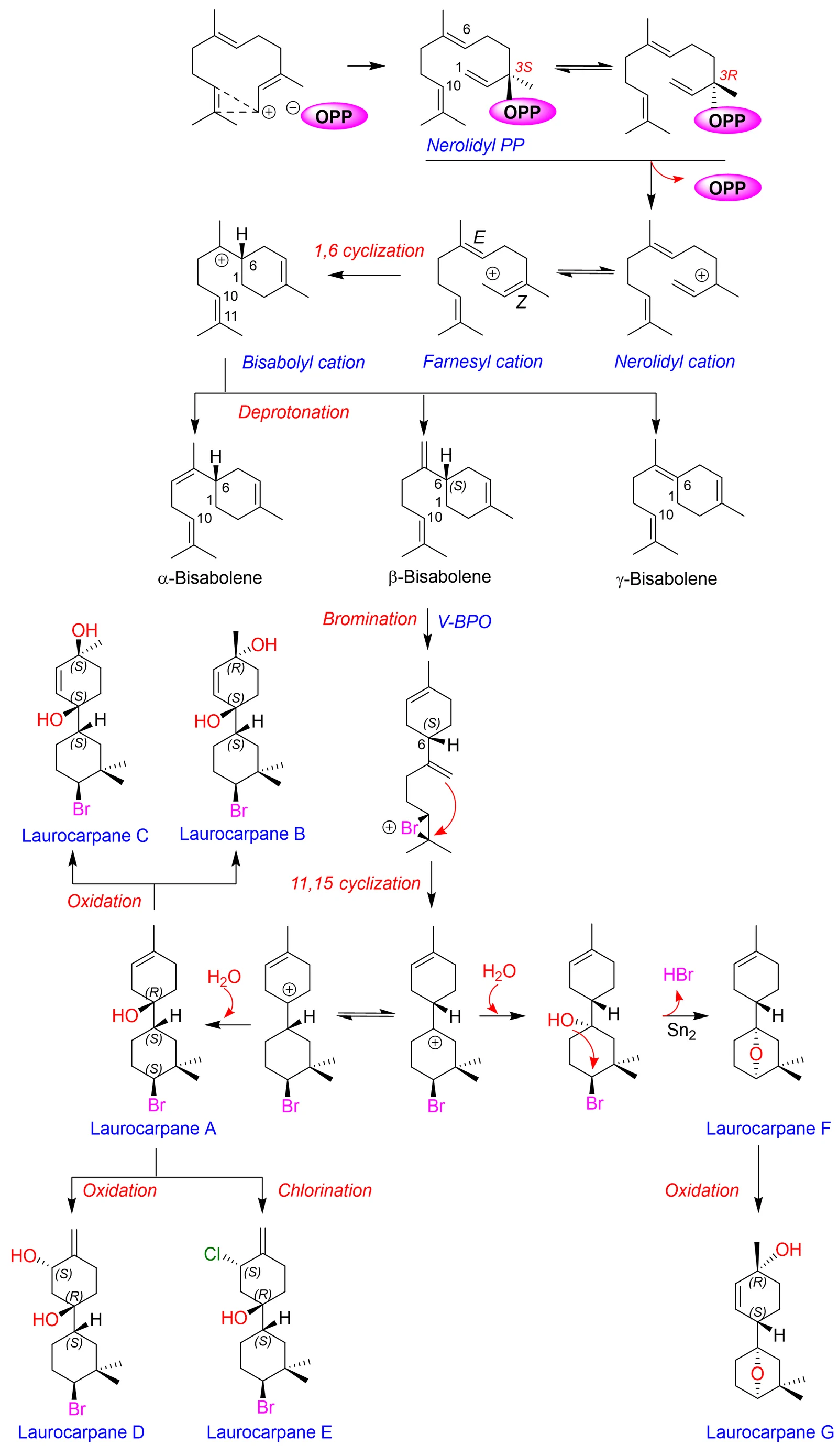
Biogenetic proposal for the formation of laurocarpanes A–G (**1**–**7**).

The molecular formula of laurocarpane F (**6**) was assigned by ESI-FTICR (*m*/*z* 259.1743 [M + K]^+^) as C_15_H_24_O, implying four degrees of unsaturation. The absence of characteristic absorptions of hydroxyl and/or carbonyl groups in the infrared spectrum indicated that the oxygen atom must correspond to an ether bridge. Through COSY, HSQC, and HMBC experiments, three spin systems could be established. The first system (I) begins with the olefinic methine H-2 (*δ*_H_ 5.39), which couples with the protons of the methylene H_2_-1 (*δ*_H_ 2.03 and 1.81). This sequence continues with the methine H-6 (δ_H_ 1.90) and the methylenes H_2_-5 (δ_H_ 1.82 and 1.29) and H_2_-4 (*δ*_H_ 2.04 and 1.92). The second system (II) comprises the methylenes H_2_-8 (δ_H_ 1.50 and 1.44) and H_2_-9 (δ_H_ 1.92 and 1.62), as well as the oxygenated methine H-10 (δ_H_ 3.84). The third spin system (III) consists solely of the isolated methylene H_2_-14 (δ_H_ 1.34 and 1.20) (Table 2; Figure 2). An analysis of the HMBC experiment revealed the ring A of the molecule through correlations observed between the methyl group at δ_H_ 1.65 (H_3_-15) and the olefinic carbons at δ_C_ 134.0 (C-3)/120.3 (C-2), as well as the terminal carbon of fragment I at δ_C_ 30.3 (C-4). Furthermore, methine H-10 connects to carbon at δ_C_ 90.5 (C-7) via an ether bridge. Additionally, correlations of this proton with carbons at δ_C_ 40.9 (C-11), 30.1 (C-12), 25.0 (C-13) and 48.6 (C-14) connect the gem-dimethyl system at C-11 to fragments II and III. HMBC correlations of H-6 with carbons at C-7, C-14, and C-8 (δ_C_ 31.2) confirm the final structure of this metabolite (Figure 2). The relative stereochemistry of this compound is proposed on the basis of biogenetic considerations. As mentioned in the background, and will be discussed in more detail subsequently, it is logical to hypothesize that the stereochemistry of laurocarpane F in ring A is derived from the chiral precursor (*S*)-*β*-bisabolene, while the carbocation on C-7 evolves by the addition of water and subsequent cyclization on C-10 on the opposite side to the bromine loss (Figure 4). Consequently, a relative configuration of 6S*, 7S*, 10S* is proposed.

Laurocarpane G (**7**) showed an ion [M]^+^ at *m*/*z* 236.1766 in its ESI-HRMS mass spectrum, indicating a molecular formula of C_15_H_24_O_2._ Given the similarity of the ^1^H and ^13^C NMR data for **7** to those of **6**, an analogous assignment strategy was used to elucidate its structure. The differences between the two are found in ring A. The double bond is *Z*-disubstituted rather than trisubstituted, with the vinylic methines H-1 and H-2 at δ_H_ 6.07 and 5.65 (^3^*J*_H-1,H-2_ = 10.2 Hz), respectively. The methine H-6 is now allylic, resulting in its displacement to lower fields (δ_H_ 2.42). Finally, the olefinic methyl H_3_-15 becomes a methylcarbinol (δ_H_ 1.35). The HMBC correlations between the protons H_3_-15/H-1/H_2_-4 (δ_H_ 2.17/1.43) and the hydroxylated carbon at δ_C_ 78.9 (C-3) complete the structure of this particular region of the molecule (Figure 2). In order to formulate a proposal for relative stereochemistry at the C-6, C-7, and C-10 centers, the same biogenetic postulates as in **6** are used. However, the configuration at C-3 is proposed as R* on the basis of the correlation observed in the TROESY experiment between the methine H-6 and the most shielded diastereotopic proton of methylene H_2_-4 (δ_H_ 1.43). This places the hydroxyl group on the opposite face of the molecule, as in compound **2**.

### 2.2. Plausible Biosynthetic Pathway

The biogenetic origin of the macrocarpane skeleton from the 2*Z*,6*E*-nerolidyl cation via a key monocyclic intermediate, (*S*)-*β*-bisabolene, has been described in detail by Cool [2] and experimentally supported by Köllner and colleagues [15]. Based on these data, we propose a biogenetic model for the formation of the metabolites isolated from *L. microcladia* (Figure 4).

The hypothesis posits that the formation of a bromonium ion on the olefin between C-10 and C-11, via vanadium-dependent bromoperoxidase (V-BPO), could induce the formation of the macrocarpane cation at C-7. The reaction of water with the carbocation could result in the subsequent formation of an ether bridge with C-10 on the opposite side from the bromine loss, leading to the formation of laurocarpanes F and G (**6**–**7**). On the other hand, hydride migration from C-6 to C-7 could generate the cation at C-6, which upon the subsequent addition of water would result in laurocarpane A. The action of peroxidases or chloroperoxidases would result in the formation of laurocarpanes B–E (**2**–**5**).

### 2.3. Computational Analysis

**Figure 5:**
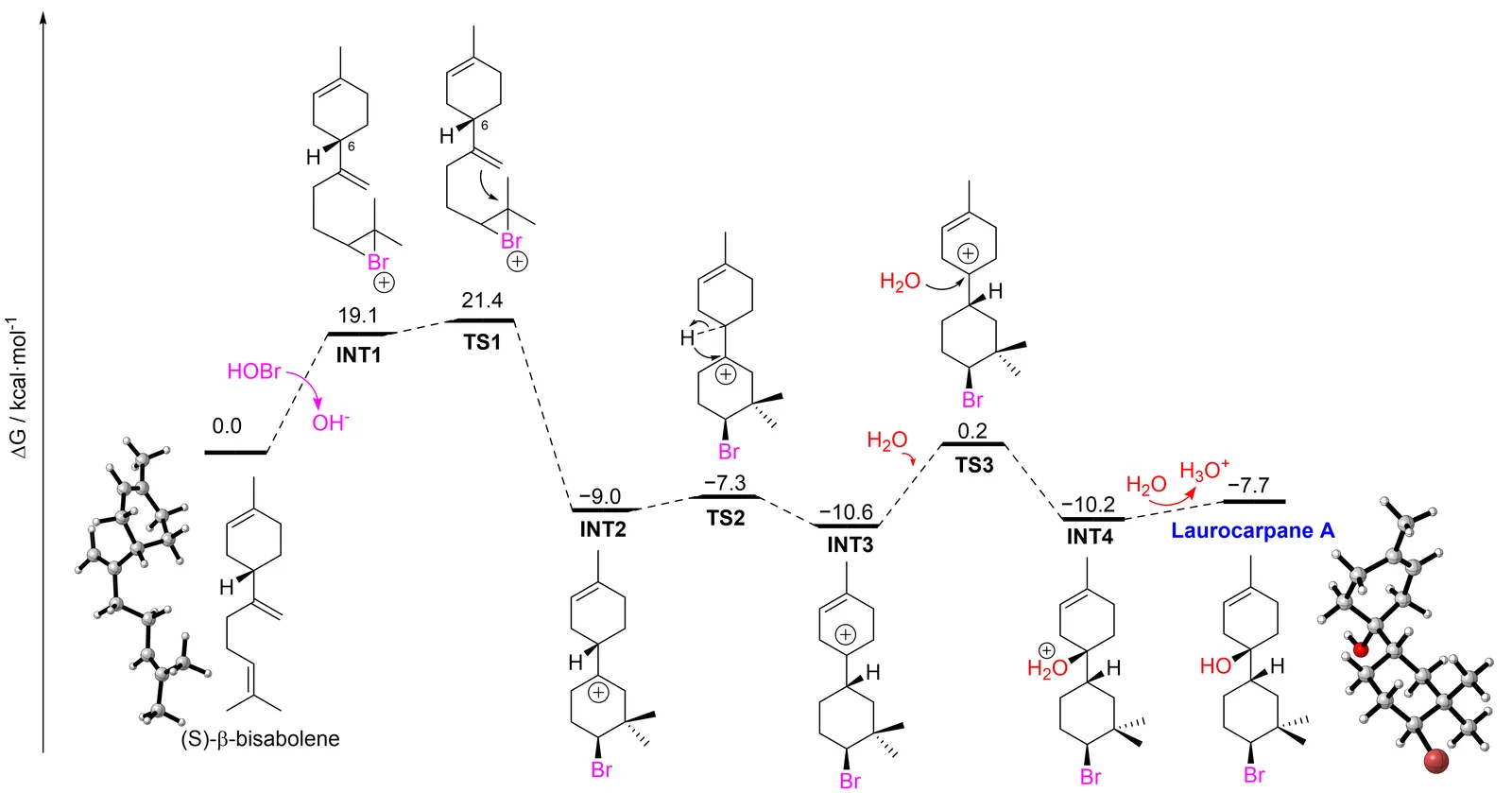
Computed Gibbs free energy profile for the proposed biogenetic formation of **laurocarpane A** from (*S*)-*β*-bisabolene. Relative free energies (ΔG) are given with respect to the initial substrate in kcal/mol. All data were computed at the SMD(water)-PBE0-D3BJ/def2TZVPP//PBE0-D3BJ/def2SVP.

To assess the plausibility of the proposed biogenetic formation of laurocarpanes A–G (**1**–**7**), Density Functional Theory (DFT) calculations were performed on the mechanism shown in Figure 4 (see ESI for computational details). Firstly, we focused on the formation of laurocarpane A (**1**), hypothesized to be the key biosynthetic precursor to laurocarpanes B–E (**2**–**5**). The computed reaction profile (Figure 5) begins with electrophilic bromination of (*S*)-*β*-bisabolene to generate the bromonium cation **INT1** in a markedly endergonic step (ΔG = 19.1 kcal/mol). In line with previous studies on related red algae [16], we propose that this energetically demanding activation is mediated by a vanadium-dependent bromoperoxidase, which catalyzes the oxidation of bromide by hydrogen peroxide to generate an electrophilic brominating species, commonly formulated as HOBr or an equivalent Br^+^-donating species, capable of initiating substrate bromination [16,17].

Once **INT1** is formed, the subsequent cyclization leading to the characteristic bicyclic laurocarpane scaffold proceeds through a remarkably low-energy transition state (**TS1**). This step requires an activation barrier of only 2.3 kcal/mol relative to the bromonium intermediate and remains accessible at physiological temperature from the initial bisabolene substrate with an overall barrier of 21.4 kcal/mol. **TS1** affords the bicyclic cation **INT2** in a strongly exergonic process relative to the starting bisabolene (ΔG = −9.0 kcal/mol). These results indicate that, although bromination is the most energetically demanding step, the subsequent ring-closing event is highly facile and provides a strong driving force for the rapid assembly of the bicyclic skeleton.

Next, we suggest that **INT2** is in equilibrium with **INT3** through a 1,2-hydride shift. Consistent with this hypothesis, we located the corresponding Meerwein–Wagner rearrangement transition state, **TS2**, which proceeds with a very low activation barrier in both directions: 1.7 kcal/mol from **INT2** to **INT3** and 3.3 kcal/mol for the reverse process.

The subsequent addition of water to **INT3** occurs through **TS3** (ΔG^‡^ = 10.8 kcal/mol), yielding **INT4** in a nearly isoenergetic step relative to **INT3**, although this transformation remains strongly exergonic with respect to the initial bisabolene (ΔG = −10.2 kcal/mol). Finally, deprotonation of **INT4** yields laurocarpane A in an overall exergonic process (ΔG = −7.7 kcal/mol). The accessible computed activation barriers, together with the overall exergonic character of the sequence, support the plausibility of the proposed biogenetic formation of laurocarpane A and are consistent with its proposed role as a common precursor to laurocarpanes B–E.

**Figure 6:**
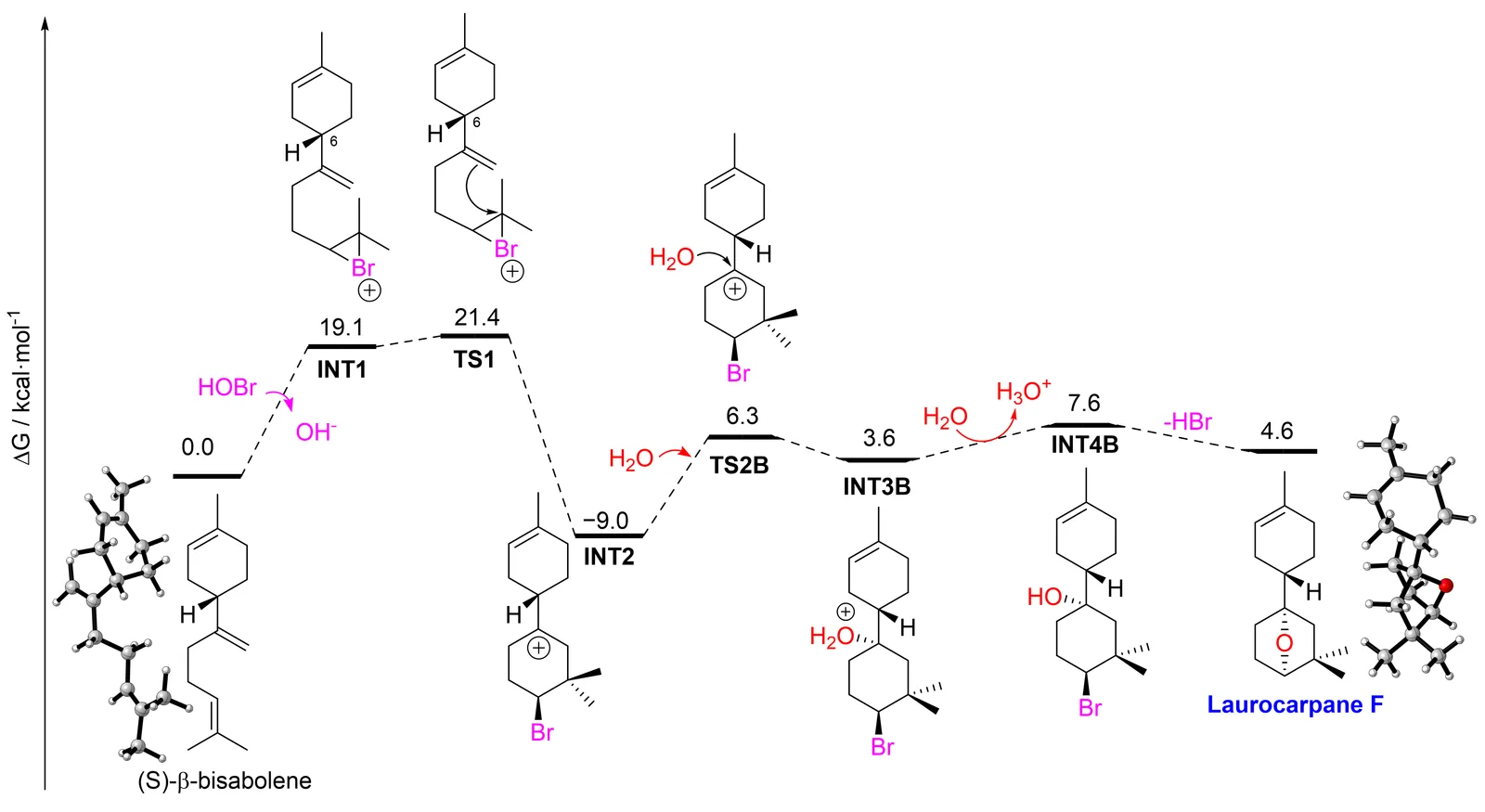
Computed Gibbs free energy profile for the proposed biogenetic formation of laurocarpane F from *β*-bisabolene. Relative free energies (ΔG) are given with respect to the initial substrate in kcal/mol. All data were computed at the SMD(water)-PBE0-D3BJ/def2TZVPP//PBE0-D3BJ/def2SVP.

We then investigated by DFT the mechanism for the formation of laurocarpane F (Figure 6). Laurocarpane F is hypothesized to be the biosynthetic precursor of laurocarpane G, as both compounds share the same cyclic ether motif.

The computed mechanism shown in Figure 6 indicates that the pathway leading to laurocarpane F is identical to that proposed for laurocarpane A up to the formation of the bicyclic carbocation **INT2**. From this point onward, however, the reaction diverges. Instead of undergoing a 1,2-hydride shift, **INT2** is trapped by water through the transition state **TS2B**, with an associated activation free energy of 15.3 kcal/mol, to give **INT3B**. This intermediate lies slightly higher in energy than the initial bisabolene substrate (ΔG = 3.6 kcal/mol). From **INT3B**, subsequent deprotonation and intramolecular cyclic ether formation accompanied by HBr elimination afford laurocarpane F in a process that is overall slightly endergonic relative to the initial bisabolene (ΔG = 4.6 kcal/mol). Despite this modest thermodynamic penalty, the relatively low computed barrier for water trapping of **INT2** indicates that this competing pathway is kinetically accessible under biosynthetic conditions. Therefore, the DFT results support the feasibility of laurocarpane F formation and its plausible role as a precursor to laurocarpane G.

### 2.4. Preliminary Bioactivity Assay

The apoptosis-inducing activity of the major compound, laurocarpane A (**1**), was investigated against the human hepatocellular carcinoma cell line HepG2 (HB-8065). Interestingly, laurocarpane A induced apoptosis in more than 98% of HepG2 cells at a concentration of 50 μM. Unfortunately, the limited availability and stability of the isolated compounds prevented the completion of a comprehensive bioactivity study. Nevertheless, these preliminary results suggest that laurocarpane A has potential as an apoptosis-inducing agent.

## 3. Materials and Methods

### 3.1. General Experimental Procedures

The optical rotations were obtained using a Perkin–Elmer 241 polarimeter (Waltham, MA, USA) equipped with a sodium lamp. UV spectra were acquired on a Jasco V-560 spectrophotometer (Easton, MD, USA). The infrared spectra were collected on a Bruker IFS55 spectrometer (Ettlingen, Germany). NMR spectra were recorded on a Bruker AVANCE 600 MHz instrument (Bruker, Karlsruhe, Germany) equipped with a 5 mm TCI (Triple Resonance Inverse) detection cryo-probe. Chemical shifts were referenced to the CDCl_3_ signals at 300 K (δ_H_ 7.26 ppm, δ_C_ 77.0 ppm). The 2D NMR experiments (COSY, HSQC, HMBC, and ROESY) were performed using standard pulse sequences. ^3^*J*_H,H_ values were measured from 1D ^1^H NMR. The *J*-HMBC pulse sequence was used to measure long-range heteronuclear coupling constants. A *J*-scale factor of 52 was used, and the experiment was optimized for long-range couplings of 2 Hz. Mass spectra were recorded on a LCT Premier XE Micromass spectrometer (Waters, Milford, CT, USA) using electrospray ionization. HPLC separations were carried out with a preparative silica column (10 μm, 19 × 150 mm) and Waters system (Waters, Milford, CT, USA) equipped with a Binary HPLC Pump 1525 and Photodiode Array Detector 2996. TLC (thin-layer chromatography) (Merck, Darmstadt, Germany) plates were visualized by spraying with phosphomolybdic acid reagent (10% in EtOH) and heating.

### 3.2. Biological Material

Specimens of *Laurencia microcladia* Kützing were collected by hand at a depth of 2 m at El Cotillo (Fuerteventura, the Canary Islands) (GPS coordinates 28°42′41.7″ N, 14°00′27.2″ W). A voucher specimen was deposited at the Department of Biología Vegetal, Botánica, University of La Laguna, Tenerife (TFC Phyc 14446).

### 3.3. Extraction and Isolation

Fresh alga (2.0 kg) was extracted with CHCl_3_:MeOH (1:1, *v*/*v*, 3×) at room temperature and the solvent removed in vacuo to give a dark-green viscous oil. The resulting extract (14.1 g) was subjected to gel filtration using Sephadex LH-20 (70 × 600 mm) and eluted with CHCl_3_:MeOH (1:1) to obtain five fractions. Fraction LM-4 (4.78 g) was rechromatographed on a medium-pressure normal-phase Lobar LiChroprep Si 60 column (25 × 310 mm) using a step gradient elution of increasing polarity (n-hexane to EtOAc) to yield five fractions. Final purifications of fractions LM-41 (eluted with 10% EtOAc in n-Hex) and LM-42 (30% EtOAc in n-Hex) were achieved on a μ-Porasil HPLC column (10 μm, 19 × 150 mm) using n-hexane:EtOAc (9:1 and 7:3, respectively), yielding compounds **1** (5.0 mg), **2** (1.4 mg), **3** (1.0 mg), **4** (2.8 mg), **5** (2.0 mg), **6** (1.8 mg), and **7** (2.5 mg).

*Laurocarpane A* (**1**): colorless oil; [α]^25^_D_ +5 (*c* 0.57, CHCl_3_); UV (MeOH) λ_max_ (log ε) 203 (3.11) nm; IR (CHCl_3_) ν_max_ 3416, 2950, 2871, 1706, 1665, 1453, 1369, 1265, 1217, 963 cm^−1^; ^1^H and ^13^C NMR data (CDCl_3_), see Table 1; ESI-FTICR *m*/*z* 301.1410, 303.1390 [M + H]^+^ (100:82) (calcd for C_15_H_26_^79^BrO, 301.1167; C_15_H_26_^81^BrO, 303.1147).

*Laurocarpane B* (**2**): colorless oil; [α]^25^_D_ + 1 (*c* 0.02, CHCl_3_); UV (MeOH) λ_max_ (log ε) 204 (3.53) nm; IR (CHCl_3_) ν_max_ 3402, 2930, 2859, 1714, 1666, 1456, 1372, 1264, 1021, 964, 865 cm^−1^; ^1^H and ^13^C NMR data (CDCl_3_), see Table 1; ESI-HRMS *m*/*z* 339.0911, 341.0928 [M + Na]^+^ (100:80) (calcd for C_15_H_25_^79^BrO_2_Na, 339.0936; C_15_H_25_^81^BrO_2_Na, 341.0915).

*Laurocarpane C* (**3**): colorless oil; [α]^25^_D_ −4 (*c* 0.20, CHCl_3_); UV (MeOH) λ_max_ (log ε) 204 (3.14) nm; IR (CHCl_3_) ν_max_ 3400, 2930, 2868, 1729, 1665, 1455, 1367, 1253, 1021, 984, 763 cm^−1^; ^1^H and ^13^C NMR data (CDCl_3_), see Table S1; ESI-HRMS *m*/*z* 339.0940, 341.0922 [M + Na]^+^ (100:76) (calcd for C_15_H_25_^79^BrO_2_Na, 339.0936; C_15_H_25_^81^BrO_2_Na, 341.0915).

*Laurocarpane D* (**4**): colorless oil; [α]^25^_D_ + 4 (*c* 0.08, CHCl_3_); UV (MeOH) λ_max_ (log ε) 203 (3.67) nm; IR (CHCl_3_) ν_max_ 3393, 2926, 2855, 1650, 1456, 1368, 1260, 1080, 1023, 963, 898 cm^−1^; ^1^H and ^13^C NMR data (CDCl_3_), see Table 1; ESI-FTICR *m*/*z* 315.0954, 317.0934 [M]^+^ (100:97) (calcd for C_15_H_24_^79^BrO_2_, 315.0960; C_15_H_24_^81^BrO_2_, 317.0939).

*Laurocarpane E* (**5**): white amorphous solid; ^1^H and ^13^C NMR data (CDCl_3_), see Table S1; ESI-HRMS *m*/*z* 357.2040, 359.2204 [M + Na]^+^ (100:96) (calcd for C_15_H_24_^79^Br^35^ClONa, 357.0597; C_15_H_24_^81^Br^35^ClONa, 359.0576). Complementary spectroscopic data are not available due to the rapid degradation of the sample.

*Laurocarpane F* (**6**): white amorphous solid; [α]^25^_D_ + 16 (*c* 0.03, CHCl_3_); UV (MeOH) λ_max_ (log ε) 202 (3.38) nm; IR (CHCl_3_) ν_max_ 2955, 2928, 2867, 1729, 1450, 1367, 1240, 1253, 1161, 988, 751 cm^−1^; ^1^H and ^13^C NMR data (CDCl_3_), see Table 2; ESI-FTICR *m*/*z* 259.1743 [M + K]^+^ (calcd for C_15_H_24_OK, 259.1464).

*Laurocarpane G* (**7**): white amorphous solid; [α]^25^_D_ + 16 (*c* 0.03, CHCl_3_); UV (MeOH) λ_max_ (log ε) 202 (3.14) nm; IR (CHCl_3_) ν_max_ 3400, 2955, 2928, 2867, 1729, 1450, 1368, 1253, 1195, 1161, 988, 751 cm^−1^; ^1^H and ^13^C NMR data (CDCl_3_), see Table 2; ESI-HRMS *m*/*z* 236.1766, [M]^+^ (calcd for C_15_H_24_O_2_, 236.1776).

### 3.4. Density Functional Theory (DFT) Calculations

All geometry optimizations were performed without imposing symmetry or geometrical constraints using the Gaussian 16 quantum chemistry package, Revision B.01 [18]. The PBE0 [19] hybrid density functional was employed in combination with Grimme’s D3 empirical dispersion correction with Becke–Johnson damping [20,21] together with the def2-SVP [22] basis set for all atoms. Frequency calculations were carried out at the same level of theory to characterize the stationary points, confirming that all minima exhibited no imaginary frequencies, whereas each transition state showed exactly one imaginary frequency. The connectivity of the transition states with their corresponding minima was verified by intrinsic reaction coordinate (IRC) [23] calculations. Single-point energy refinements were subsequently carried out on the optimized geometries using the larger def2-TZVPP [22] basis set. Solvent effects were included in these single-point calculations through the SMD implicit solvation model [24], using water as the solvent. Accordingly, the overall computational protocol is denoted as SMD(water)-PBE0-D3(BJ)/def2-TZVPP//PBE0-D3(BJ)/def2-SVP. Figures of the 3D molecular geometries were generated using CYLview, version 1.0 [25].

### 3.5. Apoptosis-Inducing Activity

The human hepatocellular carcinoma cell line HepG2 (ATCC HB-8065) was obtained from the American Type Culture Collection (ATCC) and maintained in DMEM supplemented with 10% FBS, penicillin/streptomycin (100 U/mL and 100 μg/mL, respectively), and glutamine (2 mM) at 37 °C in a humidified atmosphere containing 5% CO_2_. Laurocarpane A (**1**) was dissolved in DMSO and added to the culture medium at the appropriate concentration. The final DMSO concentration did not exceed 0.25% (*v*/*v*) in either treated or control cultures.

Apoptosis was assessed by phosphatidylserine externalization using annexin V-FITC, while cell membrane integrity was evaluated by propidium iodide (PI) staining. Briefly, HepG2 cells were cultured in 96-well plates and treated with laurocarpane A (**1**) or vehicle. Following treatment, adherent and non-adherent cells were collected and analyzed by capillary flow cytometry using a Guava EasyCyte Plus cytometer (Millipore/Merck). At least 2000 cells were analyzed per sample using InCyte software version 2.7 (Guava/Millipore/Merck, CA, USA). Annexin V-FITC and PI staining were performed according to the manufacturers’ instructions (ImmunoTools and Miltenyi Biotec, respectively).

## 4. Conclusions

In conclusion, this study reports the isolation and structural elucidation of seven previously undescribed sesquiterpenes, named laurocarpanes A–G (**1**–**7**), from fresh specimens of the red marine alga *Laurencia microcladia* collected in Fuerteventura, the Canary Islands. These metabolites possess a highly uncommon macrocarpane bicyclic framework, expanding the limited family of macrocarpane metabolites currently documented from marine and terrestrial sources. Although sesquiterpenes represent one of the most structurally diverse classes of secondary metabolites in Rhodophyta, their carbon skeletons are not evenly distributed in nature. The high prevalence of the chamigrane, cuparane, and bisabolane families contrasts sharply with the macrocarpane framework, which has been reported only sporadically.

From chemical and chemotaxonomic perspectives, the identification of these macrocarpane-type sesquiterpenes within *L. microcladia* is of particular interest. Because terpenoid profiles serve as valuable markers for assessing relationships among algal taxa, the identification of these compounds within the investigated species provides deeper insights into its distinctive, highly specialized metabolic capabilities and clarifies the distribution of this uncommon structural class within marine red algae.

Furthermore, a plausible biosynthetic model was proposed linking the assembly of the macrocarpane skeleton directly to an (*S*)-*β*-bisabolene monocyclic precursor. The pathway is initiated via vanadium-dependent bromoperoxidase (V-BPO) mediation, generating a transient bromonium ion intermediate. To evaluate the chemical and energetic validity of this hypothesis, Density Functional Theory (DFT) calculations were performed. The computational results confirmed that, while the initial enzyme-assisted bromination step demands significant energetic input, the subsequent intramolecular ring-closing events to form the bicyclic framework proceed via highly accessible, low-energy transition states.

Preliminary bioactivity evaluation showed that laurocarpane A (**1**) induced apoptosis in more than 98% of HepG2 cells at 50 μM, suggesting promising biological potential.

Ultimately, the successful characterization of laurocarpanes A–G (**1–7**) substantially enriches our current understanding of the structural and biosynthetic diversity of marine natural products and serves as a crucial baseline for future investigations into the ecological roles and potential biological activities of these rare macrocarpane frameworks.

## Figures and Tables

**Table 1:** ^1^H NMR (600 MHz) and ^13^C NMR (150 MHz) data of **1**, **2**, and **4** in CDCl_3_.

Position	Laurocarpane A (1)		Laurocarpane B (2)		Laurocarpane D (4)	
	δ_C_	δ_H_, mult. (*J* in Hz)	δ_C_	δ_H_, mult. (*J* in Hz)	δ_C_	δ_H_, mult. (*J* in Hz)
1	35.2	α 2.14, dd (3.2, 15.5)	136.8	5.77, d (10.1)	40.0	α 2.10, dd (4.6, 12.1)
		β 1.89, *br* d (15.5)				β 1.42, dd (11.3, 12.1)
2	118.1	5.27, *br* s	131.3	5.67, d (10.1)	82.7	4.73, dd (4.6, 11.3)
3	134.0		78.8		146.4	
4	26.9	2.13, m	28.2	β 2.08, m	29.5	α 2.40, ddd (4.1, 13.2, 13.7)
		1.94, m		α 1.69, m		β 2.26, ddd (3.6, 3.8, 13.7)
5	31.0	α 1.66, ddd (2.9, 5.9, 13.0)	28.0	α 1.93, ddd (3.7, 12.8, 13.7)	35.6	α 1.64, ddd (3.6, 4.1, 13.4)
		β 1.51, ddd (6.1, 11.5, 13.0)		β 1.51, ddd (3.7, 4.8, 13.7)		β 1.47, ddd (3.8, 13.2, 13.4)
6	71.0		70.9		74.9	
7	41.3	1.61, dddd (3.2, 3.4, 12.6, 12.6)	42.2	1.71, dddd (2.8, 3.2, 13.1, 13.3)	43.2	1.58, dddd (2.9, 3.1, 12.3, 13.2)
8	28.1	α 1.78, dddd (3.4, 3.9, 4.2, 13.1)	27.8	β 1.88, dddd (2.8, 3.1, 4.0, 13.2)	28.2	β 1.84, dddd (3.1, 3.7, 3.9, 13.0)
		β 1.19, dddd (3.5, 12.6, 13.1, 13.1)		α 1.20, dddd (4.4, 13.0, 13.2, 13.3)		α 1.16, dddd (3.7, 13.0, 13.2, 13.5)
9	34.2	α 2.20, dddd (3.5, 3.9, 4.4, 13.4)	34.1	α 2.20, dddd (3.1, 4.1, 4.4, 13.4)	34.0	α 2.21, dddd (3.7, 3.7, 4.0, 13.3)
		β 2.00, dddd (4.2, 12.8, 13.1, 13.4)		β 1.99, dddd (4.0, 12.7, 13.0, 13.4)		β 1.99, dddd (3.9, 12.7, 13.3, 13.5)
10	66.5	3.94, dd (4.4, 12.8)	66.1	3.91, dd (4.1, 12.7)	65.7	3.90, dd (4.0, 12.7)
11	36.6		36.7		36.6	
12	31.9	1.07, s (3H)	31.9	1.06, s (3H)	31.9	1.07, s (3H)
13	20.4	1.04, s (3H)	20.6	1.04, s (3H)	20.5	1.03, s (3H)
14	40.3	β 1.82, ddd (2.8, 3.2, 13.4)	40.9	β 1.64, ddd (2.9, 3.2, 13.3)	40.4	β 1.75, ddd (2.7, 2.9, 13.1)
		α 1.21, ddd (2.8, 12.6, 13.4)		α 1.15, dd (13.1, 13.3)		α 1.17, *br* dd (12.3, 13.1)
15	23.3	1.68, s (3H)	23.4	1.37, s (3H)	105.7	4.93, s 4.85, s

**Table 2:** ^1^H NMR (600 MHz) and ^13^C NMR (150 MHz) data of **6** and **7** in CDCl_3_.

Position	Laurocarpane F (6)		Laurocarpane G (7)	
	δ_C_	δ_H_, mult. (*J* in Hz)	δ_C_	δ_H_, mult. (*J* in Hz)
1	27.3	2.03, m 1.81, m	134.9	6.07, d (10.2)
2	120.3	5.39, *br* s	129.4	5.65, d (10.2)
3	134.0		78.9	
4	30.3	2.04, m 1.92, m	31.6	2.17, m 1.43, m
5	24.6	1.82, m 1.29, m	21.5	1.63, m 1.51, m
6	39.3	1.90, m	41.6	2.42, m
7	90.5		89.6	
8	31.2	1.50, ddd (3.5, 3.5, 11.9) 1.44, m	33.4	1.52, m 1.45, m
9	25.9	1.92, m 1.62, m	25.9	1.94, ddd (4.0, 9.0, 12.7) 1.67, m
10	84.9	3.84, *br* d (5.2)	84.9	3.86, *br* d (5.3)
11	40.9		41.0	
12	30.1	1.03, s (3H)	29.4	1.03, s (3H)
13	25.0	1.03, s (3H)	24.9	1.02, s (3H)
14	48.6	1.34, dd (2.3, 11.6) 1.20, *br* d (11.6)	46.6	1.32, *br* d (11.8) 1.16, *br* d (11.8)
15	23.6	1.65, s (3H)	24.8	1.35, s (3H)

## Data Availability

The original contributions presented in this study are included in the article/supplementary material. Further inquiries can be directed to the corresponding authors.

## Supplementary Materials

The following supporting information can be downloaded at: https://www.mdpi.com/article/10.3390/md24090316/s1. Table S1: ^1^H NMR (600 MHz) and ^13^C NMR (150 MHz) data of 3 and 5 in CDCl_3_; Figure S1: Key 2D NMR spectra for laurocarpane A (**1**): (A) HSQC and key COSY/HMBC correlations, and (B) COSY spectrum; Figures S2–S9: NMR spectra (^1^H, ^13^C, ^1^H-^1^H COSY, HSQC, HMBC, *J*-HMBC, TROESY) and ESI-FTICR mass spectrum of laurocarpane A (1); Figures S10–S16: NMR spectra (^1^H, ^13^C, ^1^H-^1^H COSY, HSQC, HMBC, TROESY) and ESI- HRMS mass spectrum of laurocarpane B (2); Figures S17–S22: NMR spectra (^1^H, ^13^C, ^1^H-^1^H COSY, HSQC, HMBC) and ESI- HRMS mass spectrum of laurocarpane C (3); Figures S23–S29: NMR spectra (^1^H, ^13^C, ^1^H-^1^H COSY, HSQC, HMBC, TROESY) and ESI-FTICR mass spectrum of laurocarpane D (4); Figures S30–S34: NMR spectra (^1^H, ^13^C, ^1^H-^1^H COSY, HSQC, HMBC) and ESI- HRMS mass spectrum of laurocarpane E (5); Figures S35–S40: NMR spectra (^1^H, ^13^C, ^1^H-^1^H COSY, HSQC, HMBC) and ESI-FTICR mass spectrum of laurocarpane F (6); Figures S41–S47: NMR spectra (^1^H, ^13^C, ^1^H-^1^H COSY, HSQC, HMBC) and ESI-FTICR mass spectrum of laurocarpane G (7); computational details; Cartesian coordinates and energies in au of DFT-optimized structures.
