# Supplementary material for: Macrocarpane-Type Sesquiterpenes from Laurencia microcladia
Source: Mar Drugs. 2026 Sep 9;24(9):316. doi: 10.3390/md24090316 (PMC13608081; doi:10.3390/md24090316)
Supplement: Supplementary file 1 [file marinedrugs-24-00316-s001.zip › marinedrugs-4503392-supplementary.pdf]

Supporting Information

Macrocarpane–Type Sesquiterpenes from *Laurencia microcladia*

Adrián Gutiérrez-Cepeda[1,2], Jorge J. Cabrera-Trujillo[1,3], José J. Fernández[1,3,4,*], Antonio Hernández-Daranas[5] , Manuel Norte[1,3] and María L. Souto[1,3,4,*]

[1] Instituto Universitario de Bio-Orgánica Antonio González (IUBO AG), Universidad de La Laguna (ULL), Avenida Astrofísico Francisco Sánchez 2, 38206 La Laguna, Spain; aguticep@gmail.com (A.G.-C.); jcabrert@ull.edu.es (J.J.C.-T.); mnorte@ull.es (M.N.)
[2] Department of Chemistry, Chemistry Institute, Sciences Faculty, Autonomous University of Santo Domingo, University City, 1355 Santo Domingo, Dominican Republic
[3] Departamento de Química Orgánica, Universidad de La Laguna (ULL), Avenida Astrofísico Francisco Sánchez 3, 38206 La Laguna, Spain
[4] Biotecnología Marina, IUBO-ULL, Unidad Asociada al IPNA-CSIC, 38206 La Laguna, Tenerife, Spain
[5] Instituto de Productos Naturales y Agrobiología (IPNA), Consejo Superior de Investigaciones Científicas (CSIC), Avenida Astrofísico Francisco Sánchez 3, 38206 La Laguna, Tenerife, Spain; adaranas@ipna.csic.es (A. H.-D.)

Corresponding Author

* E-mail: jjfercas@ull.edu.es
* E-mail: msouto@ull.edu.es

Table of contents:

Table S1. ^{1}H NMR (600 MHz) and ^{13}C NMR (150 MHz) data of **3** and **5** in $CDCl_3$. S4

Figure S1. Key 2D NMR spectra for laurocarpane A (**1**): (A) HSQC and key COSY/HMBC correlations, and (B) COSY spectrum. S5

Figure S2. ^{1}H NMR spectrum (600 MHz, $CDCl_3$) of laurocarpane A (**1**). S6

Figure S3. ^{13}C NMR spectrum (150 MHz, $CDCl_3$) of compound **1**. S7

Figure S4. ^{1}H-^{1}H COSY NMR spectrum (600 MHz, $CDCl_3$) of compound **1**. S8

Figure S5. HSQC NMR spectrum (600 MHz, $CDCl_3$) of compound **1**. S9

Figure S5. HMBC NMR spectrum (600 MHz, $CDCl_3$) of compound **1**. S10

Figure S7 *J*-HMBC NMR spectrum (600 MHz, $CDCl_3$) of compound **1**. S11

Figure S8. TROESY NMR spectrum (600 MHz, $CDCl_3$) of compound **1**. S12

Figure S9. ESI-FTICR mass spectrum for compound **1**. S13

Figure S10. ^{1}H NMR spectrum (600 MHz, $CDCl_3$) of laurocarpane B (**2**). S14

Figure S11. ^{13}C NMR spectrum (150 MHz, $CDCl_3$) of compound **2**. S15

Figure S12. ^{1}H-^{1}H COSY NMR spectrum (600 MHz, $CDCl_3$) of compound **2**. S16

Figure S13. HSQC NMR spectrum (600 MHz, $CDCl_3$) of compound **2**. S17

Figure S14. HMBC NMR spectrum (600 MHz, $CDCl_3$) of compound **2**. S18

Figure S15. TROESY NMR spectrum (600 MHz, $CDCl_3$) of compound **2**. S19

Figure S16. ESI-HRMS mass spectrum for compound **2**. S20

Figure S17. ^{1}H NMR spectrum (600 MHz, $CDCl_3$) of laurocarpane C (**3**). S21

Figure S18. ^{13}C NMR spectrum (150 MHz, $CDCl_3$) of compound **3**. S22

Figure S19. ^{1}H-^{1}H COSY NMR spectrum (600 MHz, $CDCl_3$) of compound **3**. S23

Figure S20. HSQC NMR spectrum (600 MHz, $CDCl_3$) of compound **3**. S24

Figure S21. HMBC NMR spectrum (600 MHz, $CDCl_3$) of compound **3**. S25

Figure S22. ESI-HRMS mass spectrum for compound **3**. S26

Figure S23. ^{1}H NMR spectrum (600 MHz, $CDCl_3$) of laurocarpane D (**4**). S27

Figure S24. ^{13}C NMR spectrum (150 MHz, $CDCl_3$) of compound **4**. S28

Figure S25. ^{1}H-^{1}H COSY NMR spectrum (600 MHz, $CDCl_3$) of compound **4**. S29

Figure S26. HSQC NMR spectrum (600 MHz, $CDCl_3$) of compound **4**. S30

Figure S27. HMBC NMR spectrum (600 MHz, $CDCl_3$) of compound **4**. S31

Figure S28. TROESY NMR spectrum (600 MHz, $CDCl_3$) of compound **4**. S32

Figure S29. ESI-FTICR mass spectrum for compound **4**. S33

Figure S30. ^{1}H NMR spectrum (600 MHz, $CDCl_3$) of laurocarpane E (**5**). S34

Figure S31. ^{1}H-^{1}H COSY NMR spectrum (600 MHz, $CDCl_3$) of compound **5**. S35

Figure S32. HSQC NMR spectrum (600 MHz, $CDCl_3$) of compound **5**. S36

Figure S33. HMBC NMR spectrum (600 MHz, $CDCl_3$) of compound **5**. S37

Figure S34. ESI-HRMS mass spectrum for compound **5**. S38

Figure S35. ^{1}H NMR spectrum (600 MHz, $CDCl_3$) of laurocarpane F (**6**). S39
Figure S36. ^{13}C NMR spectrum (150 MHz, $CDCl_3$) of compound **6**. S40
Figure S37. ^{1}H-^{1}H COSY NMR spectrum (600 MHz, $CDCl_3$) of compound **6**. S41
Figure S38. HSQC NMR spectrum (600 MHz, $CDCl_3$) of compound **6**. S42
Figure S39. HMBC NMR spectrum (600 MHz, $CDCl_3$) of compound **6**. S43
Figure S40. ESI-FTICR mass spectrum for compound **6**. S44
Figure S41. ^{1}H NMR spectrum (600 MHz, $CDCl_3$) of laurocarpane G (**7**). S45
Figure S42. ^{13}C NMR spectrum (150 MHz, $CDCl_3$) of compound **7**. S46
Figure S43. ^{1}H-^{1}H COSY NMR spectrum (600 MHz, $CDCl_3$) of compound **7**. S47
Figure S44. HSQC NMR spectrum (600 MHz, $CDCl_3$) of compound **7**. S48
Figure S45. HMBC NMR spectrum (600 MHz, $CDCl_3$) of compound **7**. S49
Figure S46. TROESY NMR spectrum (600 MHz, $CDCl_3$) of compound **7**. S50
Figure S47. ESI-FTICR mass spectrum for compound **7**. S51
Computational Details S52
Cartesian coordinates and energies in au of DFT-optimized structures S54

Table S1.^{1}H NMR (600 MHz) and ^{13}C NMR (150 MHz) data of **3** and **5** in $CDCl_3$.

Position	**Laurocarpane C (3)**		**Laurocarpane E (5)**	
	δ_C	δ_H, mult. (*J* in Hz)	δ_C	δ_H, mult. (*J* in Hz)
1	135.7	5.76, dd (1.4, 10.1)	45.0	α 2.07, dd (4.2, 12.6)<br>β 1.33, dd (10.7, 12.6)
2	133.0	5.61, dd (1.5, 10.1)	69.2	4.73, dd (4.2, 10.7)
3	67.2		150.5	
4	28. 0	β 1.93, ddd (3.5, 13.3, 13.7)<br>α 1.59, dddd (1.5, 3.2, 4.6, 13.7)	29.3	α 2.42, ddd (4.5, 13.5, 13.5)<br>β 2.31, ddd (2.4, 4.6, 13.5)
5	33.6	α 1.86, ddd (3.2, 13.3, 13.5)<br>β 1.77, dddd (1.4, 3.5, 4.6, 13.5)	35.7	α 1.68, ddd (2.4, 4.5, 13.4)<br>β 1.42, ddd (4.6, 13.4, 13.5)
6	70.8		74.3	
7	42.4	1.73, m	43.6	1.56, m
8	27.9	β 1.93, dddd (3.1, 3.4, 4.1, 13.1)<br>α 1.20, dddd (4.1, 12.7, 13.1, 13.2)	28.3	β 1.84, dddd (3.5, 3.5, 13.0, 13.0)<br>α 1.18, dddd (4.1, 13.0, 13.0, 13.0)
9	34.1	α 2.24, dddd (3.3, 4.1, 4.2, 13.3)<br>β 2.03, dddd (4.1, 12.7, 13.1, 13.3)	34.1	α 2.24, dddd (4.0, 4.0, 4.3, 13.4)<br>β 2.01, dddd (4.1, 12.6, 13.1, 13.4)
10	66.0	3.94, dd (4.2, 12.7)	65.7	3.92, dd (4.3, 12.6)
11	37.0		36.7	
12	31.9	1.09, s (3H)	32.1	1.09, s (3H)
13	20.5	1.08, s (3H)	20.5	1.05, s (3H)
14	41.1	β 1.69, ddd (3.2, 3.2, 13.0)<br>α 1.19, *br* dd (13.0, 13.0)	40.6	β 1.79, ddd (2.3, 2.8, 13.2)<br>α 1.18, *br* dd (13.2, 13.2)
15	29.8	1.35, s (3H)	103.7	4.94, s<br>4.82, s:

Figure S1. Key 2D NMR spectra for laurocarpane A (**1**): (A) HSQC and key COSY/HMBC correlations, and (B) COSY spectrum.

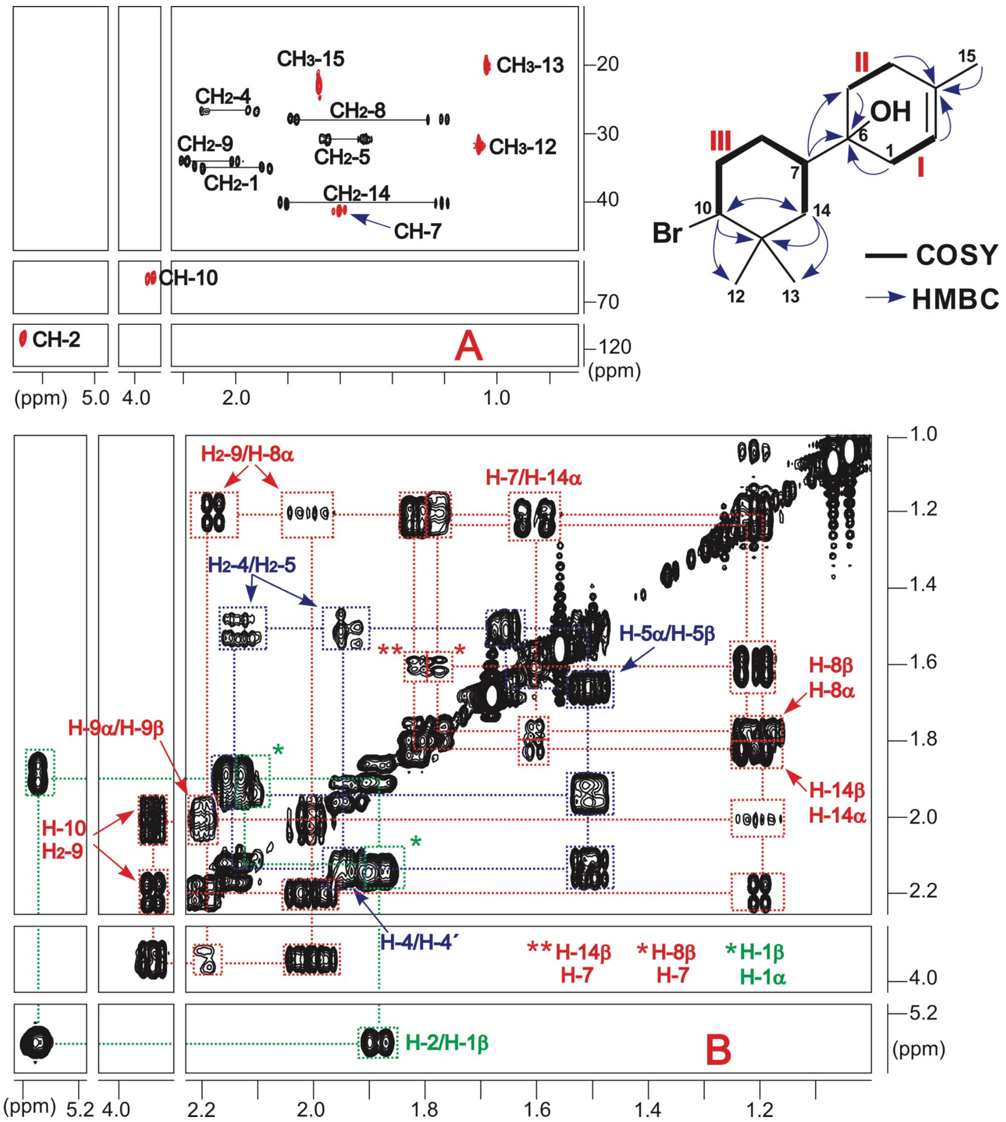

Figure S2. ^{1}H NMR spectrum (600 MHz, $CDCl_3$) of laurocarpane A (**1**).

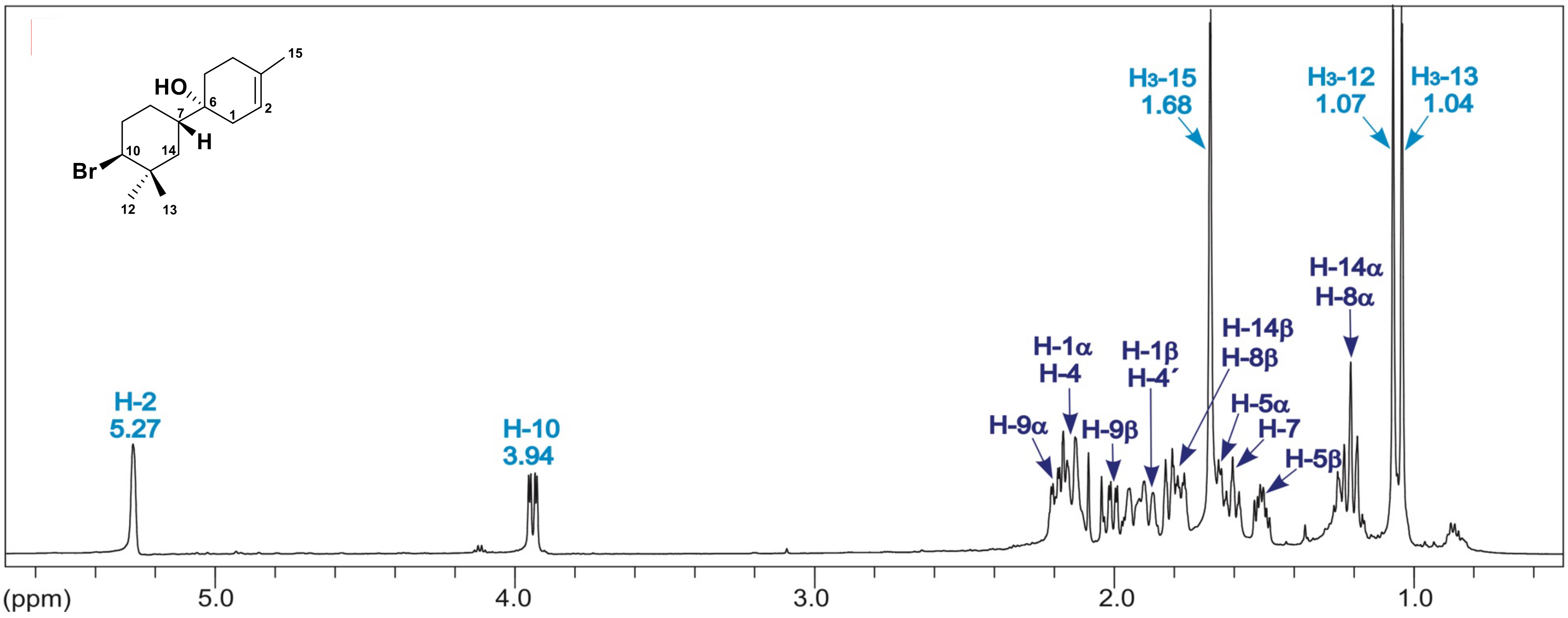

Figure S3. ^{13}C NMR spectrum (150 MHz, $CDCl_3$) of compound **1**.

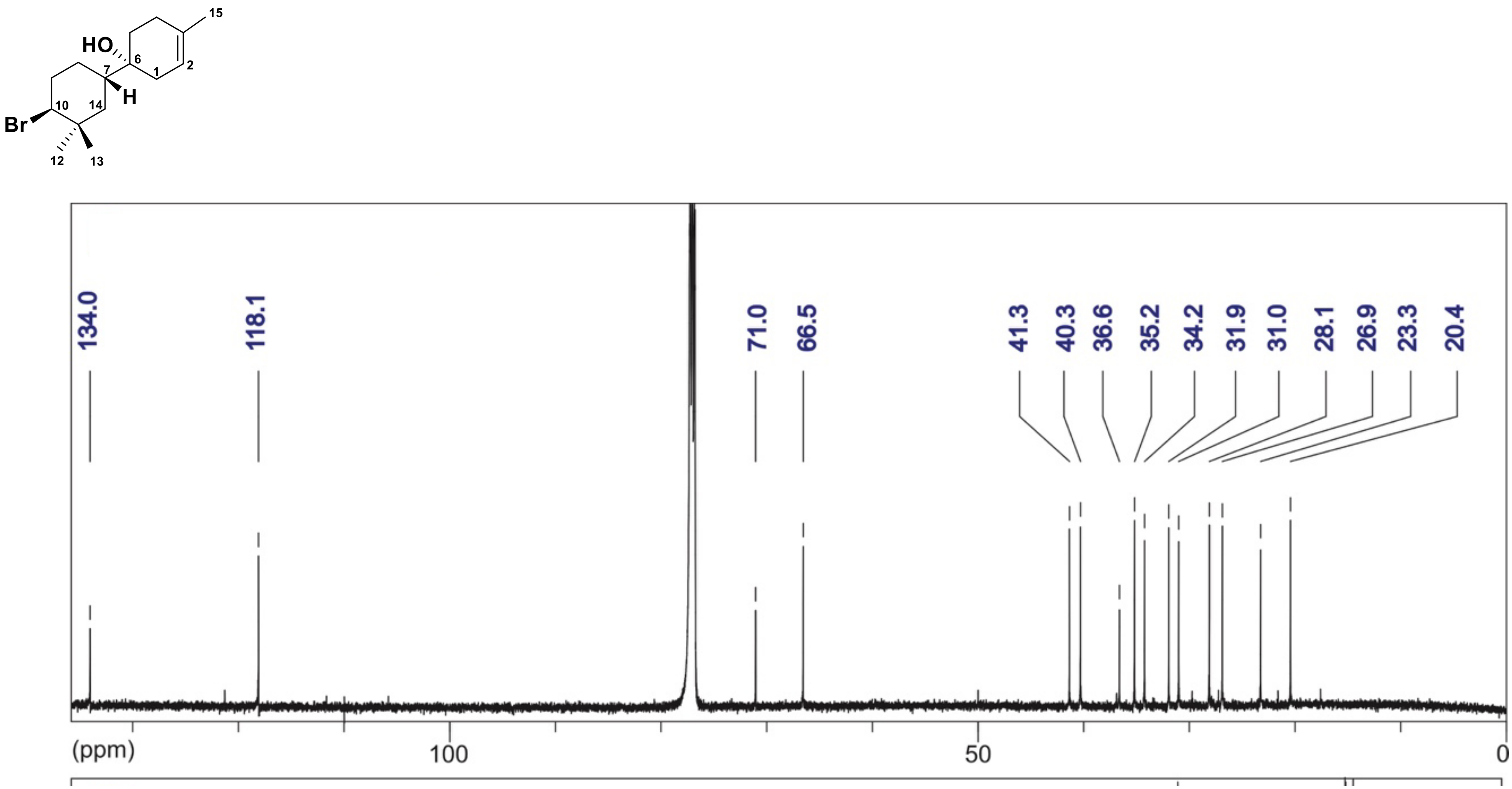

Figure S4. ^{1}H-^{1}H COSY NMR spectrum (600 MHz, $CDCl_3$) of compound **1**.

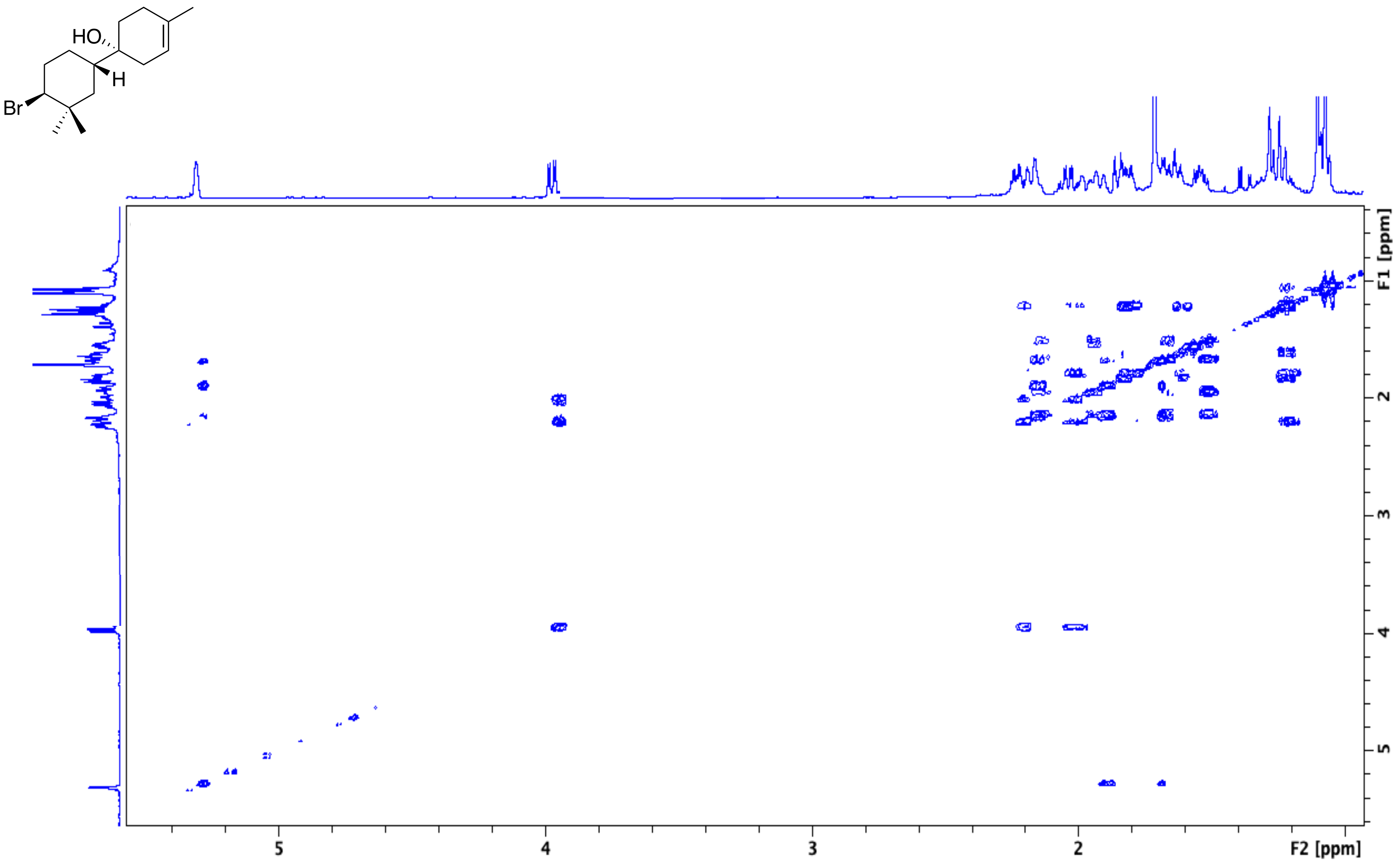

Figure S5. HSQC NMR spectrum (600 MHz, $CDCl_3$) of compound **1**.

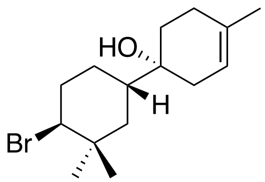

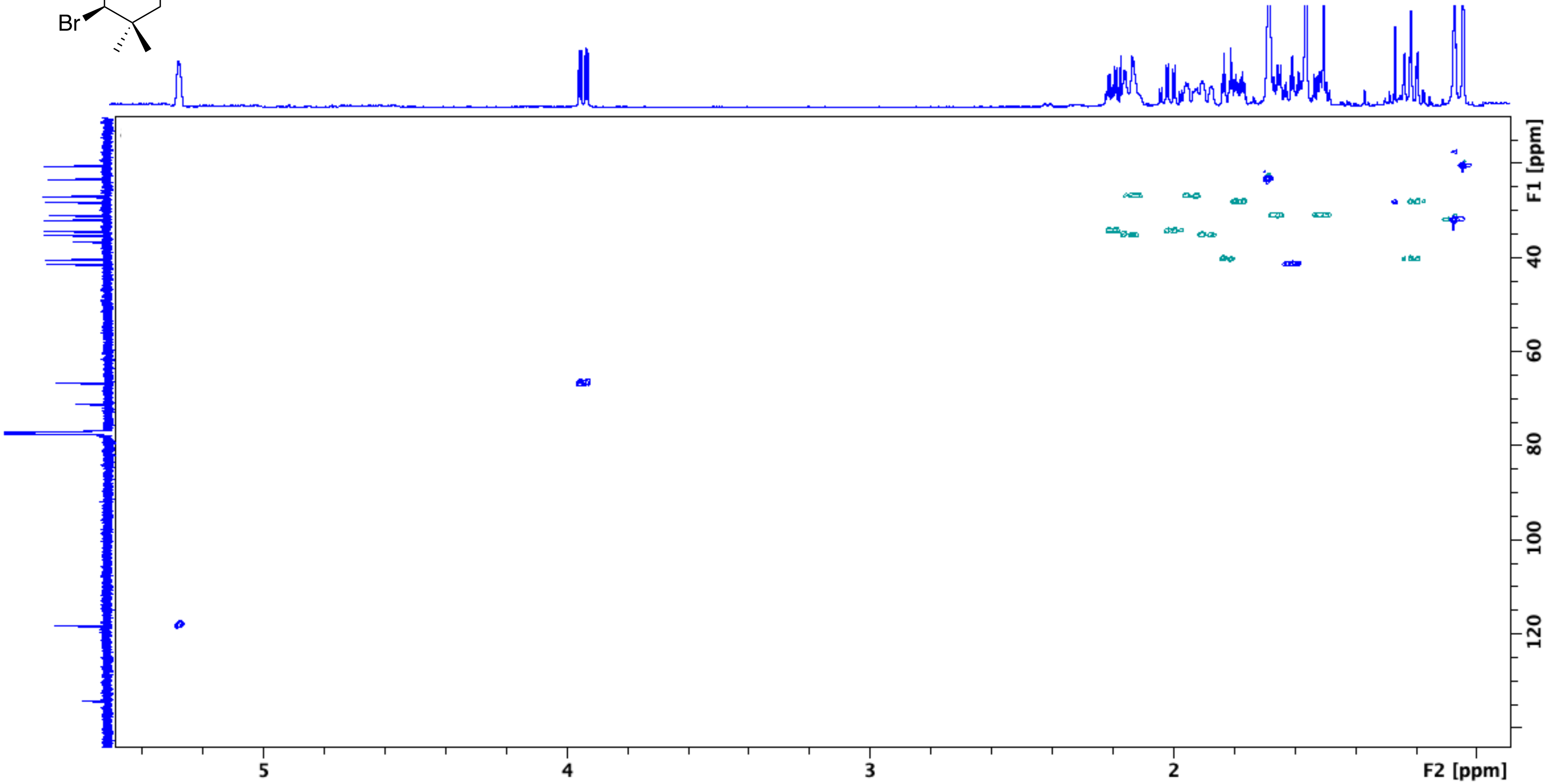

Figure S6. HMBC NMR spectrum (600 MHz, $CDCl_3$) of compound **1**.

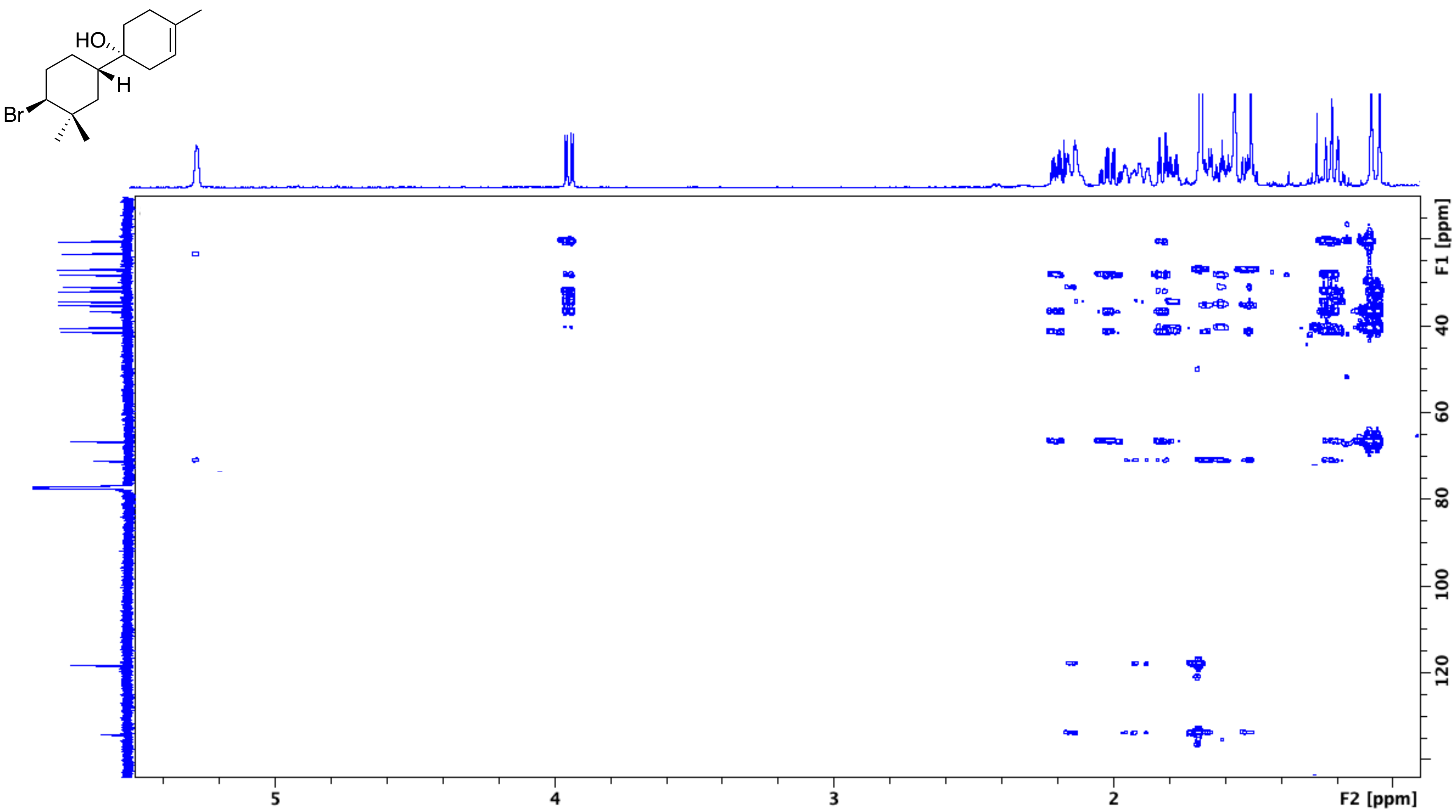

Figure S7. *J*-HMBC NMR spectrum (600 MHz, $CDCl_3$) of compound **1**.

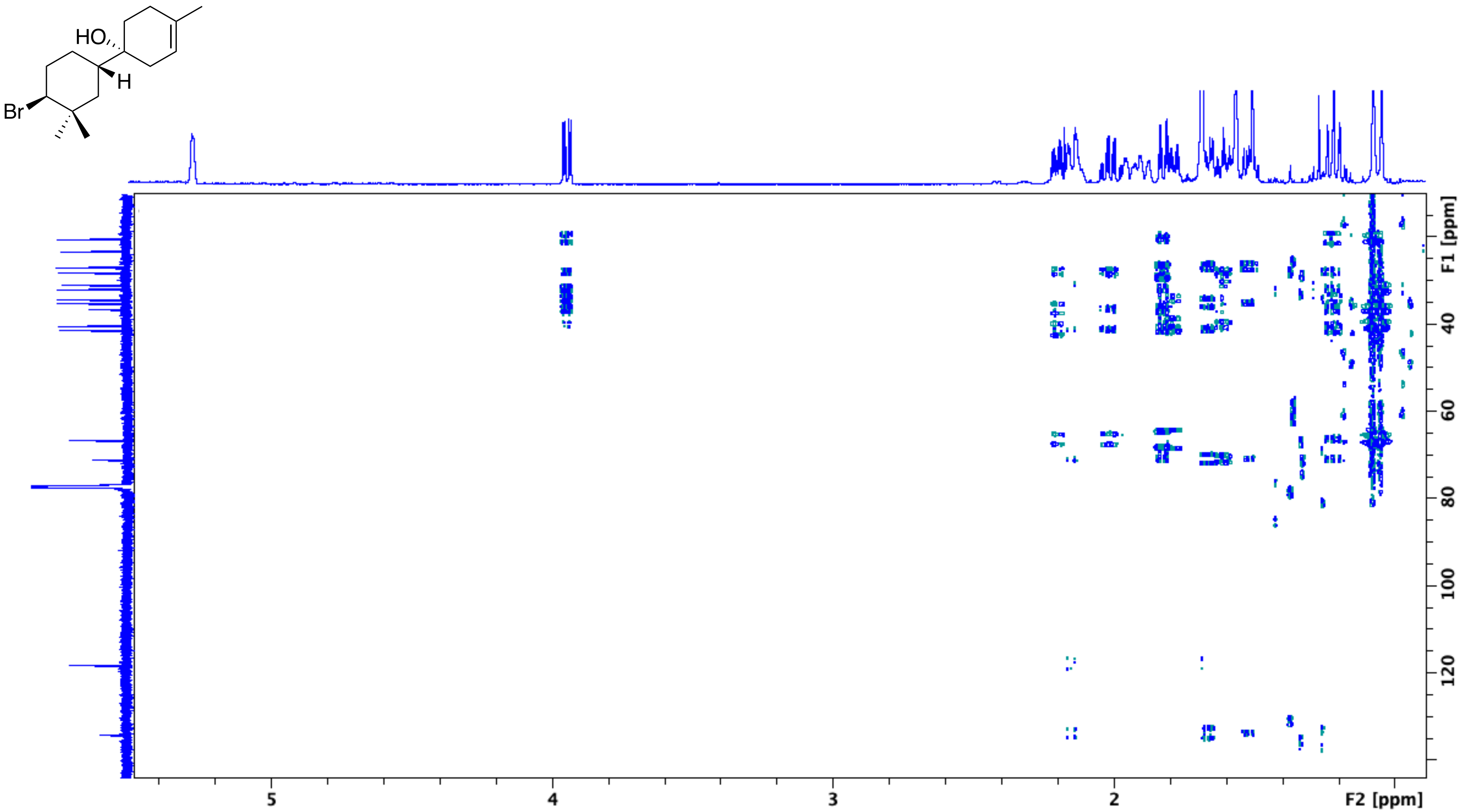

Figure S8. TROESY NMR spectrum (600 MHz, $CDCl_3$) of compound **1**.

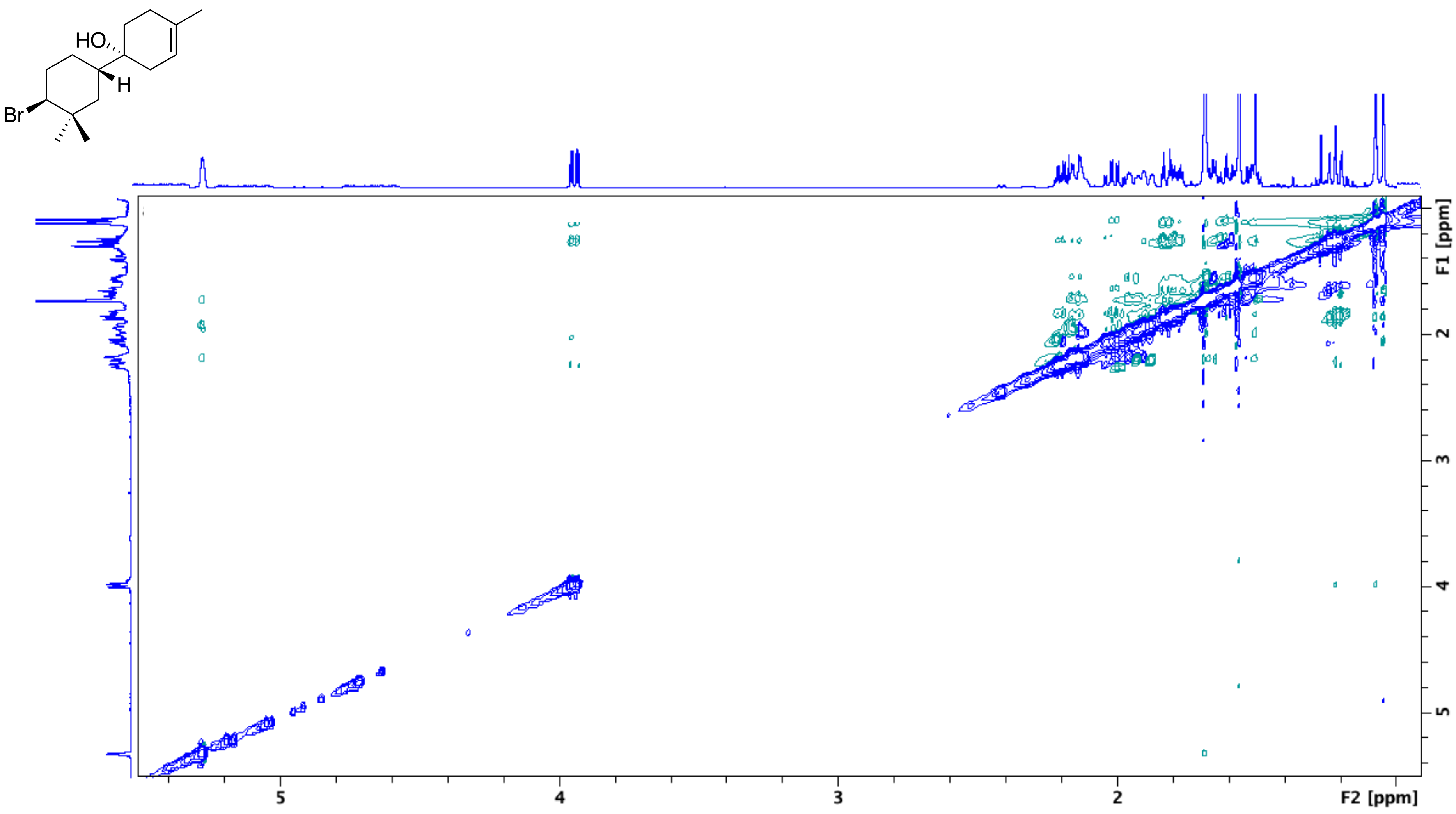

Figure S9. ESI-FTICR mass spectrum for compound **1**.

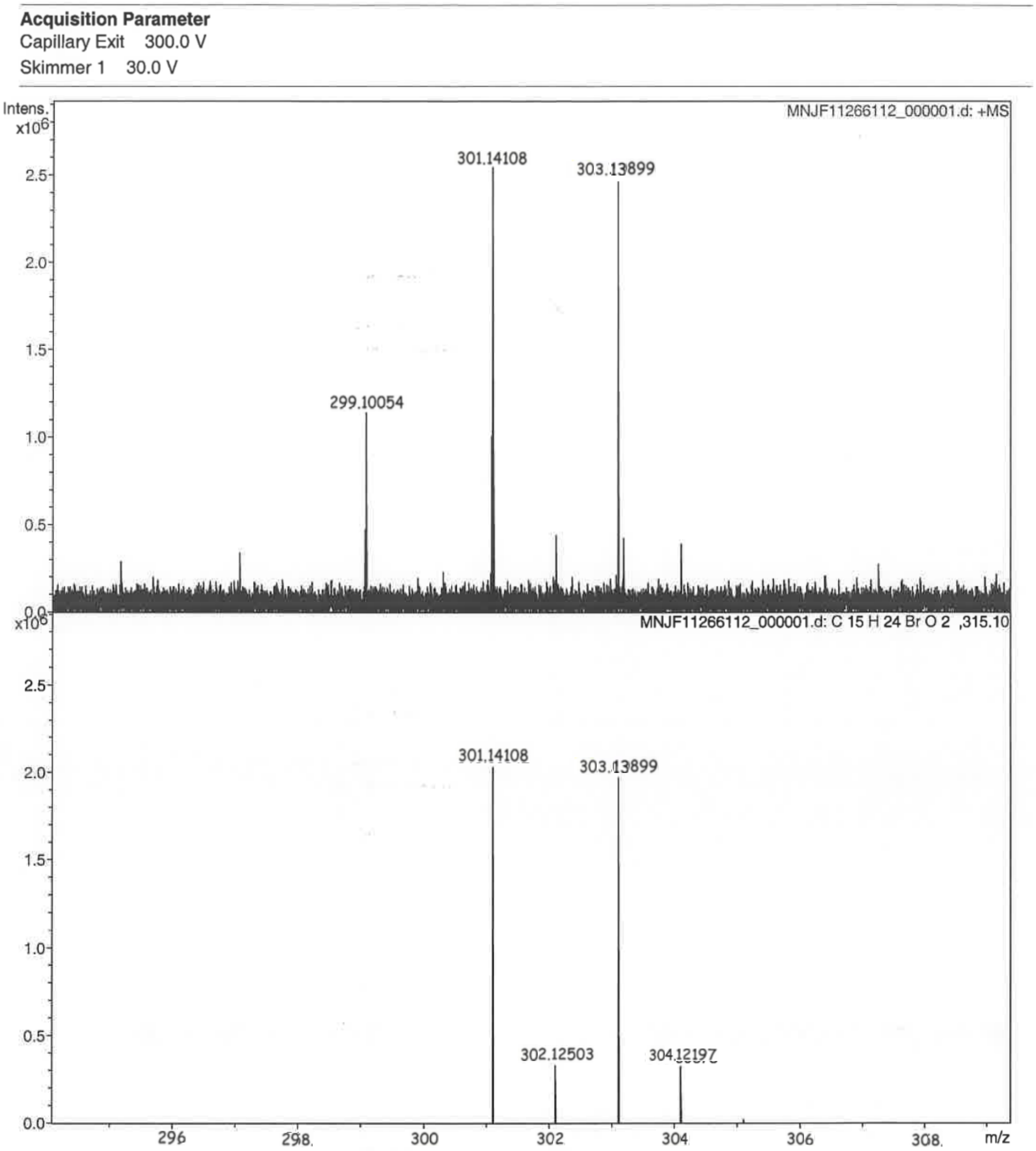

Figure S10. ^{1}H NMR spectrum (600 MHz, $CDCl_3$) of laurocarpane B (**2**).

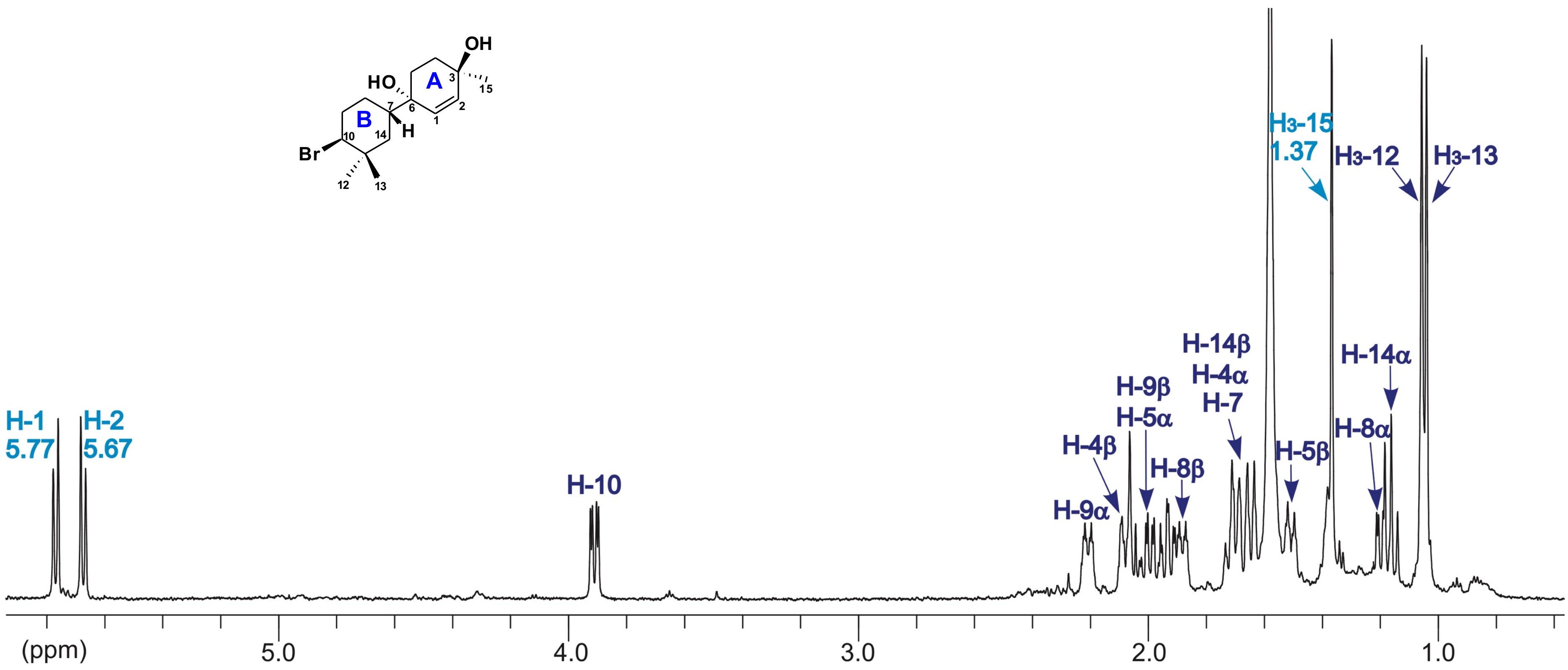

Figure S11. ^{13}C NMR spectrum (150 MHz, $CDCl_3$) of compound **2**.

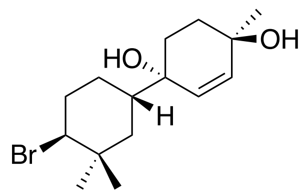

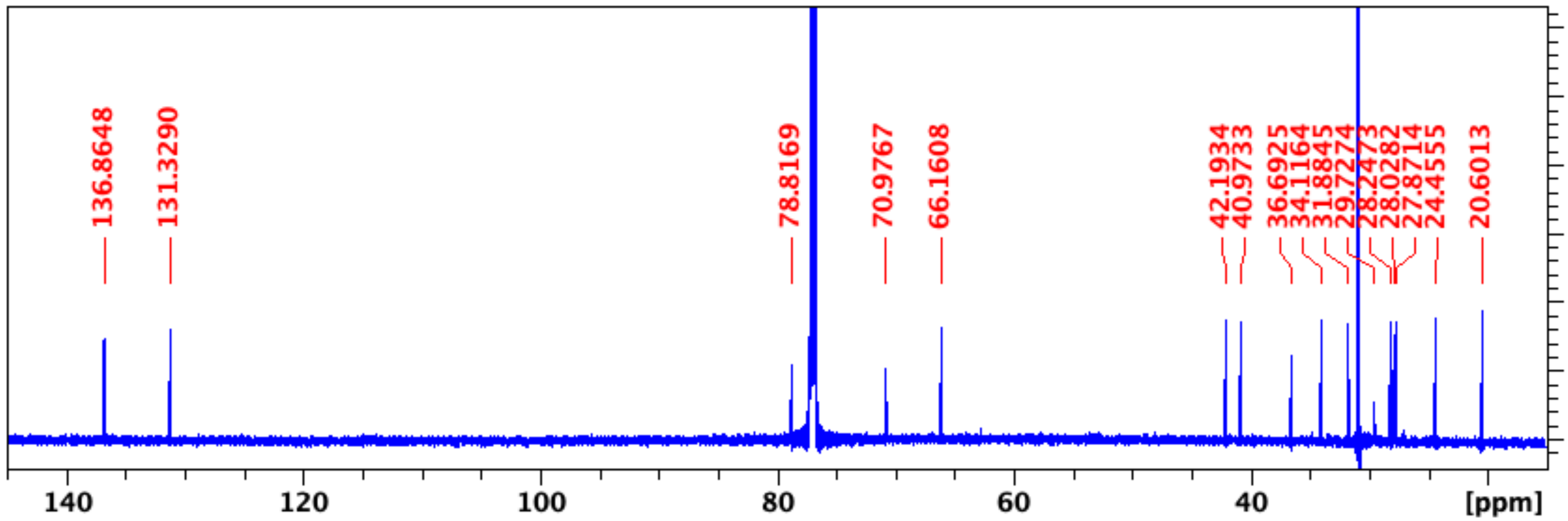

Figure S12. ^{1}H-^{1}H COSY NMR spectrum (600 MHz, $CDCl_3$) of compound **2**.

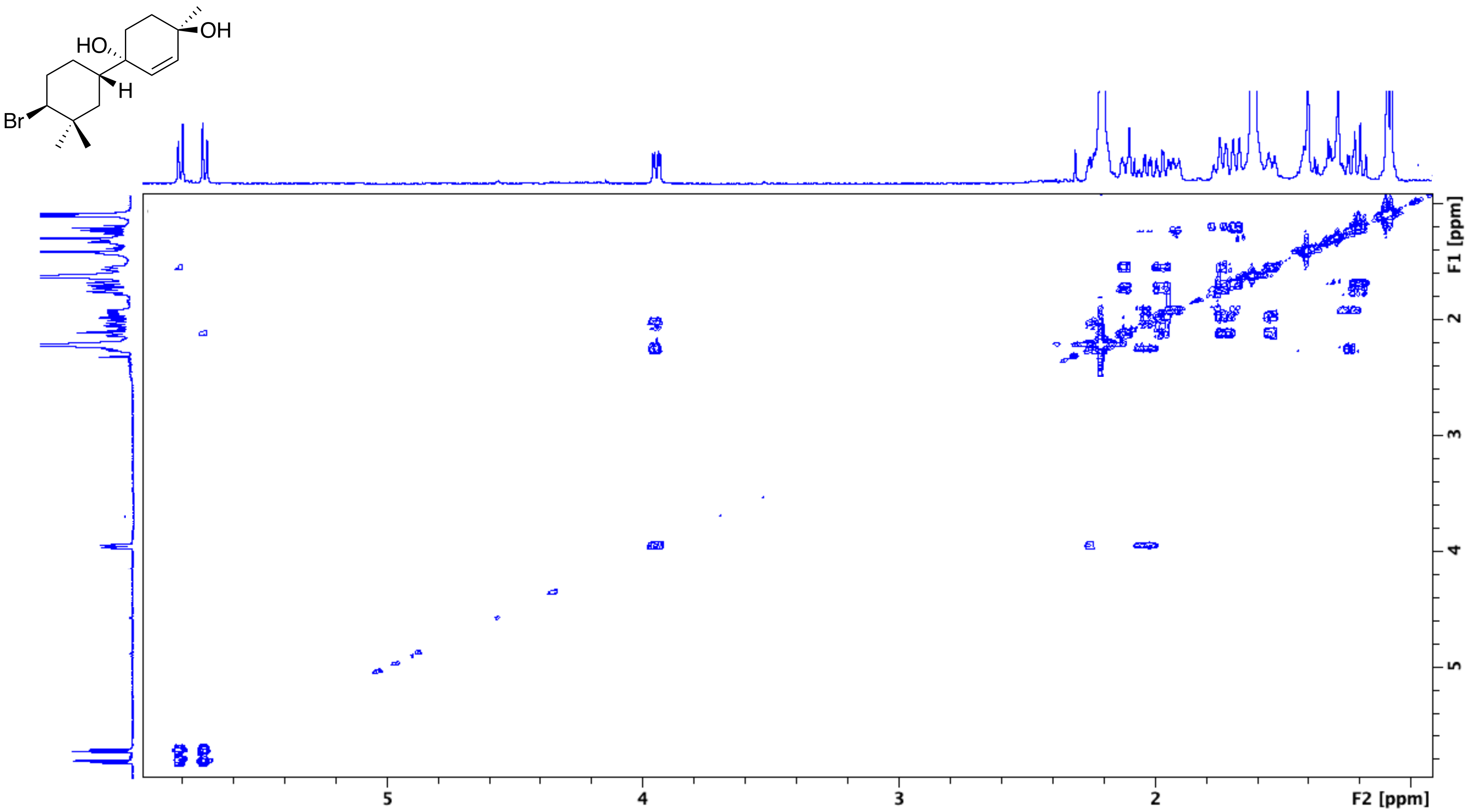

Figure S13. HSQC NMR spectrum (600 MHz, $CDCl_3$) of compound **2**.

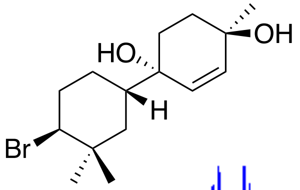

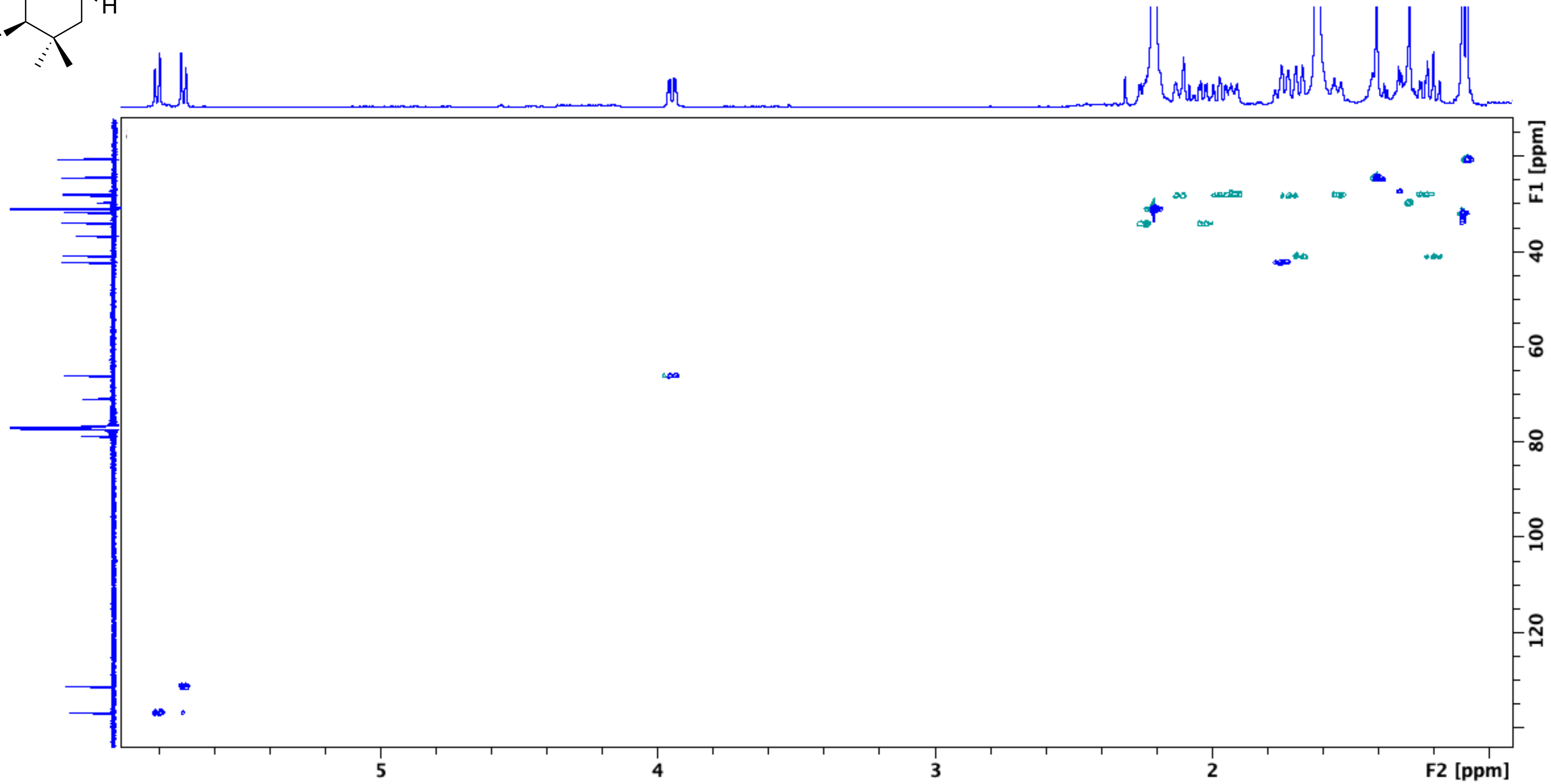

Figure S14. HMBC NMR spectrum (600 MHz, $CDCl_3$) of compound **2**.

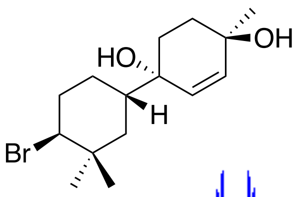

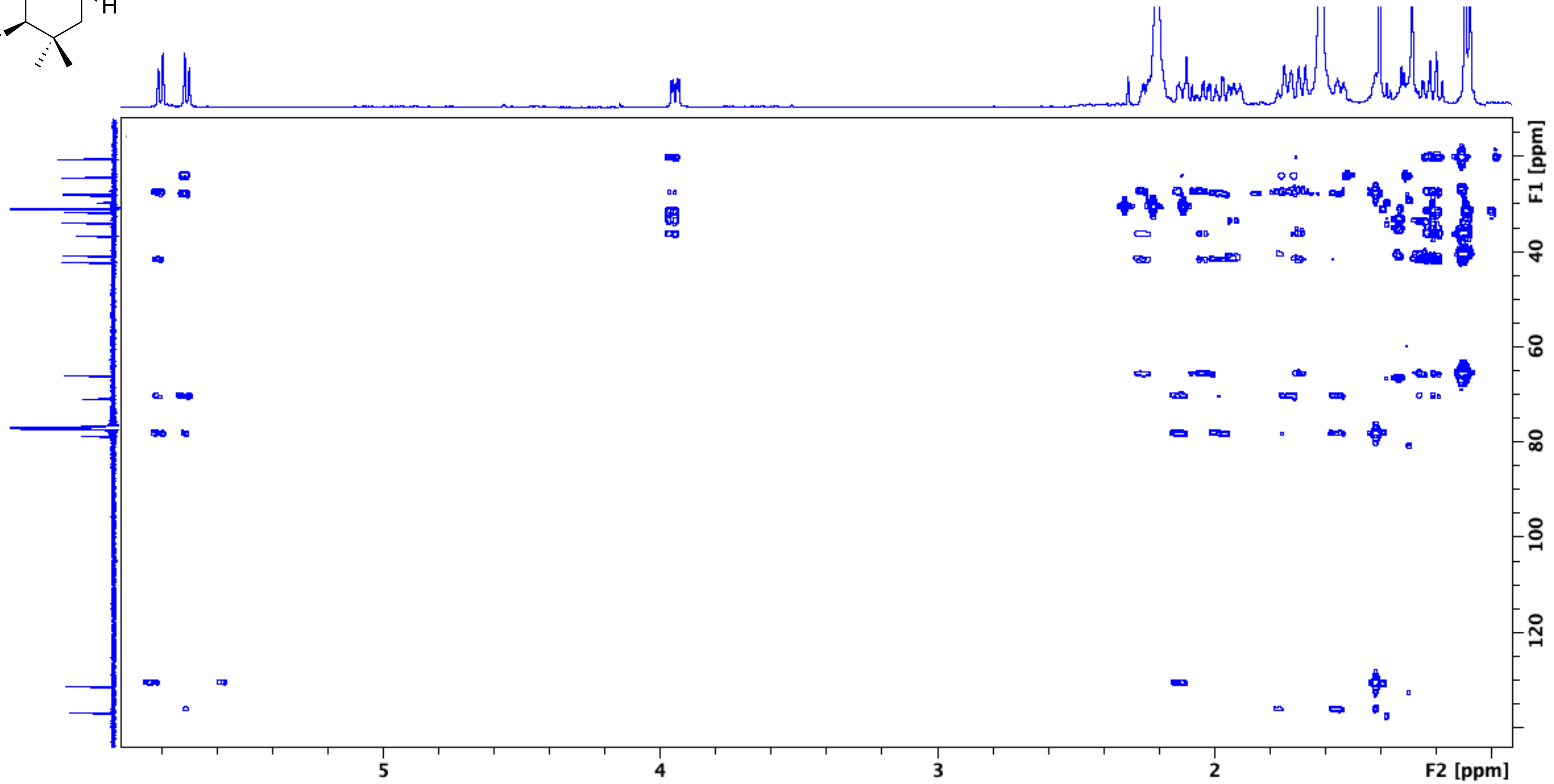

Figure S15. TROESY NMR spectrum (600 MHz, $CDCl_3$) of compound **2**.

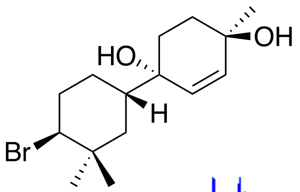

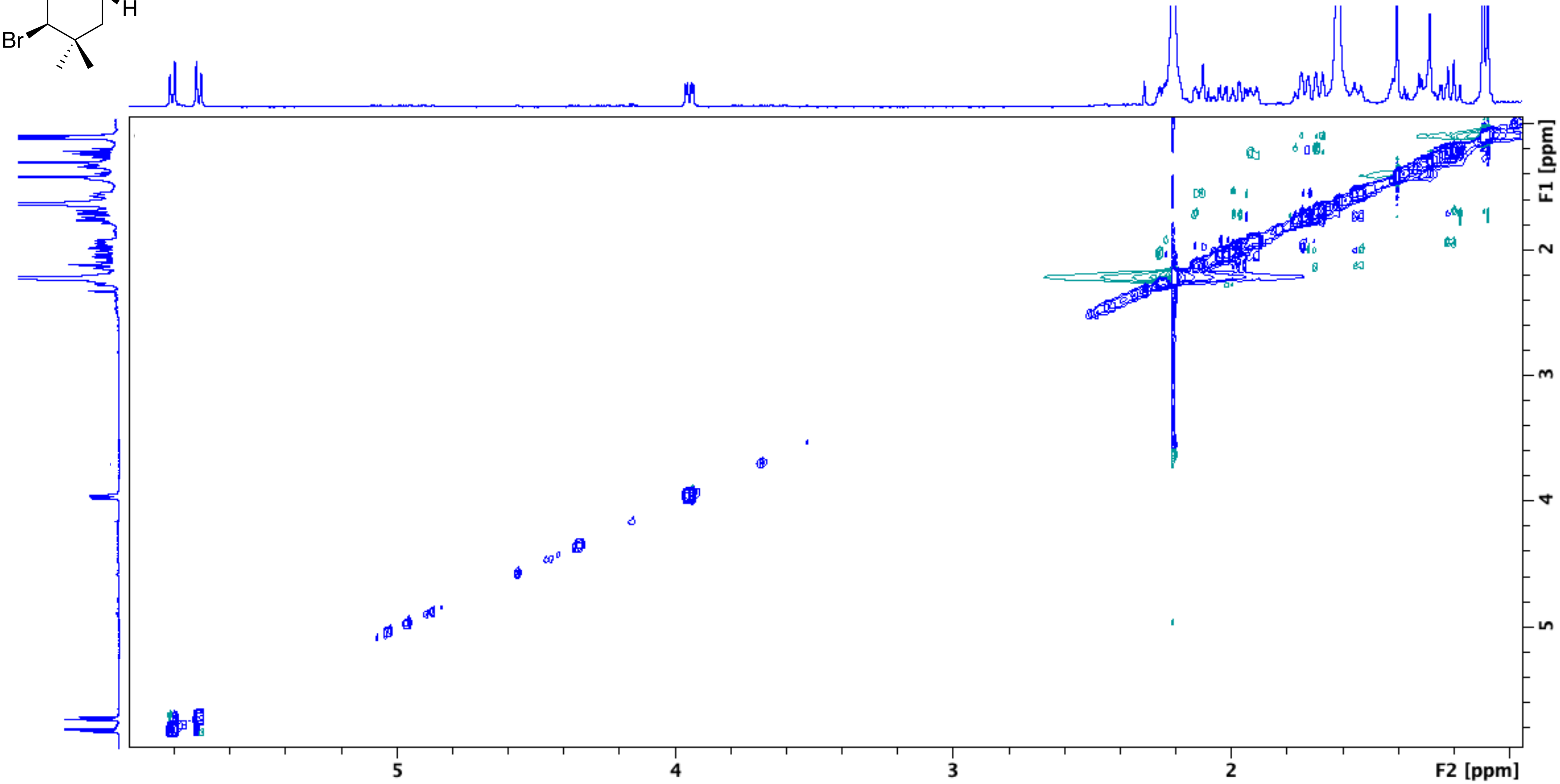

Figure S16. ESI-HRMS mass spectrum for compound **2**.

Multiple Mass Analysis: 6 mass(es) processed

Tolerance = 5.0 PPM / DBE: min = -3.0, max = 120.0
Element prediction: Off
Number of isotope peaks used for i-FIT = 3

Monoisotopic Mass, Even Electron Ions
1773 formula(e) evaluated with 8 results within limits (all results (up to 1000) for each mass)
Elements Used:
C: 0-80 H: 0-80 O: 0-12 Na: 0-1 79Br: 0-1 81Br: 0-1

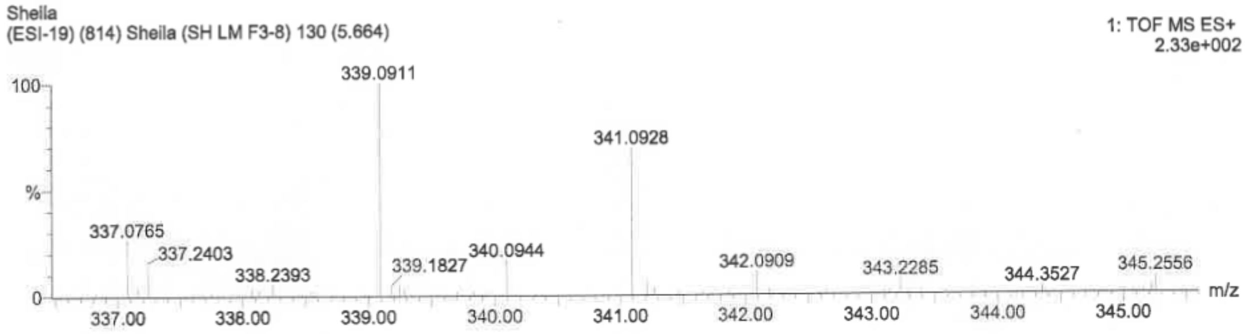

Minimum:	10.00				-3.0			
Maximum:	100.00		5.0	5.0	120.0			
Mass	RA	Calc. Mass	mDa	PPM	DBE	i-FIT	i-FIT (Norm)	Formula
337.0765	26.43	337.0771	-0.6	-1.8	4.5	70.4	0.9	C12 H17 O11
		337.0779	-1.4	-4.2	3.5	70.0	0.5	C15 H23 O2 Na 79Br
337.2403	15.65	---						
339.0911	100.00	339.0936	0.8	2.4	0.5	55.3	0.1	C15 H25 O2 Na 79Br
		339.0927	-1.6	-4.7	3.5	57.4	2.3	C12 H19 O11
340.0944	16.21	---						
341.0928	69.02	341.0939	-1.1	-3.2	5.5	52.4	1.6	C17 H24 O2 81Br
		341.0940	-1.2	-3.5	-2.5	52.1	1.3	C11 H27 O5 Na 79Br
		341.0915	1.3	3.8	2.5	51.4	0.7	C15 H25 O2 Na 81Br
		341.0942	-1.4	-4.1	17.5	54.9	4.2	C24 H14 O Na
342.0909	10.41	---						

Figure S17. ^{1}H NMR spectrum (600 MHz, $CDCl_3$) of laurocarpane C (**3**).

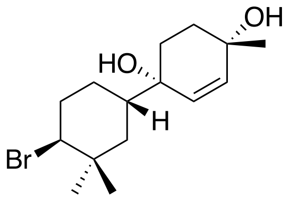

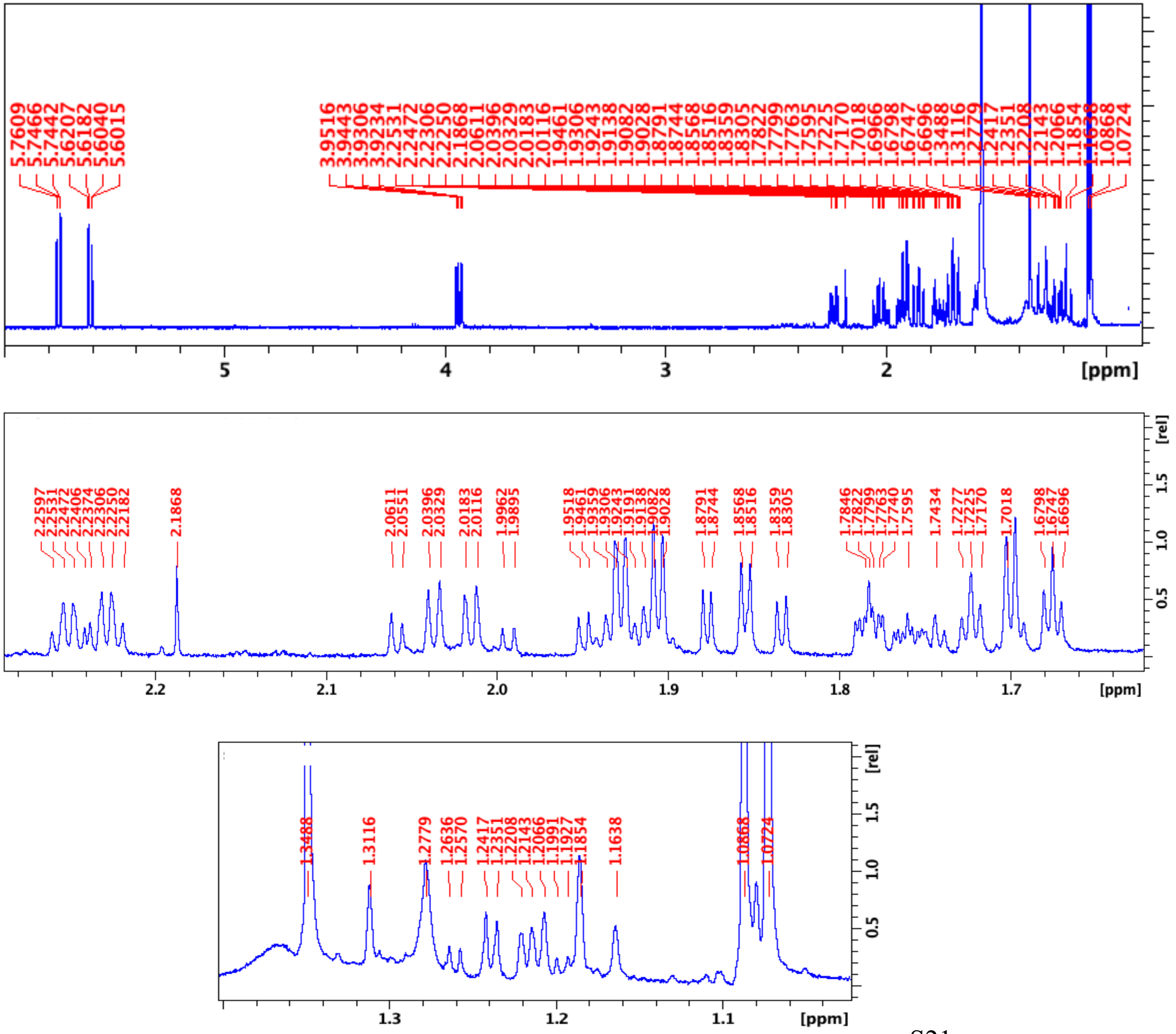

Figure S18. ^{13}C NMR spectrum (150 MHz, $CDCl_3$) of compound **3**.

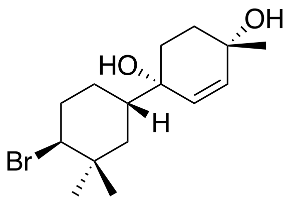

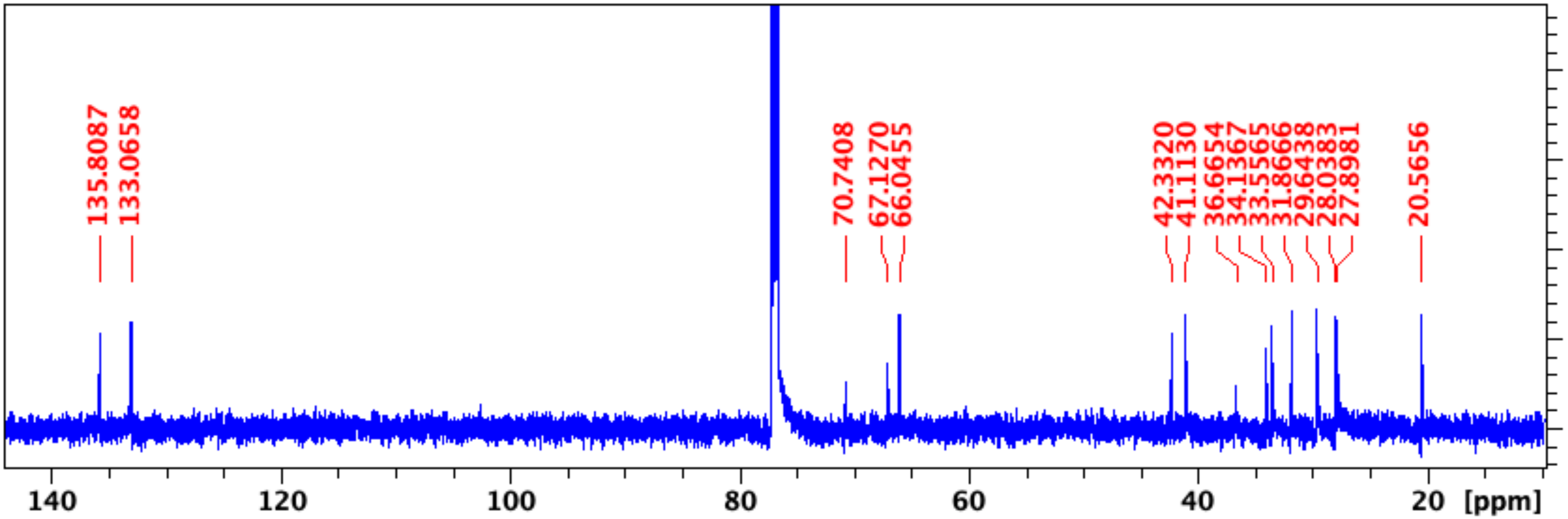

Figure S19. ^{1}H-^{1}H COSY NMR spectrum (600 MHz, $CDCl_3$) of compound **3**.

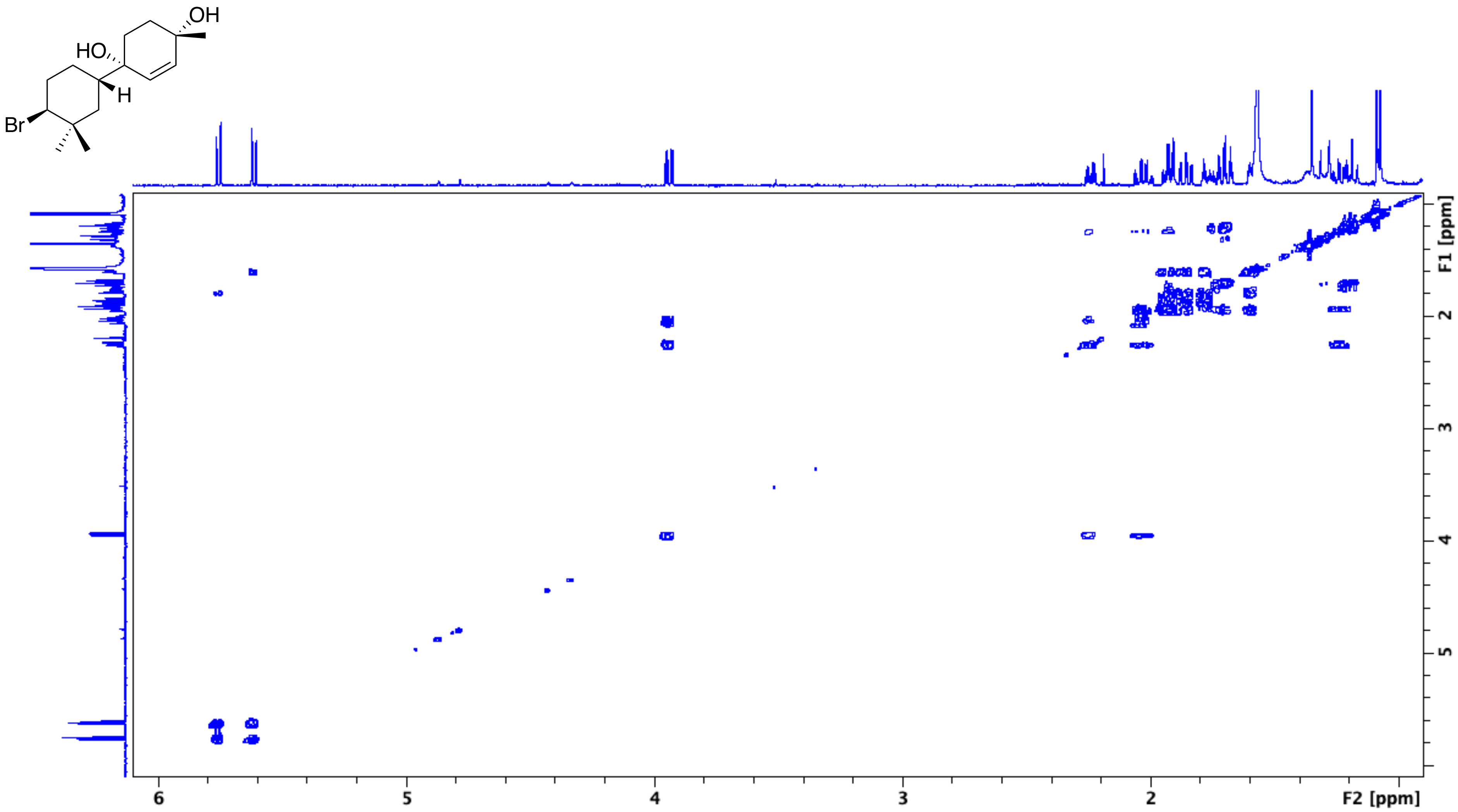

Figure S20. HSQC NMR spectrum (600 MHz, $CDCl_3$) of compound **3**.

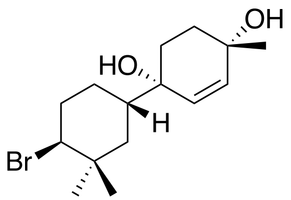

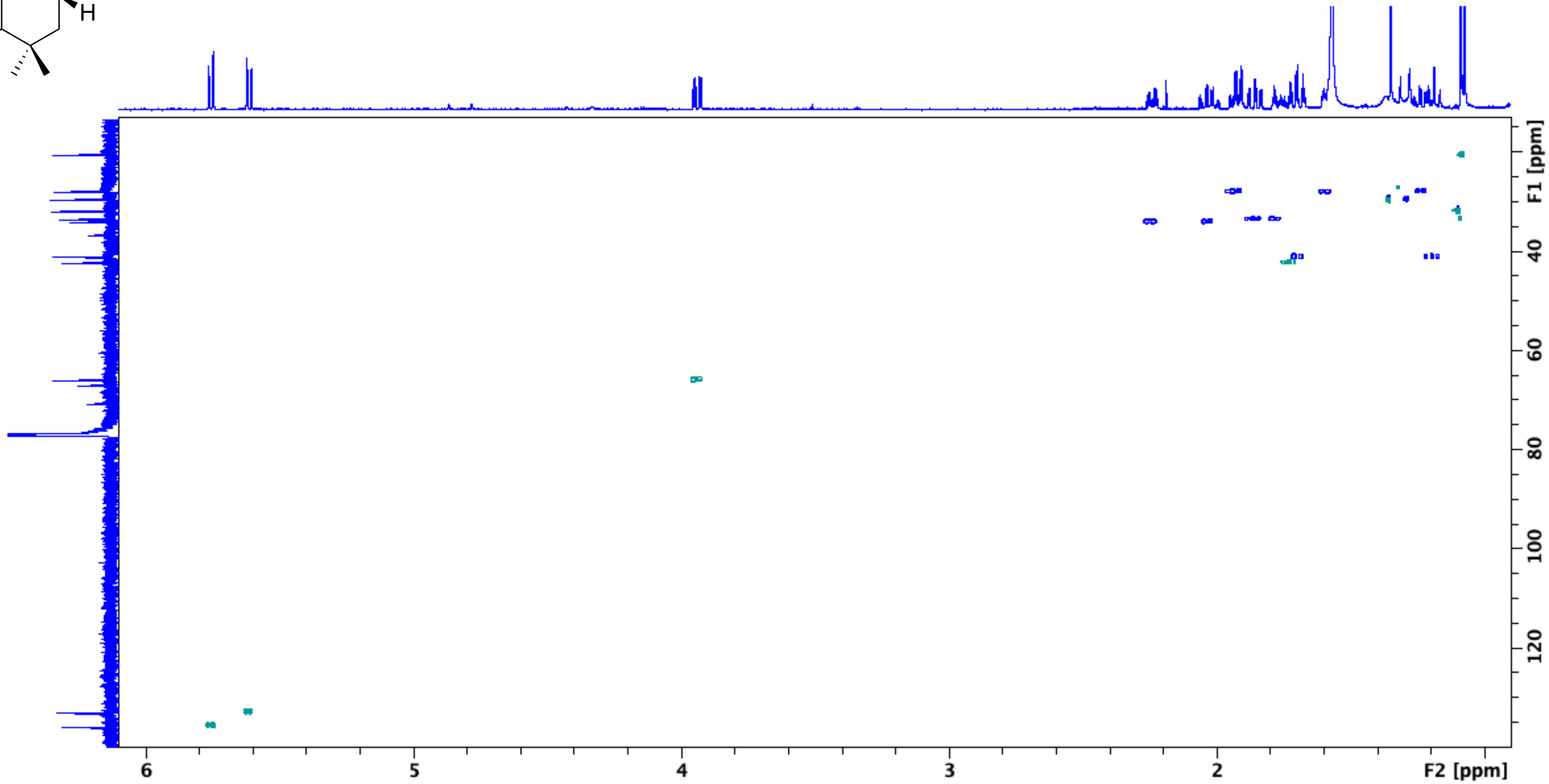

Figure S21. HMBC NMR spectrum (600 MHz, $CDCl_3$) of compound **3**.

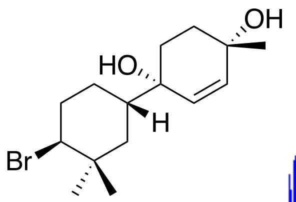

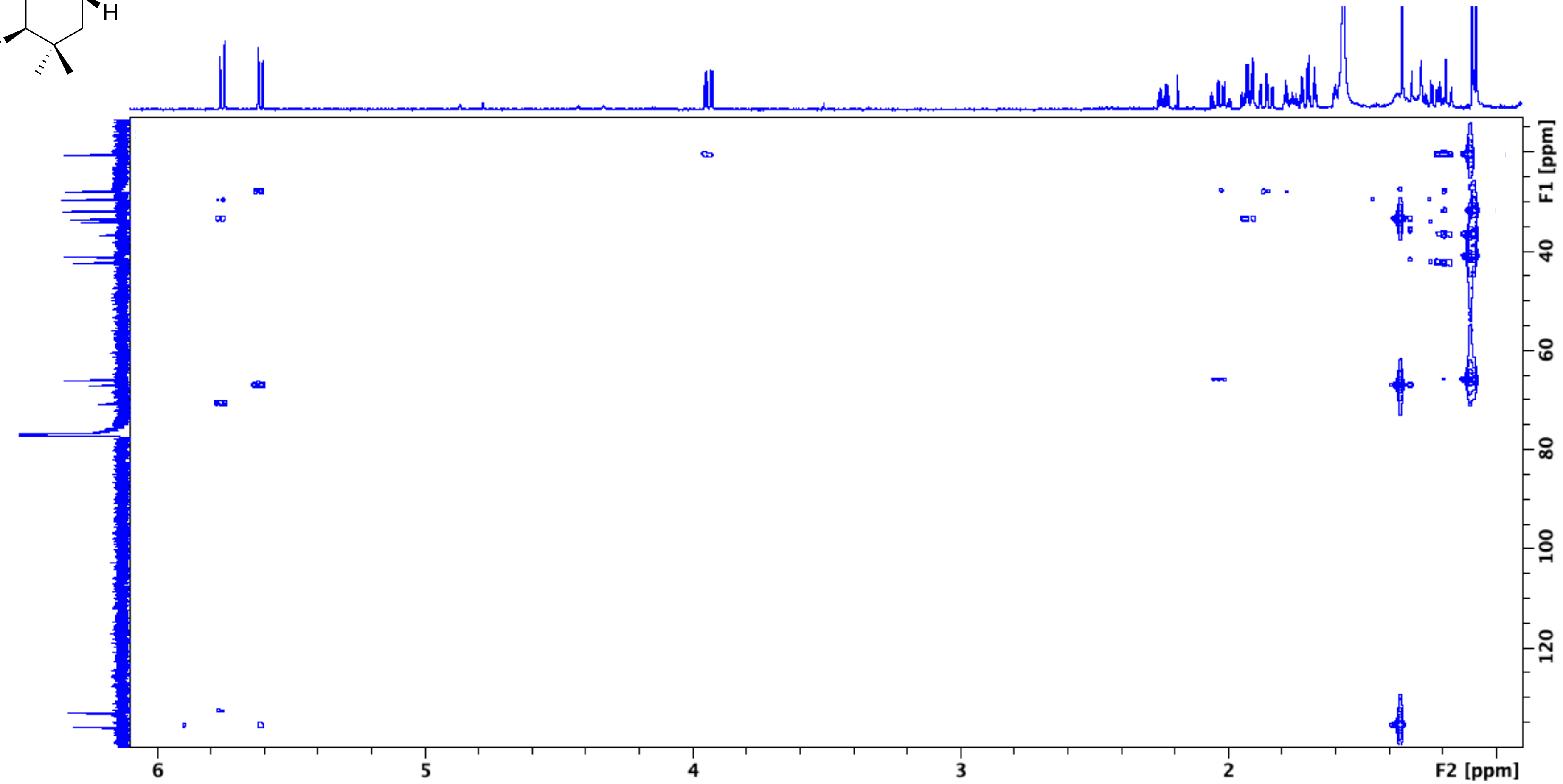

Figure S22. ESI-HRMS mass spectrum for compound **3**.

Multiple Mass Analysis: 5 mass(es) processed
Tolerance = 5.0 PPM / DBE: min = -3.0, max = 120.0
Element prediction: Off
Number of isotope peaks used for i-FIT = 3

Monoisotopic Mass, Even Electron Ions
1481 formula(e) evaluated with 5 results within limits (all results (up to 1000) for each mass)
Elements Used:
C: 0-80 H: 0-80 O: 0-12 Na: 0-1 79Br: 0-1 81Br: 0-1

Sheila
(ESI-19) (816) Sheila (SH LM F4-5) 9 (0.385)

1: TOF MS ES+
5.54e+002

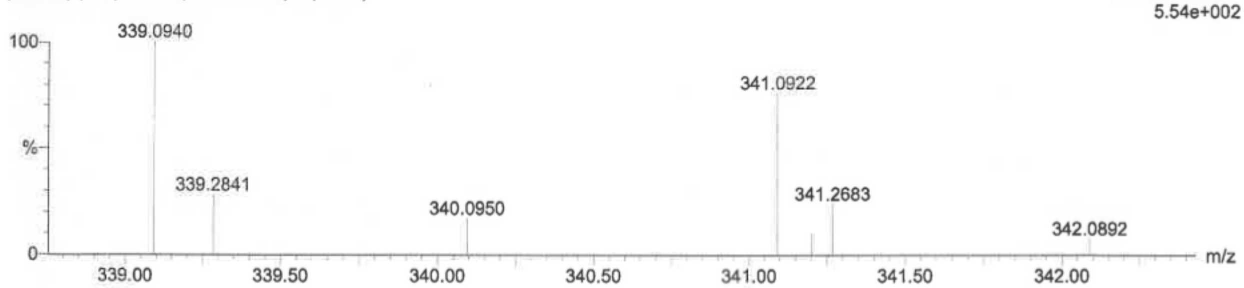

Minimum:	10.00				-3.0			
Maximum:	100.00		5.0	5.0	120.0			
Mass	RA	Calc. Mass	mDa	PPM	DBE	i-FIT	i-FIT (Norm)	Formula
339.0940	100.00	339.0936	0.4	1.2	2.5	49.7	1.4	C15 H25 O2 Na 79Br
		339.0927	1.3	3.8	3.5	48.6	0.3	C12 H19 O11
339.2841	27.87	---						
340.0950	16.96	---						
341.0922	76.05	341.0915	0.7	2.1	2.5	33.2	0.0	C15 H25 O2 Na 81Br
341.2683	23.77	341.2692	-0.9	-2.6	2.5	27.0	0.7	C20 H37 O4
		341.2668	1.5	4.4	-0.5	27.1	0.7	C18 H38 O4 Na

Figure S23. ^{1}H NMR spectrum (600 MHz, $CDCl_3$) of laurocarpane D (**4**).

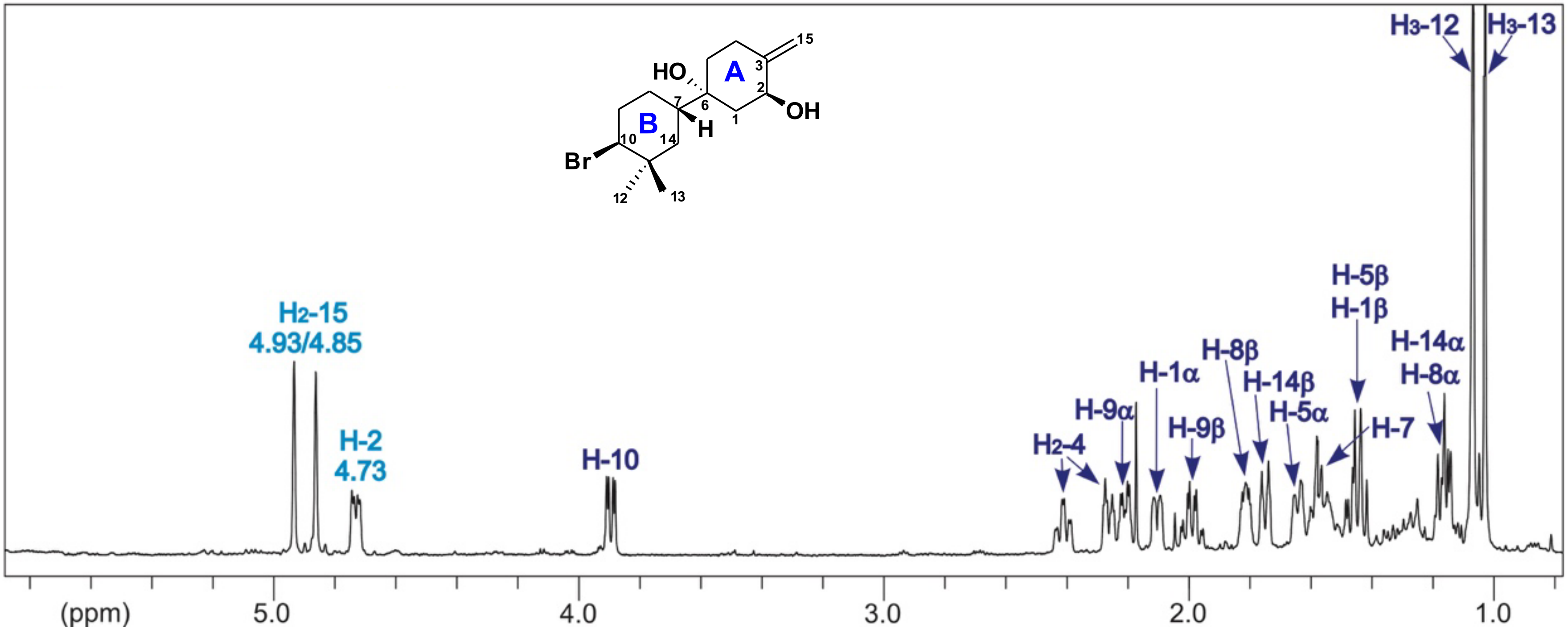

Figure S24. ^{13}C NMR spectrum (150 MHz, $CDCl_3$) of compound **4**.

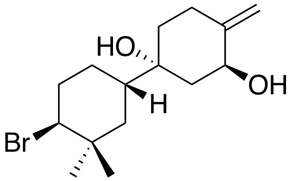

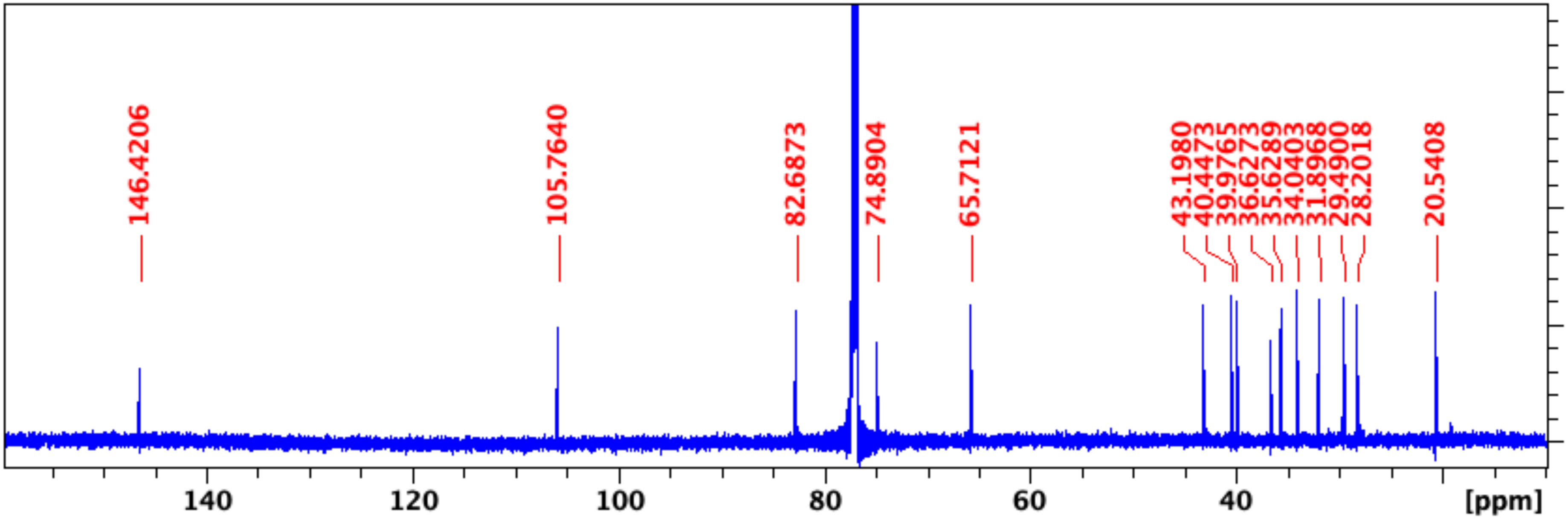

Figure S25. ^{1}H-^{1}H COSY NMR spectrum (600 MHz, $CDCl_3$) of compound **4**.

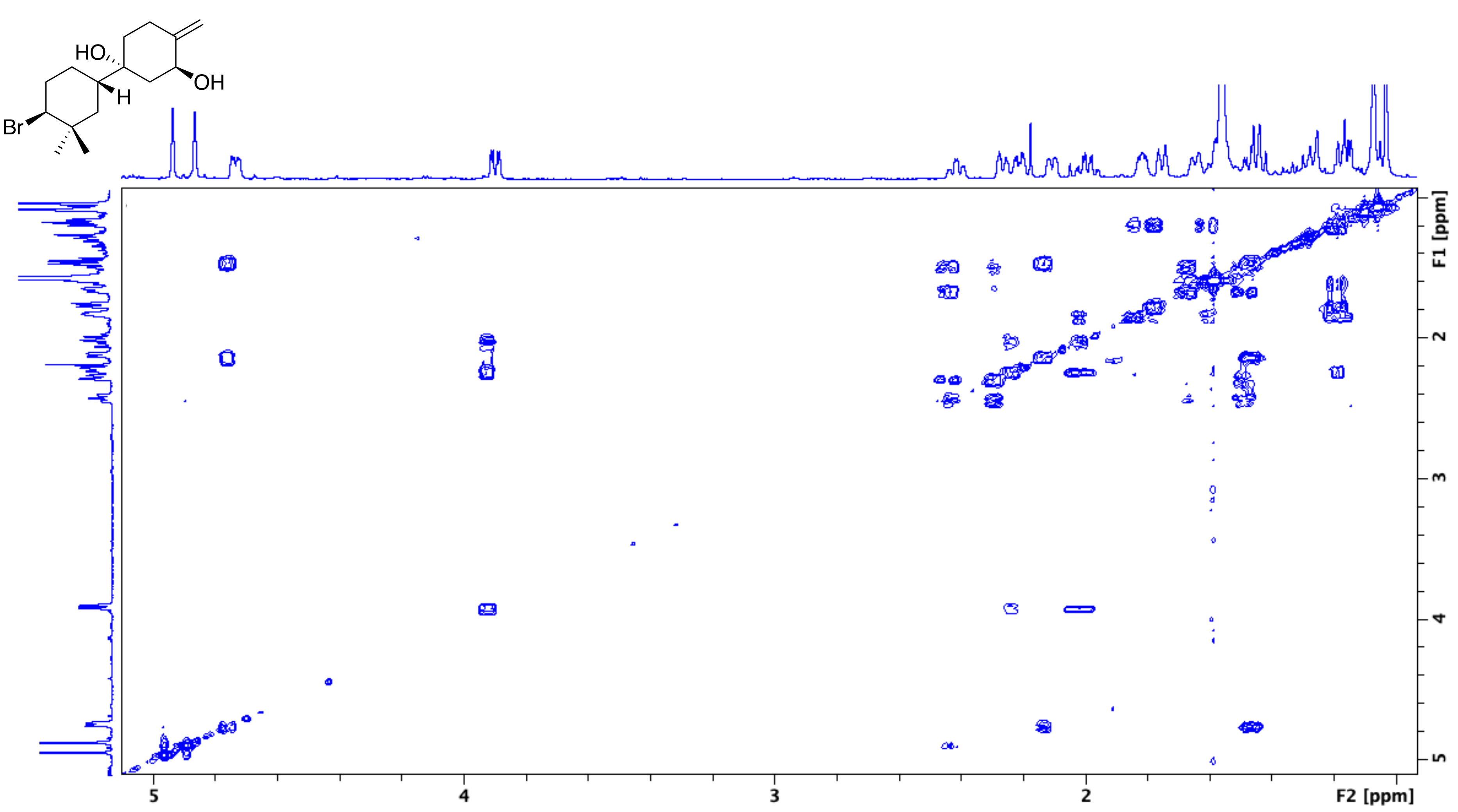

Figure S26. HSQC NMR spectrum (600 MHz, $CDCl_3$) of compound **4**.

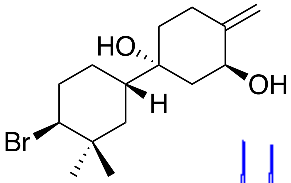

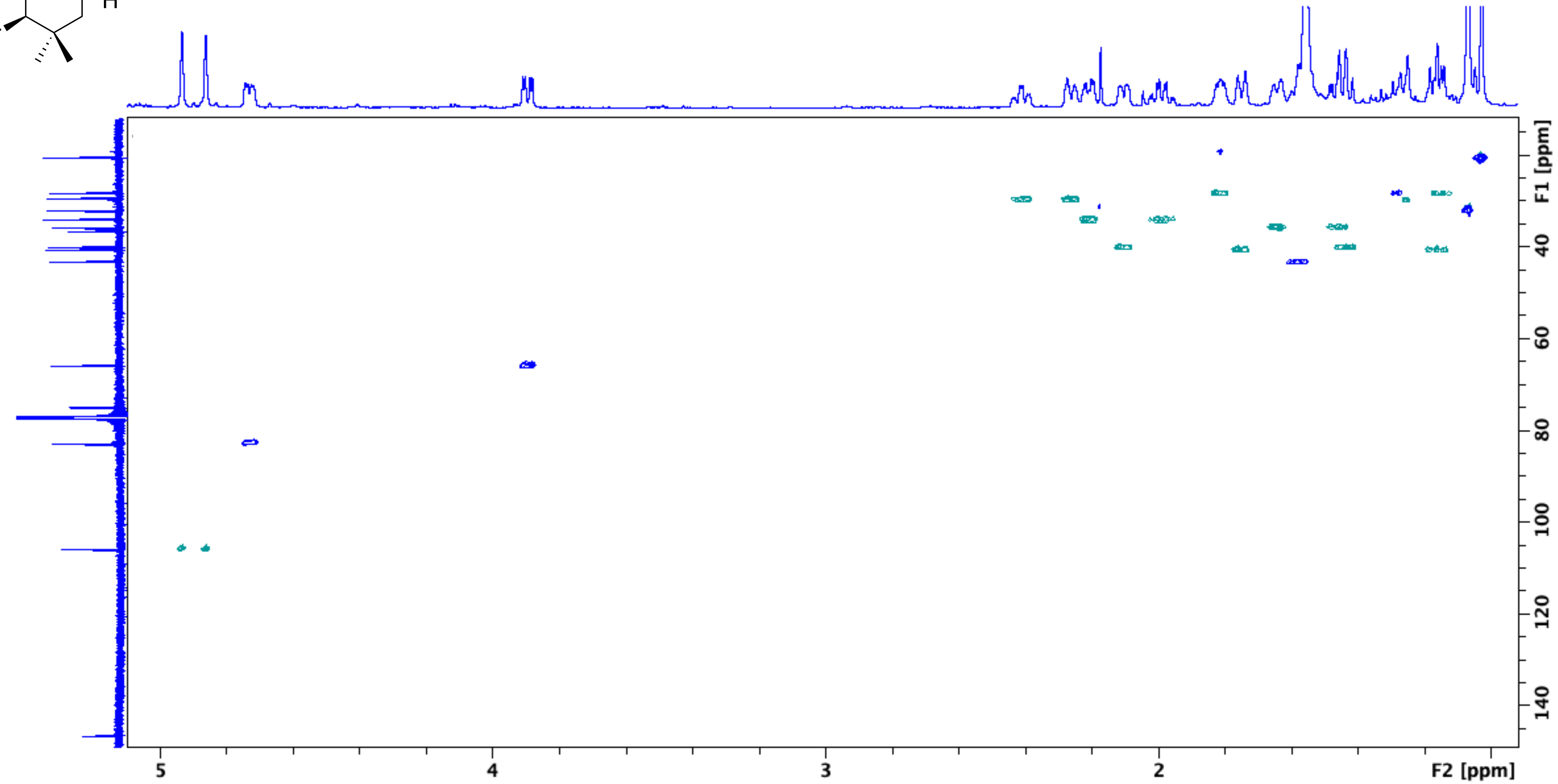

Figure S27. HMBC NMR spectrum (600 MHz, $CDCl_3$) of compound **4**.

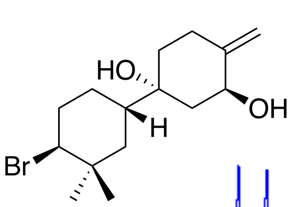

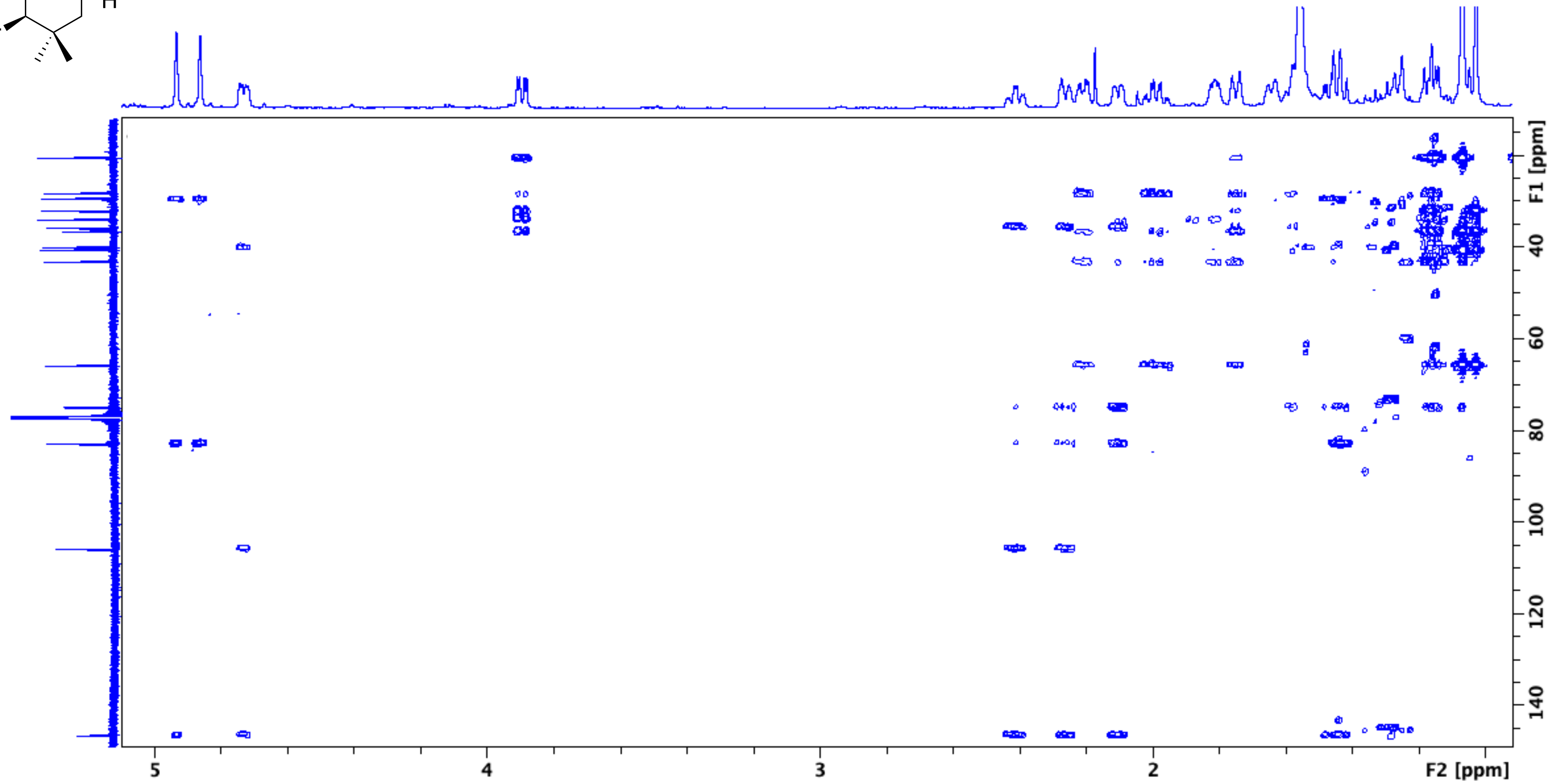

Figure S28. TROESY NMR spectrum (600 MHz, $CDCl_3$) of compound **4**.

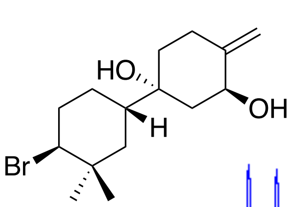

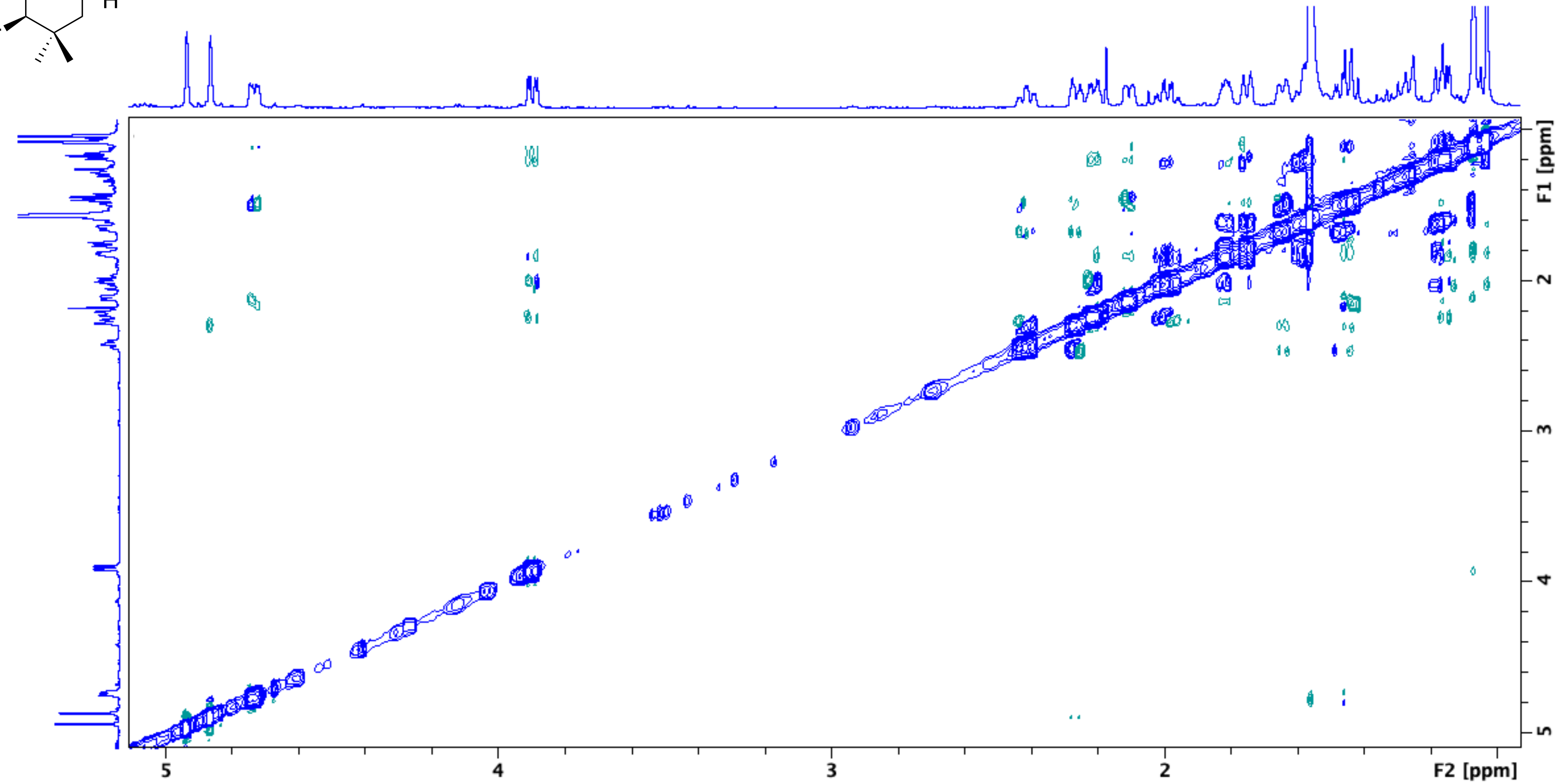

Figure S29. ESI-FTICR mass spectrum for compound **4**.

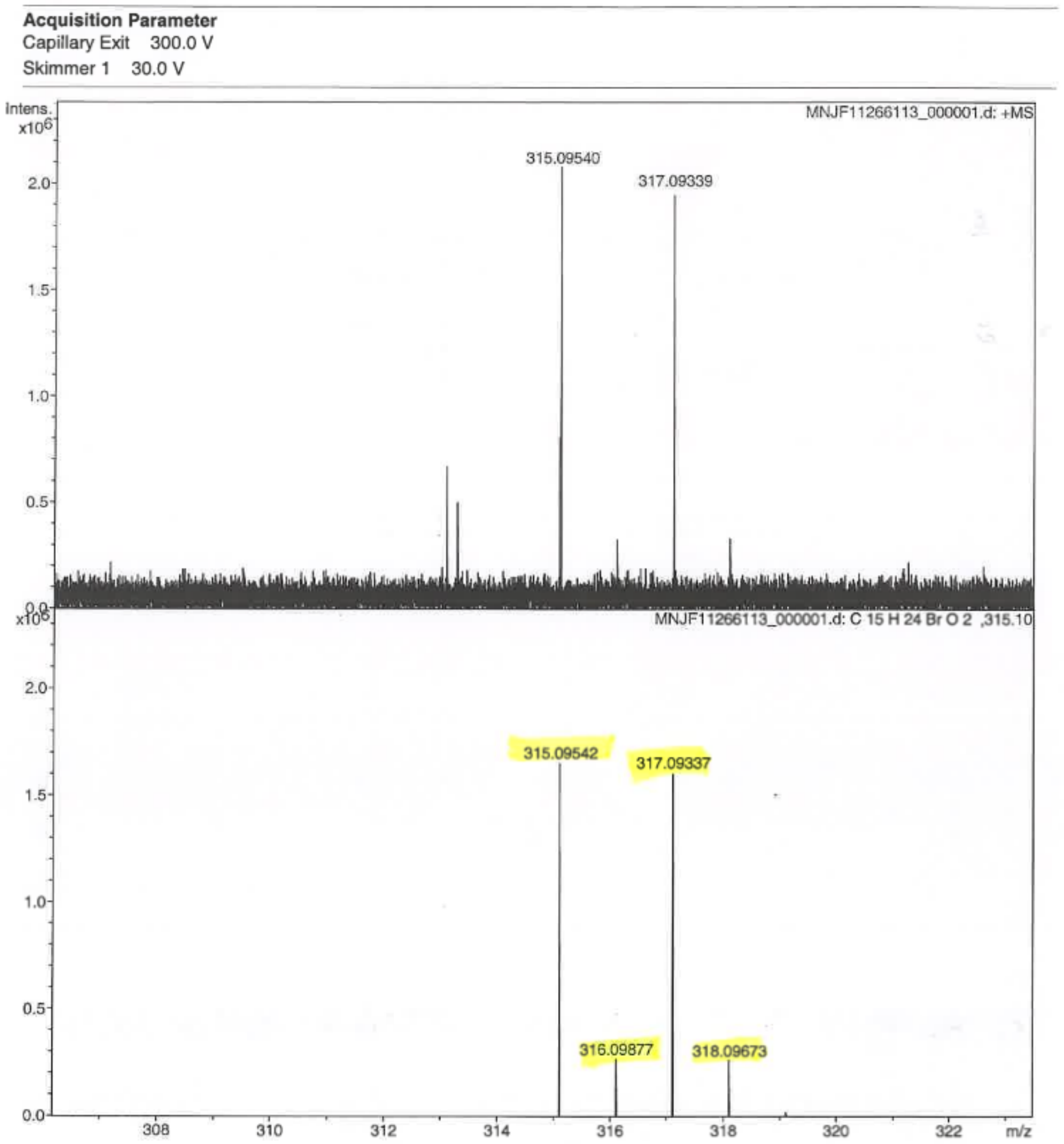

Figure S30. ^{1}H NMR spectrum (600 MHz, $CDCl_3$) of laurocarpane E (**5**).

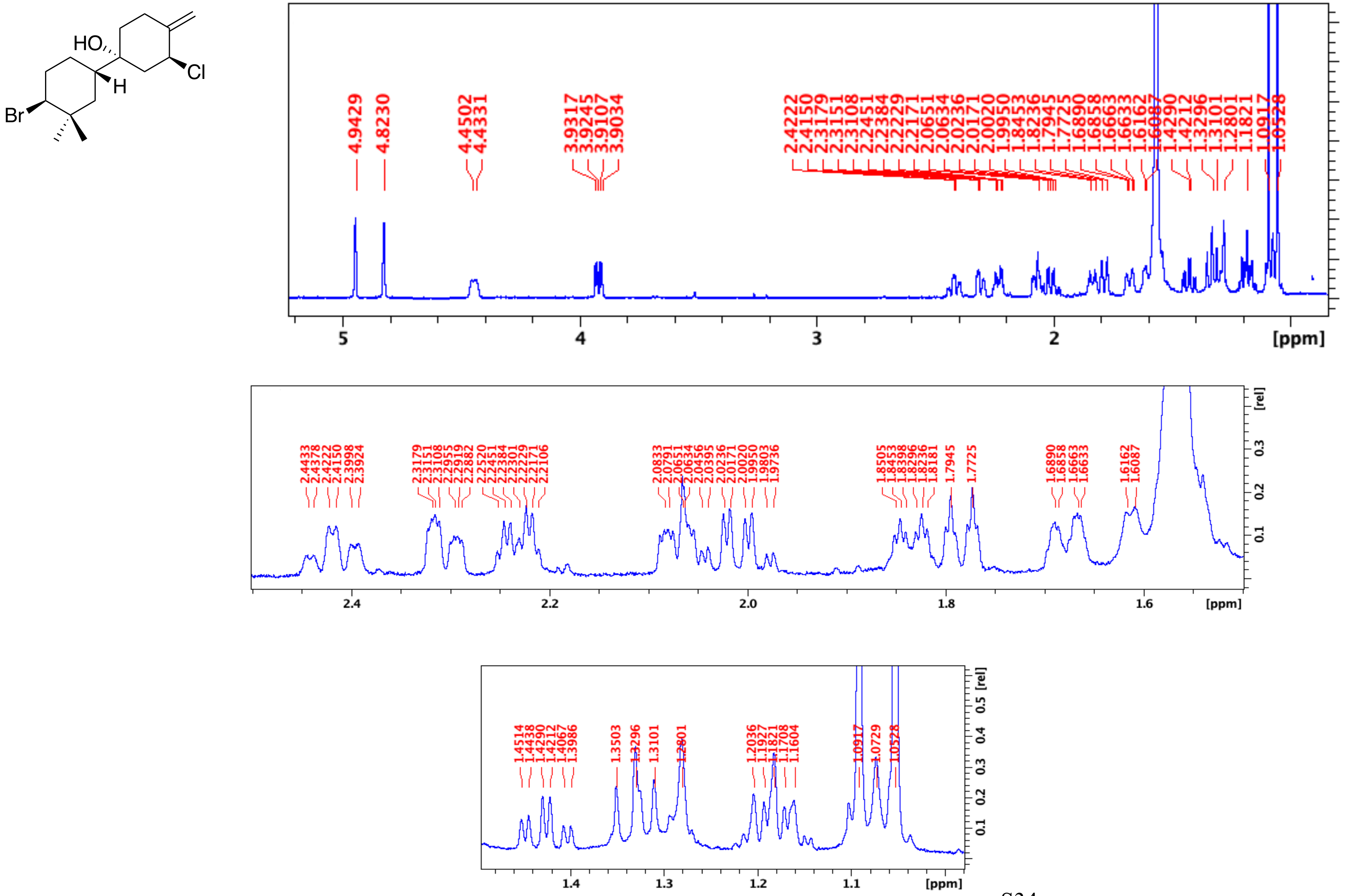

Figure S31. ^{1}H-^{1}H COSY NMR spectrum (600 MHz, $CDCl_3$) of compound **5**.

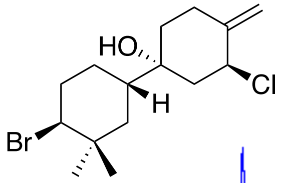

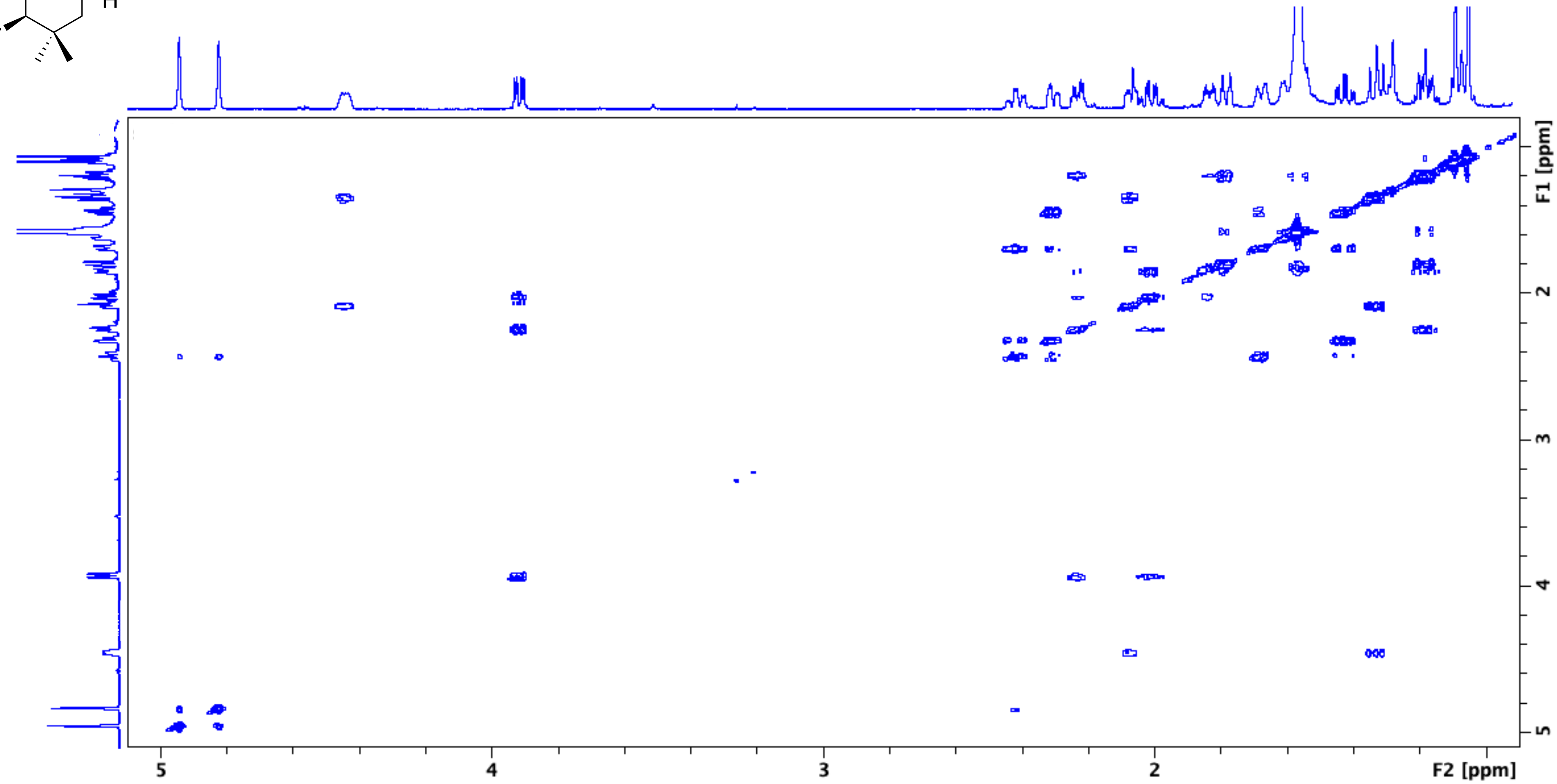

Figure S32. HSQC NMR spectrum (600 MHz, $CDCl_3$) of compound **5**.

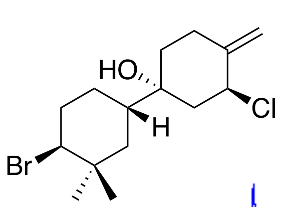

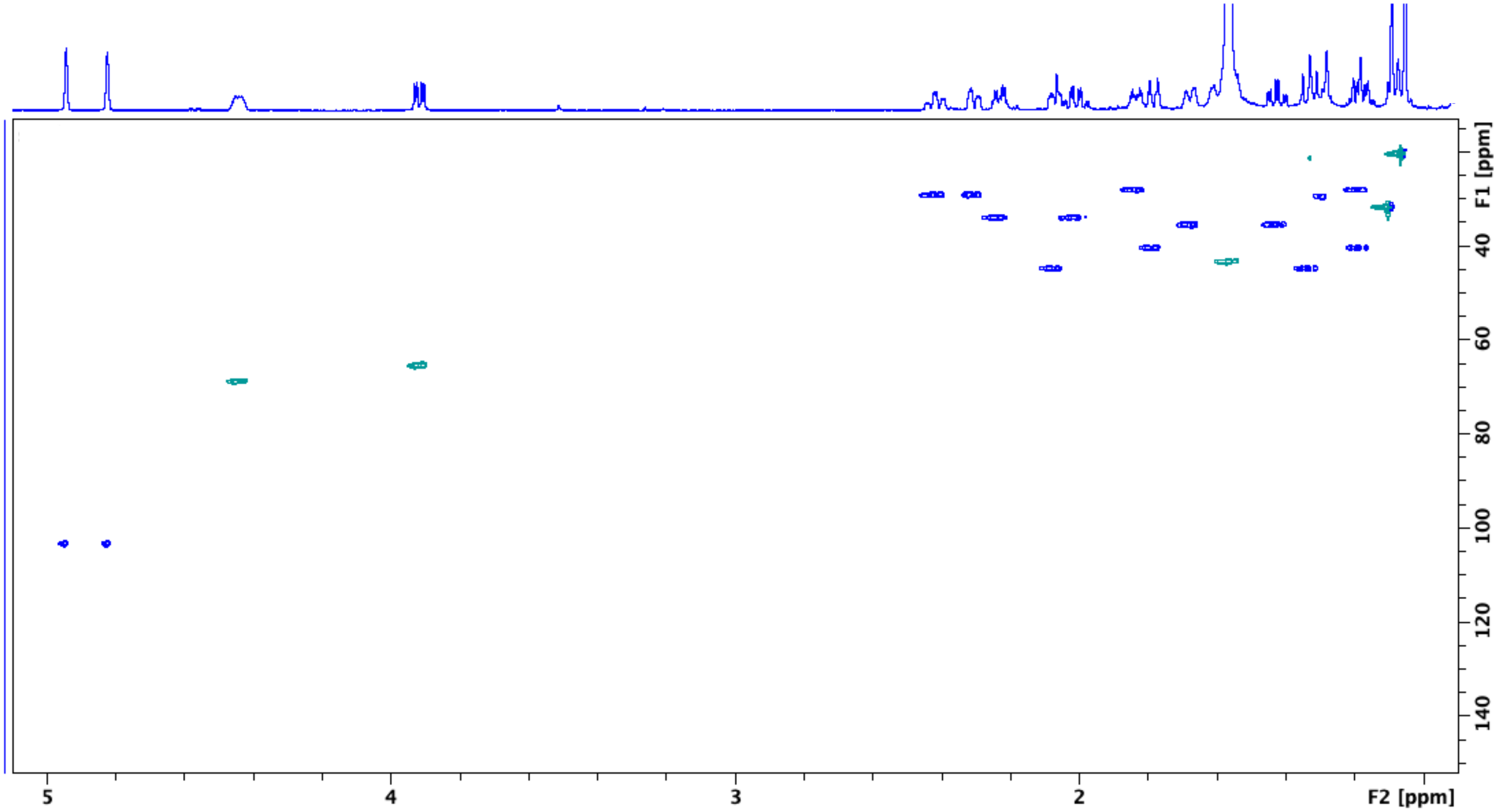

Figure S33. HMBC NMR spectrum (600 MHz, $CDCl_3$) of compound **5**.

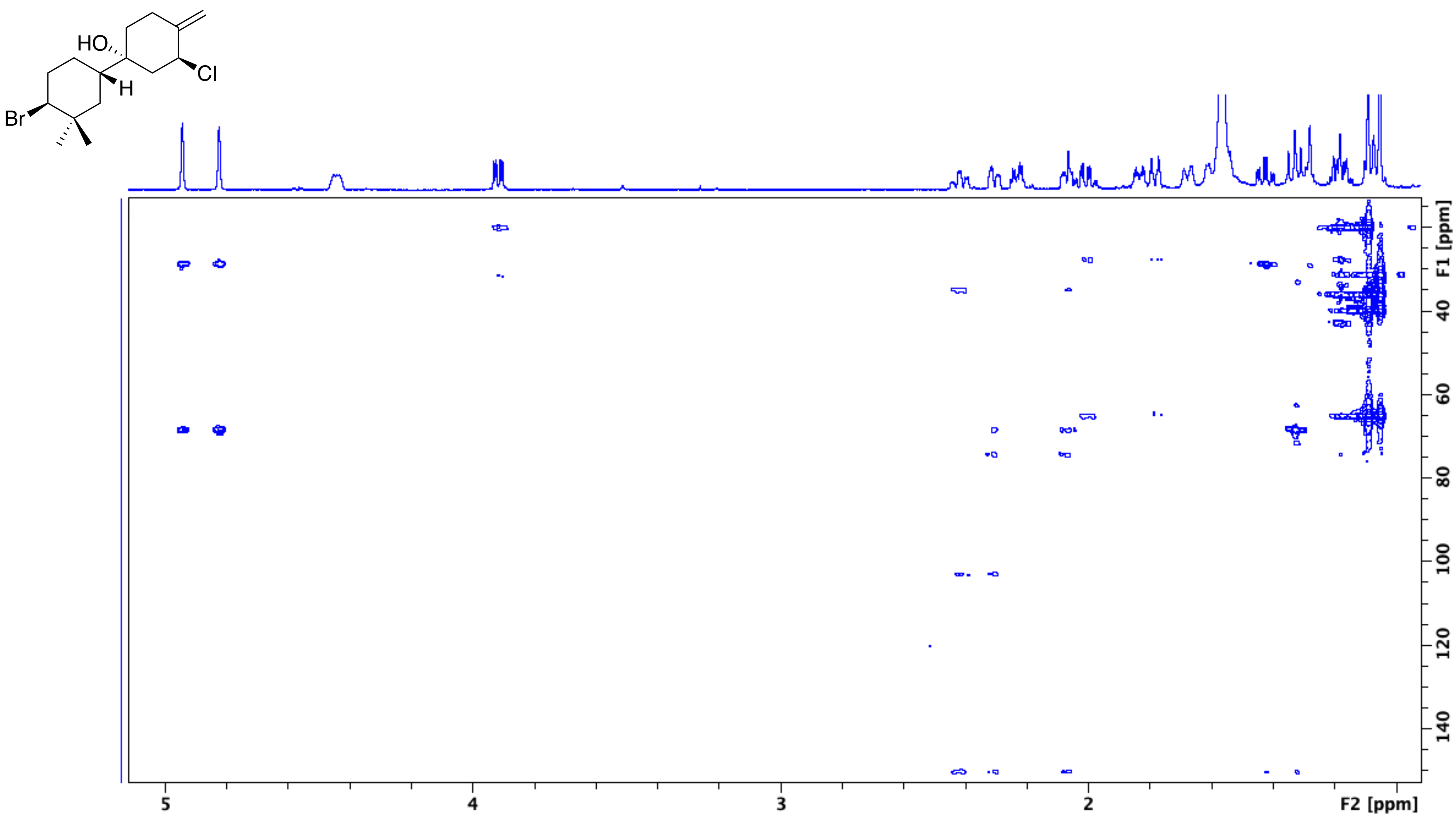

Figure S34. ESI-HRMS mass spectrum for compound **5**.

Multiple Mass Analysis: 3 mass(es) processed
Tolerance = 5.0 PPM / DBE: min = -3.0, max = 120.0
Element prediction: Off
Number of isotope peaks used for i-FIT = 3

Monoisotopic Mass, Even Electron Ions
962 formula(e) evaluated with 6 results within limits (all results (up to 1000) for each mass)
Elements Used:
C: 0-60 H: 0-120 O: 0-3 Na: 0-1 35Cl: 0-1 37Cl: 0-1 79Br: 0-1 81Br: 0-1

Sheila
(ESI-19) (814) Sheila (SH LM F3-8) 72 (3.120)

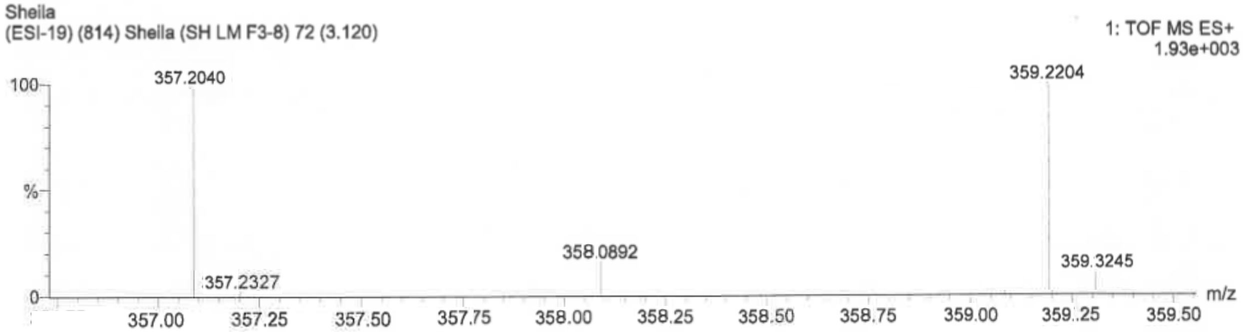

Minimum:	10.00				-3.0			
Maximum:	100.00		5.0	5.0	120.0			

Mass	RA	Calc. Mass	mDa	PPM	DBE	i-FIT	i-FIT (Norm)	Formula
357.2040	96.50	357.0597	-0.1	-0.3	3.5	44.0	0.8	C15 H24 O2 Na 35Cl 79Br
		357 0885	-0.9	-2.5	2.5	44.8	1.6	C1r H24 O2 Na 35Cl 79Br
		357 0860	1.6	4.5	7.5	44.7	1.5	C19 H22 Na 81Br
358.0892	15.73	---						
359.2204	100.00	359.2198	0.3	0.8	9.5	36.2	0.7	C20 H22 O 79Br
		359.0577	-0.7	-2.0	2.5	36.3	0.7	C15 H24 O2 Na 37Cl 81Br

Figure S35. ^{1}H NMR spectrum (600 MHz, $CDCl_3$) of laurocarpane F (**6**).

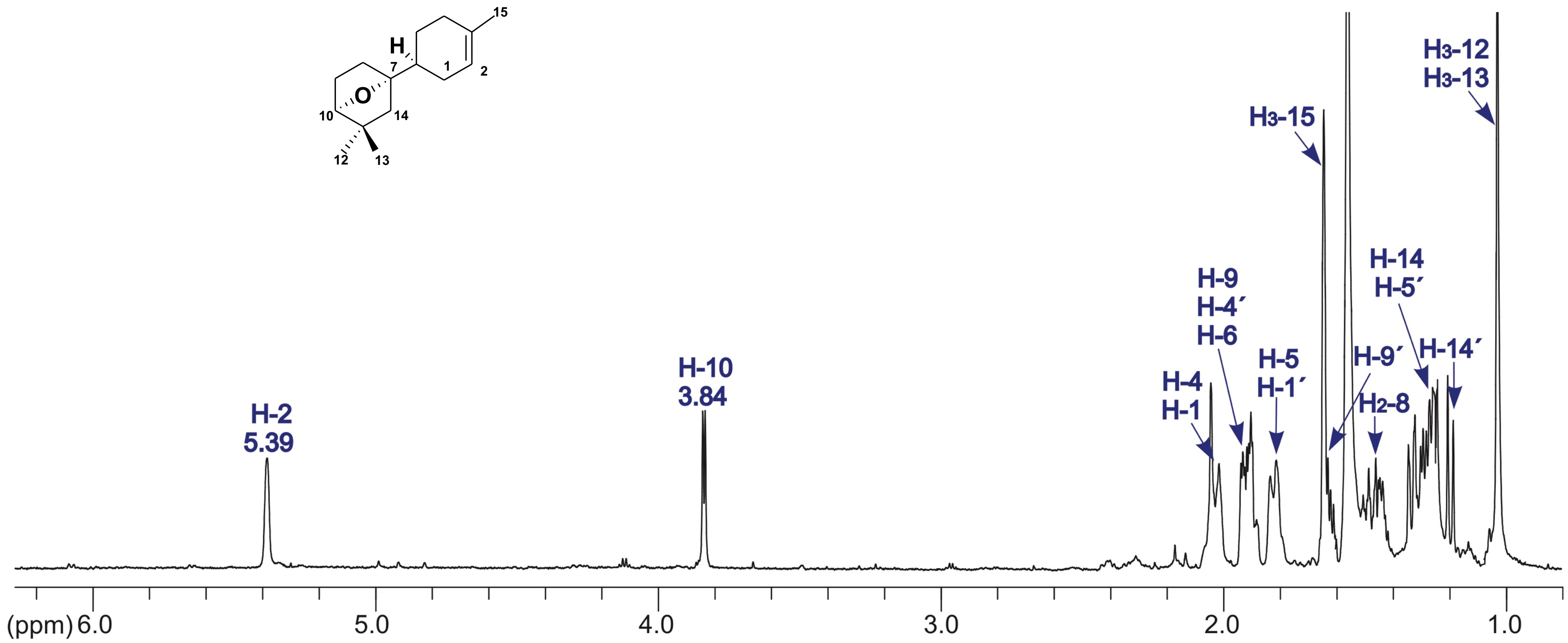

Figure S36. ^{13}C NMR spectrum (150 MHz, $CDCl_3$) of compound **6**.

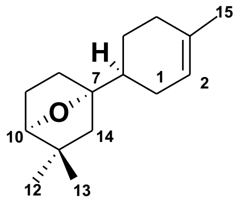

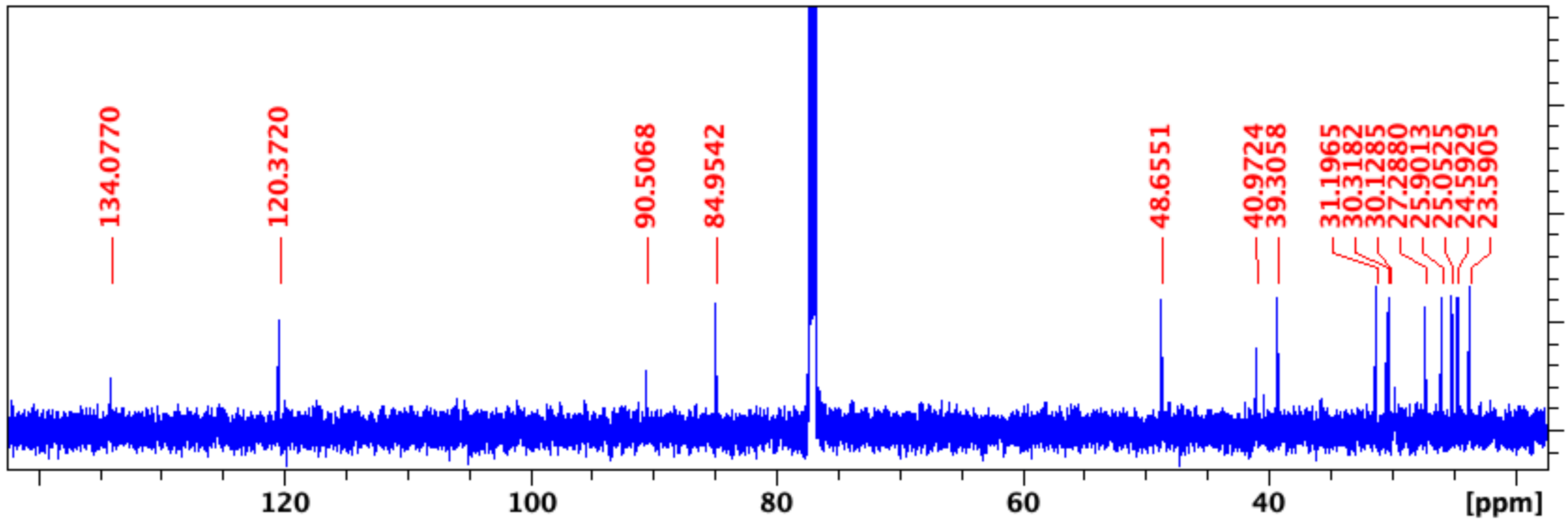

Figure S37. ^{1}H-^{1}H COSY NMR spectrum (600 MHz, $CDCl_3$) of compound **6**.

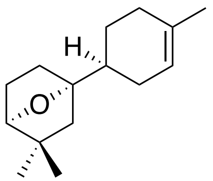

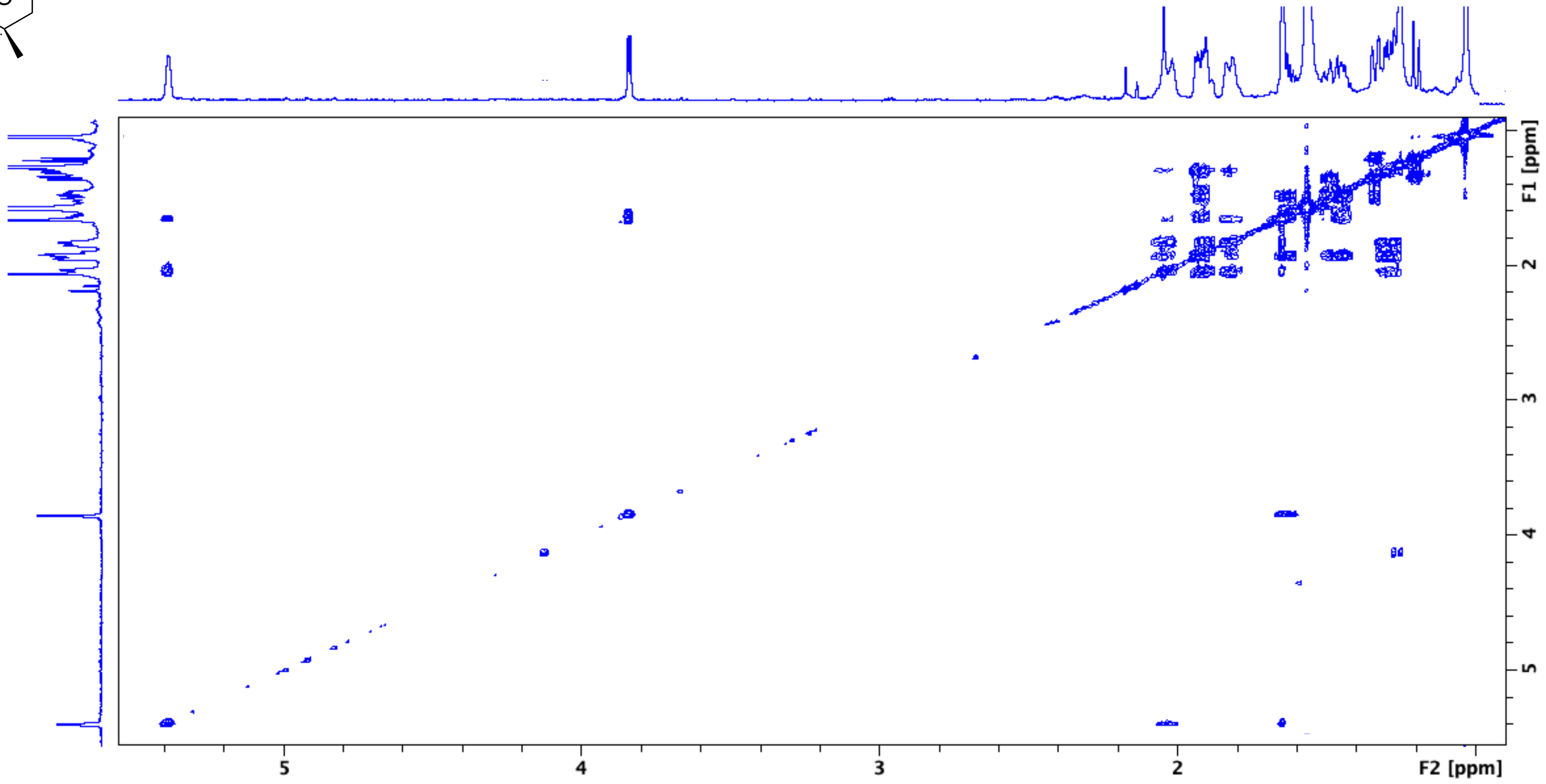

Figure S38. HSQC NMR spectrum (600 MHz, $CDCl_3$) of compound **6**.

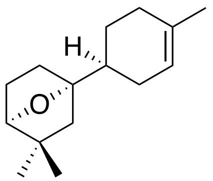

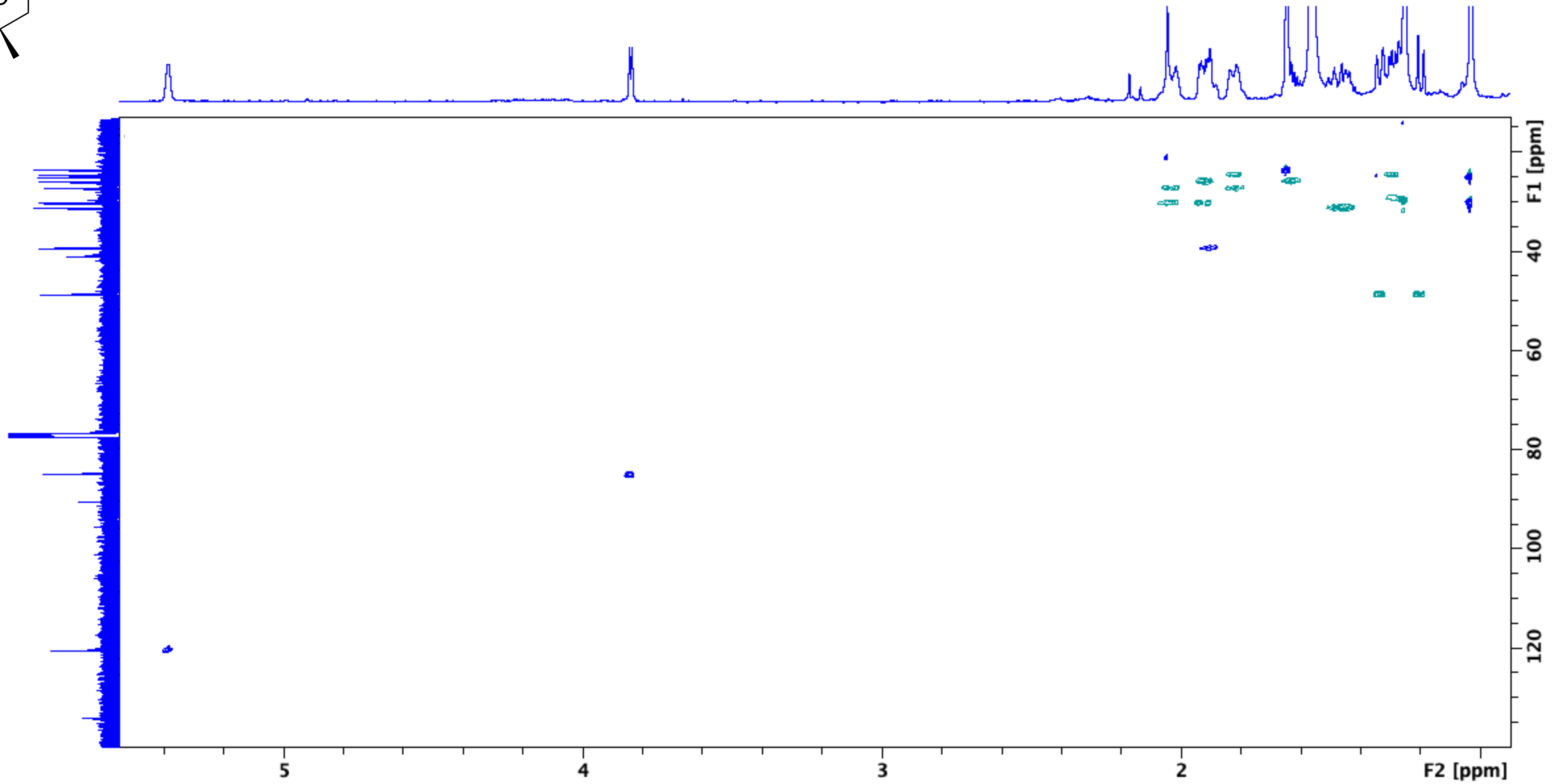

Figure S39. HMBC NMR spectrum (600 MHz, $CDCl_3$) of compound **6**.

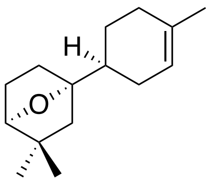

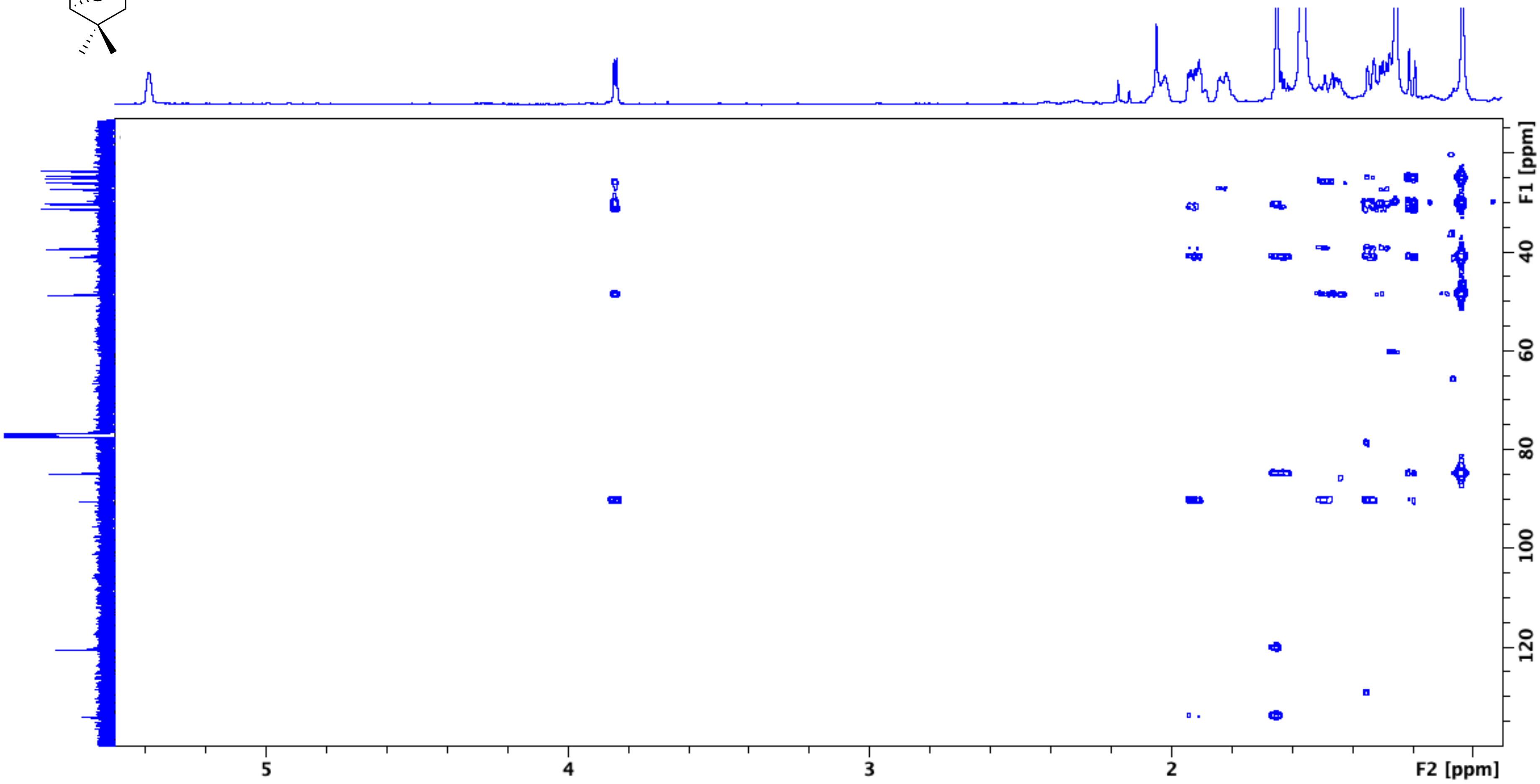

Figure S40. ESI-FTICR mass spectrum for compound **6**.

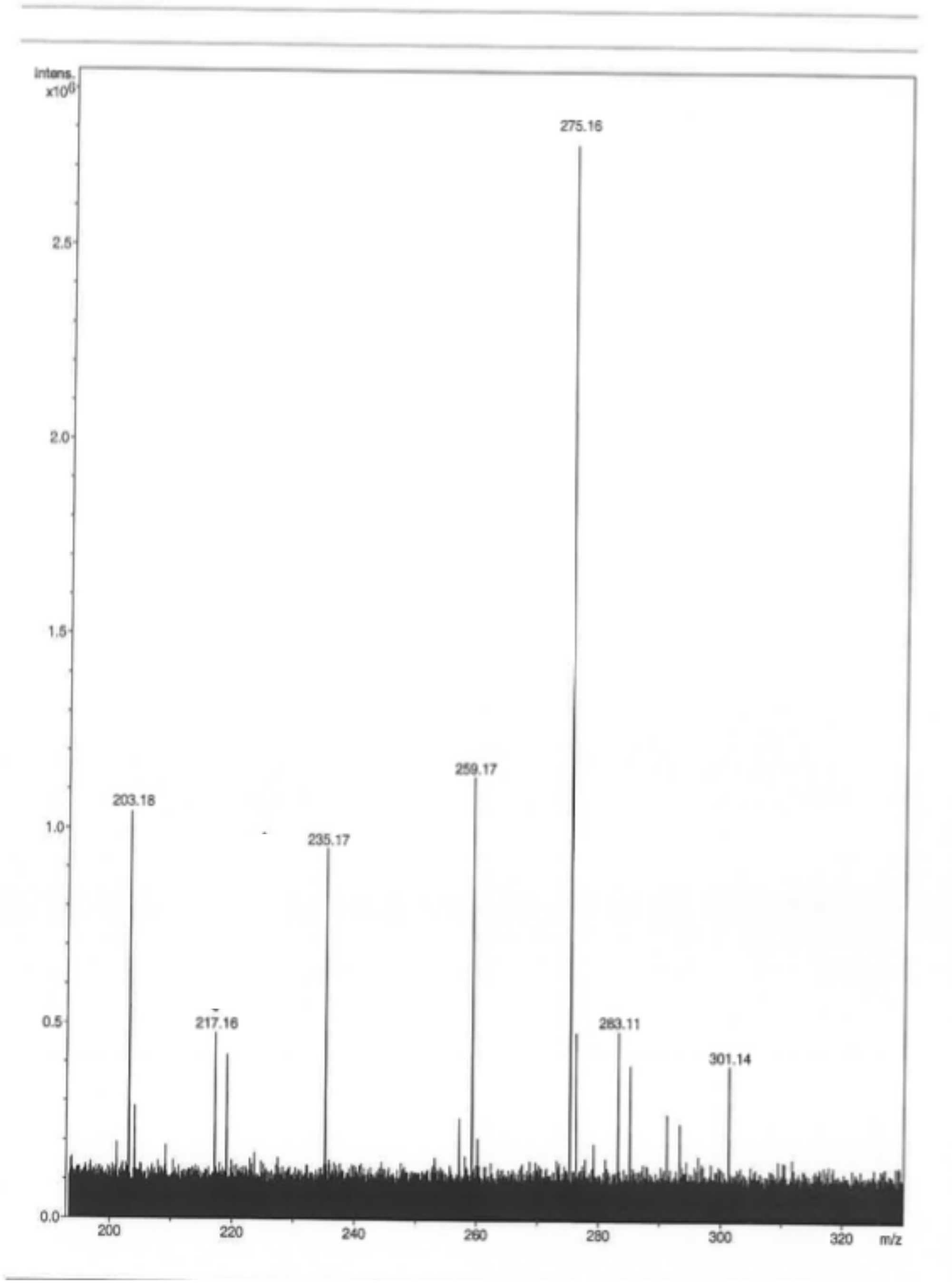

Figure S41. ^{1}H NMR spectrum (600 MHz, $CDCl_3$) of laurocarpane G (**7**).

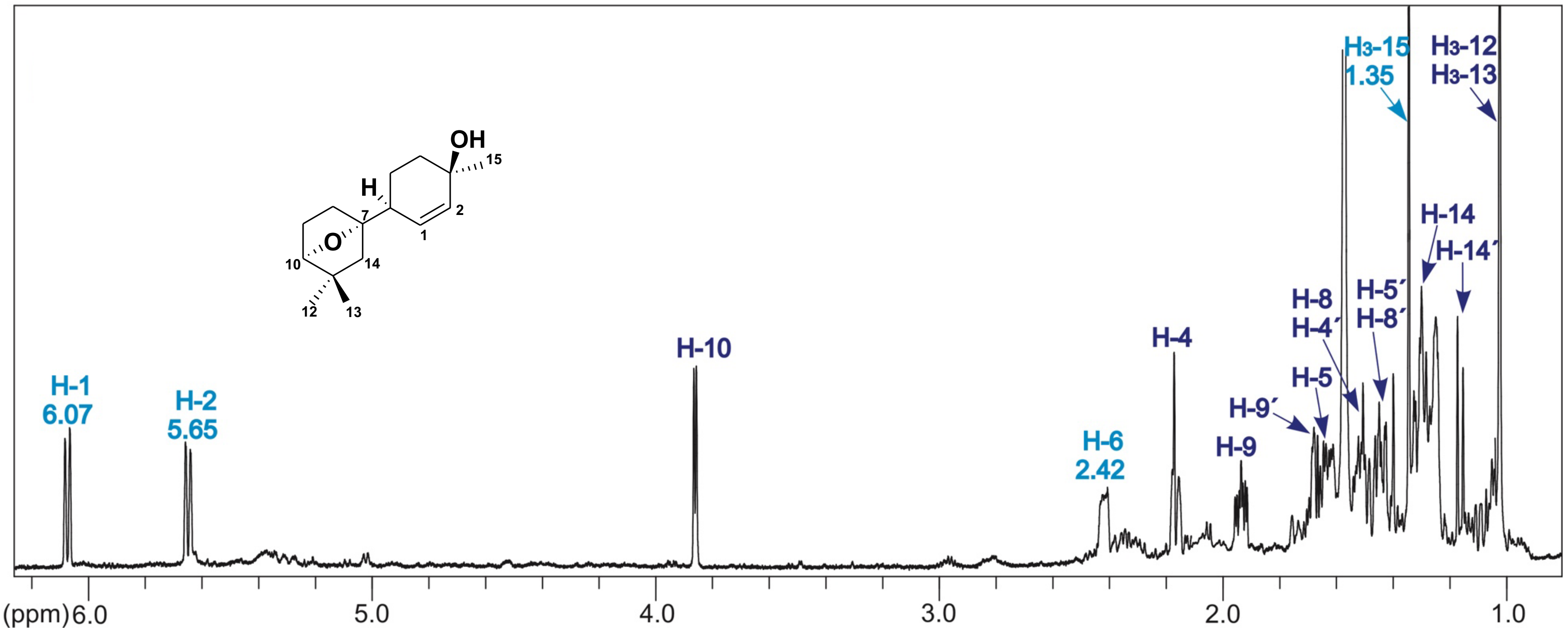

Figure S42. ^{13}C NMR spectrum (150 MHz, $CDCl_3$) of compound **7**.

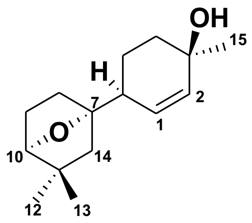

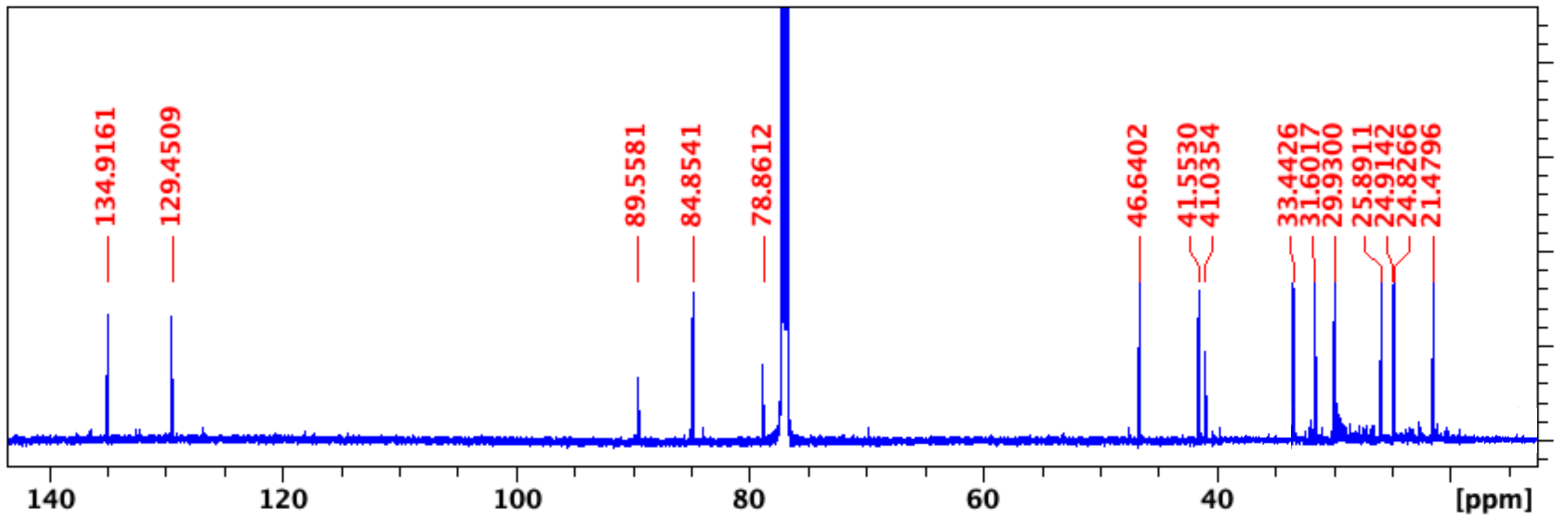

Figure S43. ^{1}H-^{1}H COSY NMR spectrum (600 MHz, $CDCl_3$) of compound **7**.

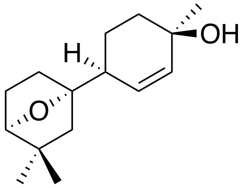

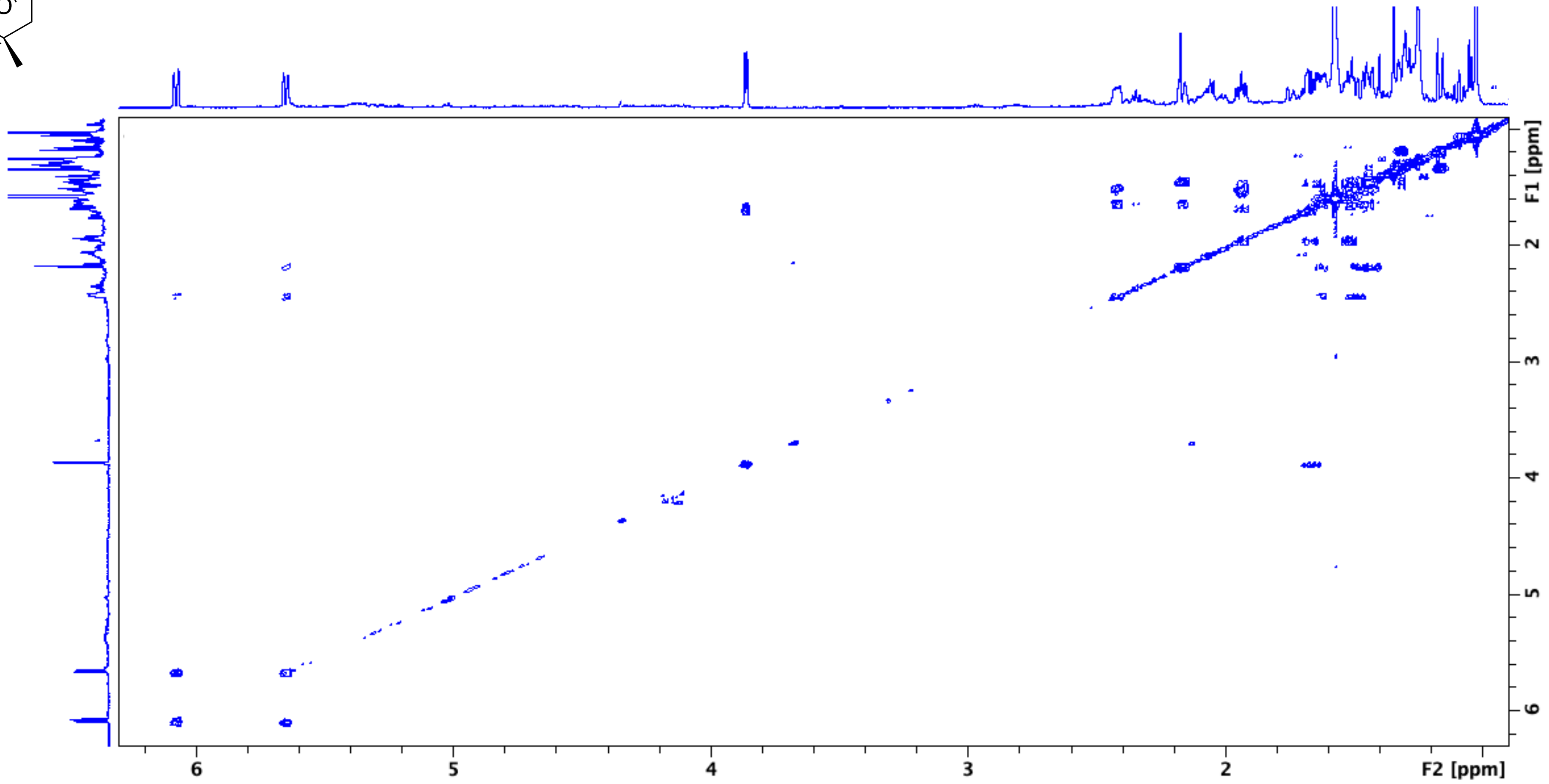

Figure S44. HSQC NMR spectrum (600 MHz, $CDCl_3$) of compound **7**.

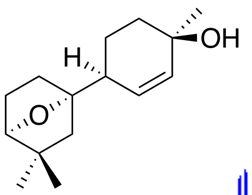

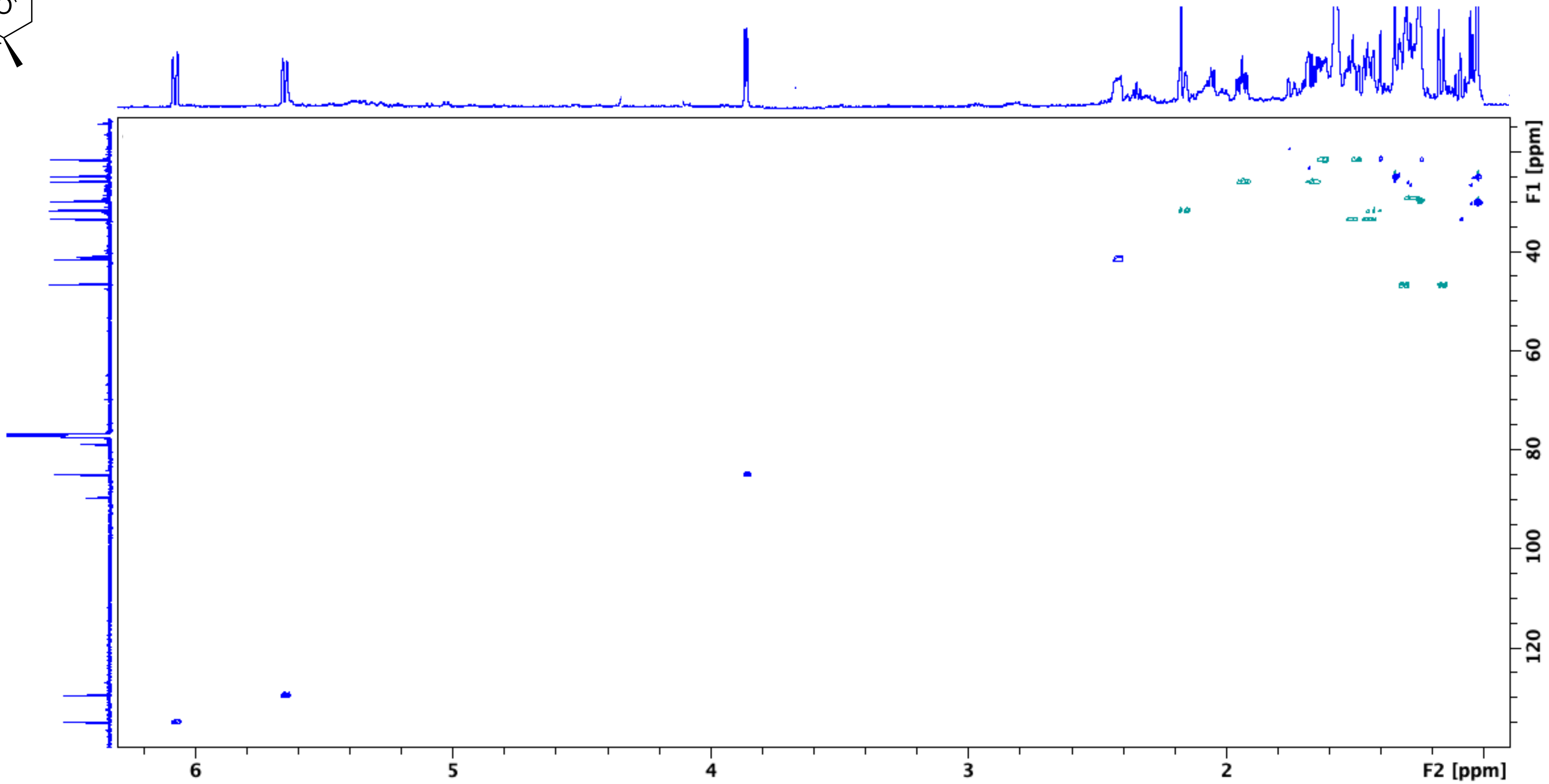

Figure S45. HMBC NMR spectrum (600 MHz, $CDCl_3$) of compound **7**.

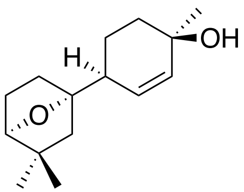

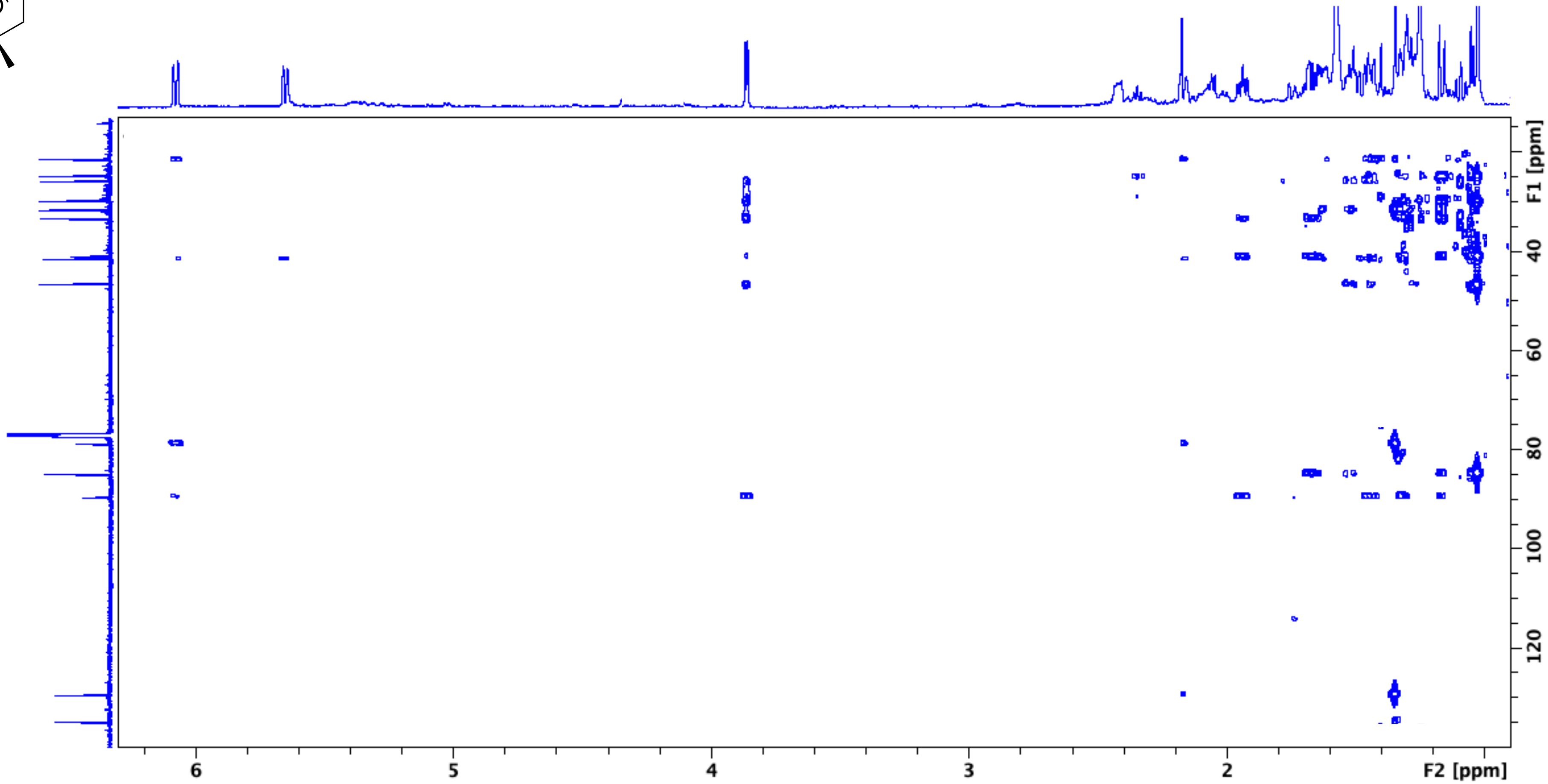

Figure S46. TROESY NMR spectrum (600 MHz, $CDCl_3$) of compound **7**.

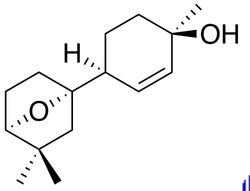

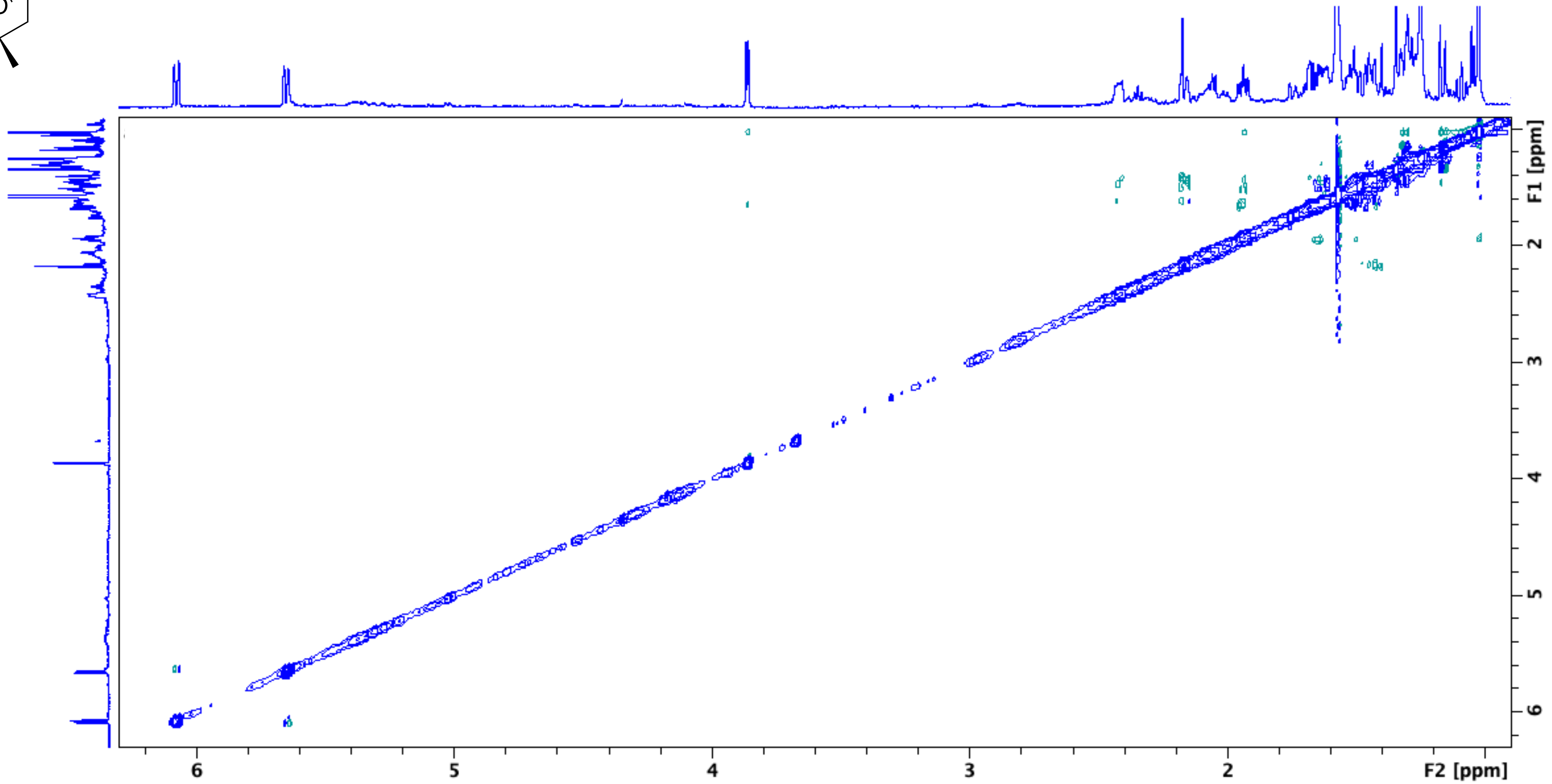

Figure S47. ESI-FTICR mass spectrum for compound **7**.

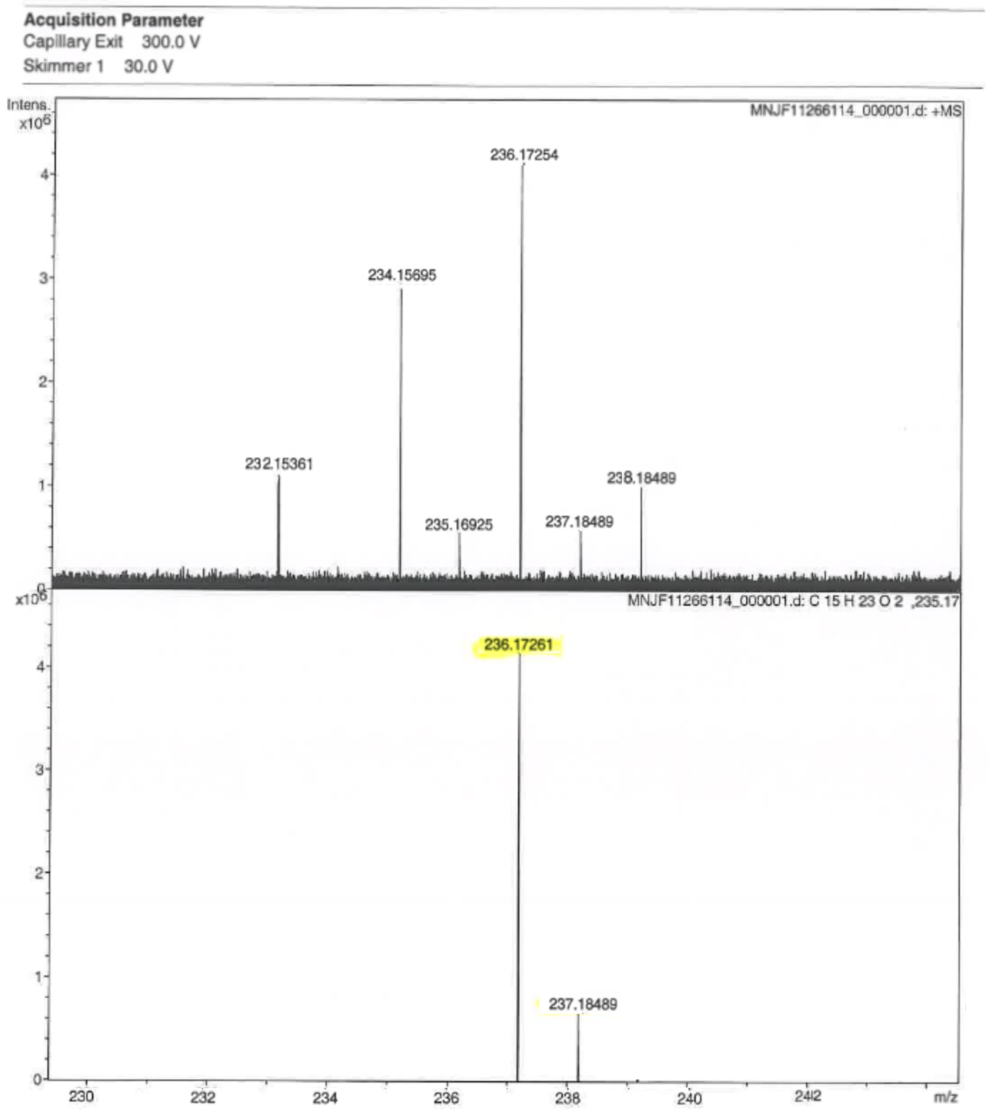

Computational Details

To facilitate the reproducibility of the calculations, representative Gaussian input files used for geometry optimizations of minima and transition states, as well as for single-point energy calculations, are provided below. Atomic coordinates have been omitted for brevity.

Geometry optimization and frequency calculation of a minimum:

```
%NPROCS=8
%Mem=8GB
%chk=minimum.chk
#PBE1PBE/def2SVP opt EmpiricalDispersion=GD3BJ pop=none

Title: Minimum Optimization

1 1
[Atomic coordinates]

--Link1--
%NPROCS=16
%mem=16GB
%chk=minimum.chk
#PBE1PBE/def2SVP EmpiricalDispersion=GD3BJ freq pop=(full,Hirshfeld) iop(6/7=3) geom=check guess=read

Title: Frequency calculation

1 1
```

Geometry optimization and frequency calculation of a transition state:

```
%NPROCS=8
%Mem=8GB
%chk=TS.chk
#PBE1PBE/def2SVP Opt=(TS,CalcFC,NoEigenTest,ModRedundant) EmpiricalDispersion=GD3BJ pop=none

Title: Transition-state optimization

1 1
[Atomic coordinates]

[ModRedundant specification]

--Link1--
%NPROCS=16
%mem=16GB
%chk=TS.chk
#PBE1PBE/def2SVP EmpiricalDispersion=GD3BJ freq pop=(full,Hirshfeld) iop(6/7=3) geom=check guess=read

Title: Frequency calculation
```

1 1

Single-point energy calculation:

%NPROCS=8
%Mem=8GB
%chk=single_point.chk
PBE1PBE/def2TZVPP EmpiricalDispersion=GD3BJ Pop=None SCRF=(SMD,Solvent=Water)

Title: Single-point energy calculation

1 1
[Atomic coordinates]

Cartesian coordinates and energies in au of DFT-optimized structures

39

(*S*)-*β*-bisabolene

C	-2.511747	0.729261	-1.170132
C	-3.682738	0.354840	-0.305394
C	-3.516518	0.102844	1.001604
C	-2.204326	0.164545	1.723744
C	-0.979215	0.273904	0.804215
C	-1.315679	1.214527	-0.366344
H	-2.819809	1.506846	-1.890459
H	-2.219613	1.032610	2.409514
H	-2.097663	-0.719032	2.375664
H	-1.544983	2.209422	0.053204
H	-0.445272	1.343466	-1.026013
C	-5.012648	0.249241	-0.983665
H	-5.309870	1.214643	-1.428126
H	-4.972577	-0.476199	-1.814788
H	-5.804044	-0.068978	-0.290044
C	-1.217466	-2.091368	-0.014558
H	-0.781723	-3.038629	-0.345986
H	-2.307709	-2.028532	0.030253
C	1.066784	-1.200397	0.309329
C	1.800134	-0.238205	-0.636206
H	1.331606	-2.236343	0.042545
H	1.457638	-1.035332	1.330503
C	3.273202	-0.495874	-0.671060
H	1.383403	-0.362000	-1.651448
H	1.595600	0.802282	-0.343918
H	-0.173284	0.751836	1.386956
C	4.256077	0.259026	-0.153035
C	-0.433352	-1.063768	0.336272
C	5.691732	-0.165157	-0.259488
H	6.289750	0.593329	-0.793753
H	6.147855	-0.272498	0.739869
H	5.799307	-1.121856	-0.789846
C	4.038870	1.559535	0.562474
H	4.577939	2.378032	0.055431
H	2.981642	1.844902	0.635419
H	4.446045	1.508616	1.586640
H	3.569595	-1.435411	-1.155843
H	-4.394177	-0.162704	1.602780
H	-2.228320	-0.146186	-1.785083

Sum of electronic and zero-point Energies= -584.564141
Sum of electronic and thermal Free Energies= -584.610235

40

INT1

C	3.967035	0.322528	0.511547
C	3.518194	-1.076750	0.186440
C	2.612620	-1.313175	-0.777484
C	1.971255	-0.245631	-1.614785
C	2.074196	1.158461	-1.005534
C	3.515227	1.363298	-0.505426
H	5.068149	0.333205	0.580830
H	2.457152	-0.224475	-2.607251
H	0.922314	-0.508676	-1.840604
H	4.170969	1.302441	-1.389715
H	3.631417	2.378485	-0.097947
C	4.141119	-2.176052	0.987461
H	5.232367	-2.202349	0.831566
H	3.988208	-2.014042	2.068105
H	3.734016	-3.162044	0.724085
C	0.459353	2.707103	0.049085
H	-0.203875	3.027735	0.859887
H	0.670964	3.443174	-0.731970
C	0.691088	0.530057	1.164782
C	-0.283525	-0.589479	0.750854
H	0.244988	1.100121	1.994264
H	1.584445	0.032435	1.563862
C	-1.358189	-0.039138	-0.135391
H	0.252448	-1.372748	0.196317
H	-0.708251	-1.062296	1.64777439
H	1.914435	1.887876	-1.814626
C	-2.483348	0.778461	0.327990
C	1.047417	1.500141	0.057542
C	-3.084838	1.761898	-0.607848
H	-4.152020	1.923625	-0.406563
H	-2.559686	2.715465	-0.413766
H	-2.927129	1.503435	-1.662371
C	-2.790394	0.913330	1.773718
H	-3.865152	1.060231	1.944196
H	-2.420382	0.081511	2.383340
H	-2.282381	1.837020	2.107167
H	-1.043623	0.189447	-1.159836
H	2.351624	-2.352361	-1.009485
H	3.632127	0.585625	1.533044
Br	-2.962182	-1.259233	-0.350800

Sum of electronic and zero-point Energies= -3157.852151
Sum of electronic and thermal Free Energies= -3157.898925

2

OH^-

O	-0.000000	-0.000000	0.108959
H	0.000000	0.000000	-0.871673

Sum of electronic and zero-point Energies= -75.571774
Sum of electronic and thermal Free Energies= -75.588057

4

H_3O^+

O	-0.000001	0.000001	-0.074474
H	0.601544	0.726100	0.198598
H	0.328066	-0.883997	0.198595
H	-0.929600	0.157887	0.198600

Sum of electronic and zero-point Energies= -76.528495
Sum of electronic and thermal Free Energies= -76.547643

3

H_2O

O	0.000000	0.000000	0.119672
H	-0.000000	0.754153	-0.478688
H	-0.000000	-0.754153	-0.478688

Sum of electronic and zero-point Energies= -76.255015
Sum of electronic and thermal Free Energies= -76.272666

2

HBr

Br	-0.000000	-0.000000	0.039503
H	0.000000	0.000000	-1.382597

Sum of electronic and zero-point Energies= -2574.138625
Sum of electronic and thermal Free Energies= -2574.157848

3

HOBr

H	-0.921909	1.671362	0.000000
O	0.021440	1.452642	0.000000
Br	0.021440	-0.379786	-0.000000

Sum of electronic and zero-point Energies= -2649.157347
Sum of electronic and thermal Free Energies= -2649.181507

40

INT2

C	-4.511403	0.367493	-0.604694
C	-4.216645	-0.720581	0.389834
C	-3.109460	-0.607824	1.148753

C	-2.262185	0.609504	1.115698
C	-2.147416	1.265585	-0.356268
C	-3.229372	0.786881	-1.322280
H	-4.963116	1.234687	-0.089462
H	-2.652464	1.437830	1.731140
H	-1.271915	0.401635	1.565633
H	-3.428816	1.598838	-2.037161
H	-2.873893	-0.068847	-1.913998
C	-5.151468	-1.876083	0.477865
H	-6.166523	-1.524847	0.727811
H	-5.233631	-2.386058	-0.496614
H	-4.840981	-2.607648	1.235454
C	0.337822	1.708952	-0.225486
H	0.014871	2.568631	0.383490
H	0.505251	2.142685	-1.240054
C	-0.500551	-0.559548	-0.981417
C	0.832760	-1.158404	-0.542410
H	-0.523730	-0.435353	-2.087256
H	-1.358241	-1.222561	-0.771039
C	1.953568	-0.133262	-0.576492
H	0.735981	-1.572468	0.472673
H	1.079945	-2.006497	-1.195088
C	1.664871	1.109317	0.288246
C	-0.785483	0.801296	-0.501770
C	2.750527	2.168459	0.105553
H	2.893658	2.419951	-0.957290
H	2.488035	3.091729	0.643952
H	3.710605	1.808567	0.499008
C	1.525450	0.768046	1.772223
H	1.265223	1.667236	2.350666
H	0.758026	0.003917	1.970099
H	2.475726	0.379872	2.164010
H	2.125862	0.197154	-1.613548
H	-2.841304	-1.393674	1.860422
H	-5.255026	0.029953	-1.340834
Br	3.618002	-1.008528	-0.078857
H	-2.179711	2.354761	-0.226162

Sum of electronic and zero-point Energies= -3157.894872
Sum of electronic and thermal Free Energies= -3157.939956

43
INT3B

C	-4.283106	-0.076016	0.201497
C	-3.569063	-1.400872	0.260065
C	-2.367608	-1.396571	0.856938
C	-1.863582	-0.121760	1.462558
C	-1.999993	1.093102	0.516509
C	-3.326165	1.018509	-0.283446
H	-4.665750	0.190913	1.203085
H	-2.468028	0.119066	2.354588
H	-0.837959	-0.212037	1.830817
H	-3.845320	1.992159	-0.256945
H	-3.127026	0.777066	-1.346818
C	-4.225083	-2.606500	-0.323158
H	-5.192591	-2.798433	0.169997
H	-4.445501	-2.462156	-1.394267
H	-3.601637	-3.504202	-0.213923
C	0.475083	1.902865	0.191773
H	0.215249	2.526276	1.063328
H	0.856212	2.580467	-0.589910
C	-0.485779	0.178861	-1.401695
C	0.612530	-0.765050	-0.912138
H	-0.135014	0.657898	-2.329619
H	-1.392517	-0.394538	-1.640700
C	1.879585	0.005895	-0.591989
H	0.279575	-1.341906	-0.037244
H	0.811559	-1.499039	-1.705154
C	1.697908	1.008689	0.561708
C	-0.782953	1.271726	-0.395095
C	2.897935	1.957478	0.639943
H	3.057455	2.491176	-0.310247
H	2.754174	2.704972	1.435040
H	3.812806	1.393697	0.868335
C	1.557905	0.312398	1.918681
H	1.124862	0.991952	2.667841
H	0.952374	-0.602662	1.882796
H	2.547996	0.006352	2.280547
H	2.207224	0.555414	-1.488591
H	-1.783095	-2.315488	0.959178
H	-5.159353	-0.117665	-0.461610
Br	3.330875	-1.247742	-0.254248
H	-2.025492	1.989374	1.159992
O	-1.288783	2.500395	-1.364095
H	-1.306048	3.340028	-0.868721
H	-2.210206	2.302631	-1.633257

Sum of electronic and zero-point Energies= -3234.155846
Sum of electronic and thermal Free Energies= -3234.201458

40
INT3

C	-4.313658	0.814955	0.870501
C	-4.981695	-0.117197	-0.105968
C	-4.238363	-1.019732	-0.763063
C	-2.779252	-1.195270	-0.504807
C	-2.081333	-0.112232	0.177336
C	-2.856408	1.086980	0.493954
H	-4.375955	0.401477	1.893372
H	-2.568220	-2.103454	0.108830
H	-2.189377	-1.439371	-1.414685
H	-2.332318	1.759548	1.187649
H	-2.870941	1.599234	-0.499844
C	-6.460189	-0.001758	-0.260630
H	-6.959526	-0.118069	0.715715
H	-6.737813	0.996075	-0.637941
H	-6.861904	-0.758865	-0.946394
C	0.034810	0.830961	-0.536813
H	-0.434611	1.823107	-0.468432
H	-0.118661	0.458675	-1.563682
C	0.026512	-1.538442	0.351469
C	1.520590	-1.422384	0.628029
H	-0.121208	-1.953299	-0.660295
H	-0.430554	-2.249821	1.058604
C	2.170647	-0.422913	-0.314870
H	1.696767	-1.124784	1.673712
H	1.996013	-2.405396	0.503527
C	1.545539	0.984642	-0.253285
C	-0.650975	-0.178550	0.454616
C	2.102656	1.867039	-1.373562
H	1.954542	1.405749	-2.362246
H	1.615498	2.853689	-1.375257
H	3.181654	2.020445	-1.231643
C	1.764567	1.670317	1.093690
H	1.271264	2.654219	1.107446
H	1.387702	1.087520	1.948100
H	2.837441	1.830223	1.268299
H	2.084209	-0.790357	-1.349644
H	-4.700833	-1.719427	-1.463226
H	-4.843088	1.778261	0.907047
Br	4.091374	-0.392795	0.011549
H	-0.498857	0.263963	1.457187

Sum of electronic and zero-point Energies= -3157.895957
Sum of electronic and thermal Free Energies= -3157.941314

42
INT4B

C	-4.265318	-0.127676	0.126297
C	-3.485297	-1.403592	0.295814
C	-2.318929	-1.299796	0.948894
C	-1.902812	0.041264	1.472637
C	-2.047521	1.164687	0.430683
C	-3.362917	1.006984	-0.368979

H	-4.717094	0.150327	1.097723
H	-2.559070	0.302750	2.323727
H	-0.890804	0.025016	1.885953
H	-3.926982	1.952079	-0.364339
H	-3.122514	0.821711	-1.425564
C	-4.035157	-2.677240	-0.256219
H	-5.024848	-2.901897	0.177309
H	-4.183313	-2.603549	-1.347444
H	-3.370986	-3.530194	-0.055606
C	0.436107	1.927055	0.170287
H	0.151073	2.524712	1.054185
H	0.824637	2.645695	-0.567369
C	-0.508341	0.195250	-1.430502
C	0.556640	-0.750027	-0.896089
H	-0.152757	0.632428	-2.377478
H	-1.422225	-0.367057	-1.670810
C	1.823502	0.019283	-0.582876
H	0.208567	-1.286653	-0.000907
H	0.770580	-1.519782	-1.651683
C	1.646820	1.030778	0.558357
C	-0.843207	1.386267	-0.525728
C	2.863164	1.959188	0.637653
H	3.031316	2.479536	-0.318217
H	2.713060	2.723242	1.416546
H	3.772903	1.392581	0.884013
C	1.510369	0.336626	1.917177
H	1.107709	1.033474	2.667840
H	0.864803	-0.550676	1.889139
H	2.494463	-0.005857	2.265714
H	2.156553	0.555514	-1.484420
H	-1.692065	-2.180002	1.125891
H	-5.106119	-0.265860	-0.571353
Br	3.298038	-1.239340	-0.243572
H	-2.106345	2.107262	1.007375
O	-1.259838	2.404554	-1.437953
H	-1.575774	3.151374	-0.916014

Sum of electronic and zero-point Energies= -3233.834533
Sum of electronic and thermal Free Energies= -3233.879928

43
INT4

C	-3.447056	-1.090746	-0.980932
C	-3.821207	-1.152556	0.474734
C	-3.589863	-0.117968	1.296778
C	-2.948941	1.174721	0.867974
C	-2.188336	0.970332	-0.427682
C	-3.083531	0.315860	-1.453135
H	-4.281333	-1.466980	-1.594537
H	-3.733469	1.944267	0.721192
H	-2.291851	1.552153	1.666099
H	-4.006912	0.912291	-1.543042
H	-2.606743	0.295009	-2.442005
C	-4.441648	-2.428055	0.944809
H	-5.392612	-2.614789	0.419487
H	-3.786976	-3.286888	0.722762
H	-4.640207	-2.415776	2.024414
C	0.162464	1.271793	0.524899
H	-0.284195	1.625204	1.467556
H	0.421874	2.175440	-0.075610
C	-0.122006	-0.075095	-1.559975
C	1.225154	-0.737099	-1.286628
H	0.021404	0.801019	-2.218272
H	-0.755127	-0.781686	-2.114973
C	2.135276	0.160721	-0.466250
H	1.075245	-1.692944	-0.759958
H	1.717462	-0.984208	-2.237850
C	1.518818	0.613543	0.871925
C	-0.793571	0.365064	-0.259188
C	2.397818	1.671721	1.536256
H	2.572536	2.529237	0.866452
H	1.929119	2.045392	2.459574
H	3.377649	1.250906	1.800356
C	1.299394	-0.546309	1.844504
H	0.803635	-0.187951	2.759650
H	0.688333	-1.358429	1.424778
H	2.264691	-0.983803	2.133883
H	2.381970	1.061522	-1.050927
H	-3.872815	-0.187257	2.350541
H	-2.615503	-1.793850	-1.169425
Br	3.858106	-0.721204	-0.227473
H	-0.984458	-0.528406	0.359173
O	-1.892687	2.398965	-1.014781
H	-1.080902	2.743676	-0.580391
H	-2.629643	3.021707	-0.877666

Sum of electronic and zero-point Energies= -3234.175659
Sum of electronic and thermal Free Energies= -3234.221036

42
Laurocarpane A

C	-3.425058	-0.926018	-1.126199
C	-3.808358	-1.167365	0.308815
C	-3.599984	-0.230171	1.245801
C	-2.973867	1.105473	0.976115
C	-2.179762	1.145346	-0.336599
C	-3.079587	0.528115	-1.413045
H	-4.247683	-1.256141	-1.784471
H	-3.761478	1.882182	0.918164
H	-2.338852	1.410454	1.821181
H	-4.012041	1.121168	-1.436745
H	-2.615927	0.642478	-2.403369
C	-4.406345	-2.502798	0.622810
H	-5.341396	-2.661359	0.058761
H	-3.723630	-3.318525	0.328569
H	-4.627460	-2.611964	1.693850
C	0.105358	1.086349	0.787102
H	-0.371041	1.173511	1.775819
H	0.281258	2.117333	0.439322
C	-0.112451	0.267775	-1.551874
C	1.239103	-0.425372	-1.417039
H	0.021767	1.277926	-1.974438
H	-0.721415	-0.296767	-2.274030
C	2.108230	0.276603	-0.392259
H	1.101636	-1.478504	-1.122401
H	1.758210	-0.439649	-2.387050
C	1.463072	0.381830	0.997935
C	-0.831105	0.402017	-0.209364
C	2.315293	1.257954	1.914537
H	2.477856	2.253227	1.471198
H	1.813279	1.398947	2.884355
H	3.298811	0.801876	2.099093
C	1.264179	-0.985171	1.654046
H	0.760711	-0.862174	2.625439
H	0.648297	-1.664285	1.047654
H	2.231287	-1.478318	1.829219
H	2.340559	1.293820	-0.742281
H	-3.899123	-0.441930	2.278382
H	-2.574944	-1.586572	-1.383781
Br	3.875772	-0.592821	-0.343659
H	-1.066623	-0.607265	0.167786
O	-1.859061	2.479299	-0.681223
H	-2.683896	2.957471	-0.817047

Sum of electronic and zero-point Energies= -3233.858748
Sum of electronic and thermal Free Energies= -3233.903967

40
Laurocarpane F

C	3.175863	-0.928930	-0.754843
C	3.426851	0.210576	0.196840
C	2.614302	0.298341	1.259735
C	1.558978	-0.745554	1.469261
C	0.785849	-1.089904	0.175688

C	1.683461	-1.040484	-1.076430
H	3.532225	-1.872998	-0.300146
H	2.044019	-1.670744	1.831286
H	0.858506	-0.452975	2.265702
H	1.492228	-1.921867	-1.705767
H	1.398630	-0.175050	-1.693624
C	4.531644	1.171866	-0.096897
H	5.503199	0.651754	-0.157504
H	4.380134	1.661573	-1.074478
H	4.609118	1.954093	0.672032
C	-1.579592	-0.456956	1.035330
H	-1.467184	0.143101	1.949916
H	-1.604937	-1.517269	1.330483
C	-0.218843	1.318879	-0.071471
C	-1.439369	1.755905	-0.906014
H	0.727698	1.546501	-0.579403
H	-0.176170	1.786011	0.922722
C	-2.169001	0.419742	-1.084148
H	-2.052067	2.533581	-0.431100
H	-1.130751	2.133873	-1.891064
C	-2.848252	-0.069058	0.223845
C	-0.443873	-0.208406	0.020543
C	-3.676705	-1.311149	-0.100176
H	-3.070911	-2.035071	-0.664175
H	-4.029870	-1.799703	0.821986
H	-4.560830	-1.052732	-0.705554
C	-3.718600	0.970508	0.917408
H	-4.210558	0.532026	1.799897
H	-3.138128	1.838355	1.263824
H	-4.512783	1.338152	0.246978
H	-2.823614	0.364942	-1.965644
H	2.730998	1.107181	1.988385
H	3.753737	-0.801489	-1.683764
H	0.399836	-2.116525	0.286437
O	-1.093598	-0.493371	-1.222431

Sum of electronic and zero-point Energies= -659.690968
Sum of electronic and thermal Free Energies= -659.732896

40
TS1

C	-4.458566	0.478900	0.084542
C	-4.010360	-0.951640	0.224159
C	-2.819396	-1.151279	0.810480
C	-2.062274	0.029087	1.342148
C	-2.006284	1.227907	0.361844
C	-3.319248	1.341934	-0.460770
H	-4.783753	0.863559	1.069051
H	-2.568110	0.390956	2.255587
H	-1.053493	-0.253920	1.674586
H	-3.619229	2.398439	-0.520733
H	-3.131081	1.024975	-1.499656
C	-4.892477	-2.042023	-0.284289
H	-5.873658	-2.013200	0.218475
H	-5.093351	-1.918349	-1.361851
H	-4.454111	-3.036647	-0.122638
C	-0.122428	2.298227	-0.864856
H	-0.298463	3.235469	-0.327551
H	0.601758	2.329847	-1.685374
C	-0.573632	-0.083549	-1.359002
C	0.567270	-0.905411	-0.738669
H	-0.318299	0.144919	-2.406020
H	-1.468207	-0.723365	-1.375937
C	1.829223	-0.089967	-0.699563
H	0.299393	-1.269799	0.262751
H	0.754503	-1.793081	-1.361249
C	2.161893	0.846812	0.384033
C	-0.854075	1.197231	-0.610285
C	3.040213	2.006558	0.105320
H	2.431161	2.915937	0.247436
H	3.854499	2.067099	0.842705
H	3.448418	2.008101	-0.912574
C	1.624595	0.698173	1.754680
H	0.835623	1.461206	1.869426
H	1.182965	-0.284796	1.949225
H	2.397187	0.922513	2.503491
H	2.136313	0.282122	-1.685045
H	-2.428448	-2.162921	0.959659
H	-5.332642	0.558246	-0.578934
Br	3.421494	-1.105599	-0.036531
H	-1.898002	2.142897	0.965124

Sum of electronic and zero-point Energies= -3157.848238
Sum of electronic and thermal Free Energies= -3157.894183

43
TS2B

C	-4.282270	-0.137706	0.139251
C	-3.537135	-1.441516	0.249652
C	-2.362553	-1.397686	0.897863
C	-1.919005	-0.102773	1.500623
C	-2.049091	1.099747	0.511842
C	-3.343370	0.980524	-0.324920
H	-4.715055	0.127361	1.120894
H	-2.572999	0.157540	2.351152
H	-0.910290	-0.158471	1.921922
H	-3.880398	1.942755	-0.310658
H	-3.098831	0.763108	-1.379037
C	-4.132887	-2.673240	-0.342486
H	-5.116031	-2.883452	0.110617
H	-4.310361	-2.548749	-1.423989
H	-3.491605	-3.552322	-0.192651
C	0.427833	1.855205	0.296633
H	0.157687	2.442043	1.188095
H	0.783006	2.563211	-0.468563
C	-0.497934	0.190379	-1.363174
C	0.633952	-0.754599	-0.933512
H	-0.157328	0.730688	-2.260416
H	-1.394776	-0.385100	-1.626751
C	1.881706	0.030851	-0.579868
H	0.318171	-1.385241	-0.089674
H	0.838672	-1.436764	-1.770167
C	1.678306	0.968767	0.623050
C	-0.787152	1.198796	-0.296589
C	2.854993	1.937624	0.757385
H	3.010673	2.517839	-0.165623
H	2.687625	2.644147	1.584420
H	3.779490	1.382887	0.969224
C	1.534982	0.201714	1.938831
H	1.135921	0.853482	2.730434
H	0.895405	-0.687693	1.861300
H	2.519967	-0.154687	2.267699
H	2.193364	0.632314	-1.448255
H	-1.756272	-2.297467	1.037210
H	-5.129694	-0.216606	-0.557133
Br	3.361760	-1.204426	-0.307929
H	-2.093188	2.003716	1.139880
O	-1.296397	2.674815	-1.435057
H	-1.440862	3.479569	-0.911611
H	-2.144976	2.485752	-1.867827

Sum of electronic and zero-point Energies= -3234.156724
Sum of electronic and thermal Free Energies= -3234.202591

40
TS2

C	-3.780095	1.229577	0.276647
C	-4.532203	-0.018356	0.647913
C	-4.126289	-1.219060	0.213343
C	-2.892479	-1.464717	-0.606339
C	-2.040188	-0.238271	-0.785510
C	-2.836265	1.028205	-0.905280
H	-3.224384	1.607353	1.155248
H	-2.318389	-2.297165	-0.174841

H	-3.155993	-1.801599	-1.629383
H	-2.212569	1.912775	-1.073376
H	-3.435016	0.882378	-1.822802
C	-5.734717	0.162069	1.516099
H	-5.467708	0.687085	2.448174
H	-6.491934	0.783857	1.010827
H	-6.196837	-0.797538	1.782166
C	0.218350	0.895024	-1.233719
H	-0.345029	1.834587	-1.197070
H	0.559830	0.776323	-2.278024
C	0.123984	-1.596251	-0.984389
C	1.403557	-1.551053	-0.146746
H	0.381568	-1.733737	-2.051300
H	-0.491737	-2.459970	-0.709112
C	2.235090	-0.315796	-0.448101
H	1.151774	-1.582354	0.925034
H	1.993984	-2.455632	-0.348018
C	1.451384	1.002900	-0.300523
C	-0.637637	-0.304194	-0.964462
C	2.287899	2.187739	-0.777212
H	2.637609	2.040855	-1.810882
H	1.702508	3.118918	-0.742657
H	3.171094	2.318000	-0.137132
C	0.977687	1.246255	1.132211
H	0.400549	2.181690	1.193300
H	0.348996	0.431609	1.531762
H	1.836441	1.336398	1.811921
H	2.620243	-0.371604	-1.478416
H	-4.698887	-2.112561	0.477586
H	-4.493380	2.029934	0.023352
Br	3.835603	-0.356020	0.658830
H	-1.227291	-0.133318	0.230978

Sum of electronic and zero-point Energies= -3157.889238
Sum of electronic and thermal Free Energies= -3157.934022

43
TS3

C	-3.843307	-1.372195	-0.408248
C	-4.556129	-0.584730	0.654632
C	-4.074301	0.601761	1.045956
C	-2.770906	1.184311	0.557342
C	-2.054984	0.302898	-0.381868
C	-2.928661	-0.474893	-1.270906
H	-4.569185	-1.842568	-1.089736
H	-2.956089	2.112704	-0.022320
H	-2.129781	1.484120	1.399262
H	-3.585218	0.253752	-1.775067
H	-2.380052	-1.050693	-2.023151
C	-5.792925	-1.193503	1.231328
H	-6.562521	-1.329949	0.454191
H	-5.577745	-2.193764	1.641966
H	-6.216342	-0.576271	2.034145
C	0.255689	1.195173	0.207543
H	-0.217498	1.807435	0.988413
H	0.358313	1.835057	-0.683934
C	0.051317	-0.833605	-1.275794
C	1.457354	-1.245729	-0.850955
H	0.094576	-0.234755	-2.201839
H	-0.527932	-1.740056	-1.503199
C	2.291529	-0.049345	-0.427230
H	1.406804	-1.973076	-0.025191
H	1.954187	-1.763426	-1.683561
C	1.653112	0.777900	0.705278
C	-0.631899	0.014797	-0.209999
C	2.452917	2.057811	0.947441
H	2.549779	2.649921	0.023725
H	1.963149	2.685676	1.707348
H	3.465401	1.821805	1.302743
C	1.537425	-0.005269	2.012336
H	1.056023	0.612355	2.785997
H	0.956322	-0.935976	1.913283
H	2.532882	-0.290424	2.380173
H	2.442944	0.616270	-1.291635
H	-4.593290	1.190292	1.807214
H	-3.274774	-2.206146	0.041407
Br	4.091530	-0.657552	0.014436
H	-0.768438	-0.615693	0.715111
O	-1.736354	1.980423	-2.174327
H	-1.684400	1.749255	-3.110160
H	-1.327230	2.852802	-2.116957

Sum of electronic and zero-point Energies= -3234.168411
Sum of electronic and thermal Free Energies= -3234.215766
